# Dual Covalent Targeting of STING Cysteines 292/309 Disrupts Functional Oligomerization and Enables Potent Antagonist Development

**DOI:** 10.1002/advs.202522764

**Published:** 2026-03-24

**Authors:** Yuxuan Zhao, Ling Huang, Wenjing Qin, Bin Zhang, Yang Yang, Xue Chen, Xiaoquan Wang, Weilin Zhou, Feiyang Chen, Zhenyu Li, Liyuan Le, Yiqiu Zhang, Zhen Xiang, Lu Zhang, Fei Wang, Dan Lei, Zi‐zhe Cai, Ying Gao, Yong Chen, Xuecen Wang, Junmin Quan, Shuixing Zhang, Xianzhang Bu, Xin Yue

**Affiliations:** ^1^ State Key Laboratory of Bioactive Molecules and Druggability Assessment Department of Radiology The First Affiliated Hospital Jinan University Guangzhou Guangdong China; ^2^ State Key Laboratory of Anti‐Infective Drug Discovery and Development School of Pharmaceutical Sciences Sun Yat‐Sen University Guangzhou Guangdong China; ^3^ Department of Radiation Oncology The First Affiliated Hospital Sun Yat‐sen University Guangzhou Guangdong China; ^4^ State Key Laboratory of Oncology in South China Guangdong Provincial Clinical Research Center For Cancer Sun Yat‐sen University Cancer Center Guangzhou Guangdong China; ^5^ Laboratory of Chemical Oncogenomics Guangdong Provincial Key Laboratory of Chemical Genomics Peking University Shenzhen Graduate School Shenzhen Guangdong China; ^6^ Jiangsu Synthgene Biotechnology Co., Ltd. Yixing Jiangsu China; ^7^ China‐Malaysia Belt and Road Joint Laboratory on Oil Processing and Safety Jinan University Guangzhou Guangdong China

**Keywords:** dual cysteines covalent modification, drug development, STING‐driven disease, P005091 (NTP1), STING functional oligomerization

## Abstract

Dysregulated STING activation is a well‐established driver of pathological inflammation in autoimmune and autoinflammatory diseases, underscoring the need for targeted therapeutic inhibition. Current STING antagonist development has predominantly relied on phenotypic screening strategies. In contrast, we introduce a rational design strategy that directly disrupts STING signaling at its structural origin by covalently targeting cysteine residues within the C‐terminal domain (CTD) to prevent functional oligomerization. Through covalent warhead repurposing, we identified P005091, previously known as a USP7 inhibitor, as a STING antagonist that operates via a non‐classical nucleophilic displacement mechanism. Mechanistic investigation demonstrated that inhibition by P005091 depends on its concurrent engagement of Cys292 and Cys309, as evidenced by the fact that its activity to block STING oligomerization was abolished only by the C292A/C309A double mutation. Functionally, P005091 potently suppressed STING signaling and type I interferon responses in vitro and in vivo. Structure‐guided optimization yielded the advanced compounds NTP14 and NTP16, which exhibit markedly enhanced cellular potency and robust efficacy in ameliorating type I interferon‐driven pathology in multiple preclinical models, including DSS‐induced colitis. Our work establishes dual covalent CTD targeting as a transformative strategy for STING antagonist development and opens a new therapeutic avenue for quenching STING‐driven inflammation at its source.

## Graphical abstract

We report a rational design strategy for STING antagonists by dual covalent targeting of Cys292/309 in its C‐terminal domain, directly preventing functional oligomerization. Through covalent warhead repurposing, we identified P005091 and revealed its unique dual‐cysteine mechanism. Optimized leads NTP14/16 show potent cellular & in vivo efficacy, suppressing interferon responses and ameliorating colitis, offering a novel strategy against STING‐driven inflammation.

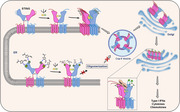

## 1 Introduction

The stimulator of interferon genes (STING) pathway serves as a critical sentinel of cellular defense, detecting cytosolic double‐stranded DNA (dsDNA) arising from microbial invasion, genomic instability, or mitochondrial stress [1, 2, 3]. Activation of STING by its endogenous ligand, 2’3’‐cyclic GMP‐AMP (cGAMP) synthesized by cyclic GMP‐AMP synthase (cGAS), initiates a potent innate immune cascade culminating in the production of type I interferons (I‐IFN) and pro‐inflammatory cytokines [4, 5]. While indispensable for host defense against pathogens and tumor surveillance, dysregulated or persistent STING signaling is a well‐established driver of pathological inflammation in a spectrum of conditions, including systemic lupus erythematosus (SLE), Aicardi‐Goutières syndrome (AGS), non‐alcoholic steatohepatitis (NASH), pulmonary fibrosis, and notably, inflammatory bowel disease (IBD) [6, 7, 8, 9]. Consequently, the development of potent and selective STING inhibitors represents a major therapeutic goal for ameliorating STING‐dependent immunopathology.

STING activation is a multi‐step, conformationally dynamic process. cGAMP binding induces profound structural rearrangements within the STING dimer, triggering its functional oligomerization in the endoplasmic reticulum (ER) [10, 11]. This oligomerization, governed primarily by the cytosolic C‐terminal domain (CTD), is the essential initial step that enables subsequent trafficking to the Golgi apparatus, recruitment and activation of TANK‐binding kinase 1 (TBK1), phosphorylation of STING and interferon regulatory factor 3 (IRF3), and ultimately, the transcriptional induction of IFN‐I and inflammatory mediators [11, 12, 13]. While the transmembrane domain (TMD) has been the primary focus for inhibitor development, exemplified by covalent inhibitors like H‐151 targeting Cys91 to block palmitoylation and trafficking [14], the CTD is increasingly recognized as the critical structural determinant for the initiating event: functional oligomerization [15, 16]. Despite this insight, the development of inhibitors specifically targeting STING CTD, particularly through covalent mechanisms designed to irreversibly disrupt its oligomerization interface, remains largely unexplored and represents a significant gap in the STING therapeutic landscape [2]. Existing non‐covalent CTD binders [17] and the tentative covalent candidate LB244 [18] highlight the potential of this region but lack the robust mechanistic validation, optimized potency, and clear demonstration of in vivo efficacy needed for therapeutic translation.

We therefore propose a novel therapeutic strategy centered on the covalent targeting of key cysteine residues within STING CTD to disrupt its essential functional oligomerization. Our core hypothesis posits that small molecules capable of forming specific, irreversible bonds with cysteines critical for CTD structural dynamics will effectively prevent the conformational changes required for STING oligomerization and subsequent pathway activation. To rapidly translate this concept into viable therapeutic leads with inherent druggability advantages, we employed a pragmatic “covalent warhead repurposing” approach. This strategy strategically mines libraries of compounds with established cysteine‐targeting capabilities and known pharmacological profiles, leveraging their pre‐existing synthetic accessibility, reduced developmental risk, and often, preliminary safety data [19, 20]. Through systematic in vitro screening against the recombinant STING CTD protein using mass spectrometry‐based covalent modification analysis, we identified P005091, previously characterized as a deubiquitinase USP7 inhibitor [21, 22], as a potent covalent binder targeting multiple CTD cysteines.

We demonstrate that P005091 operates via a unique, non‐classical nucleophilic displacement mechanism, covalently modifying these key cysteines and thereby potently inhibiting STING functional oligomerization, downstream signaling, and I‐IFN production in vitro and in vivo. Crucially, detailed mechanistic dissection revealed an essential dual‐cysteine dependency (Cys292/Cys309) for its inhibitory activity, providing unprecedented insight into the structural basis of STING oligomerization and validating the CTD as a viable target site. Building upon P005091 (renamed NTP1) as a mechanistically validated lead, we rationally optimized NTP1 through a dual‐pronged strategy: enhancing covalent warhead efficiency and improving interactions within the adjacent binding microenvironment. This yielded derivatives NTP14 and NTP16, which showed significantly increased cellular potency against STING signaling. Importantly, NTP14/16 demonstrated superior efficacy in ameliorating inflammation and tissue damage in the DSS‐induced IBD preclinical model.

Our work fundamentally establishes covalent targeting of the STING CTD oligomerization interface as a novel and highly effective therapeutic paradigm. The significant potency gains achieved through rational medicinal chemistry optimization of the repurposed scaffold P005091 underscore the strong druggability of this target site and the substantial translational potential of this approach for treating a broad spectrum of STING‐driven inflammatory and autoimmune diseases.

## 2 Results

### 2.1 Identification of P005091 as a Covalent Warhead Targeting the C‐Terminal Cysteines of STING

**FIGURE 1:**
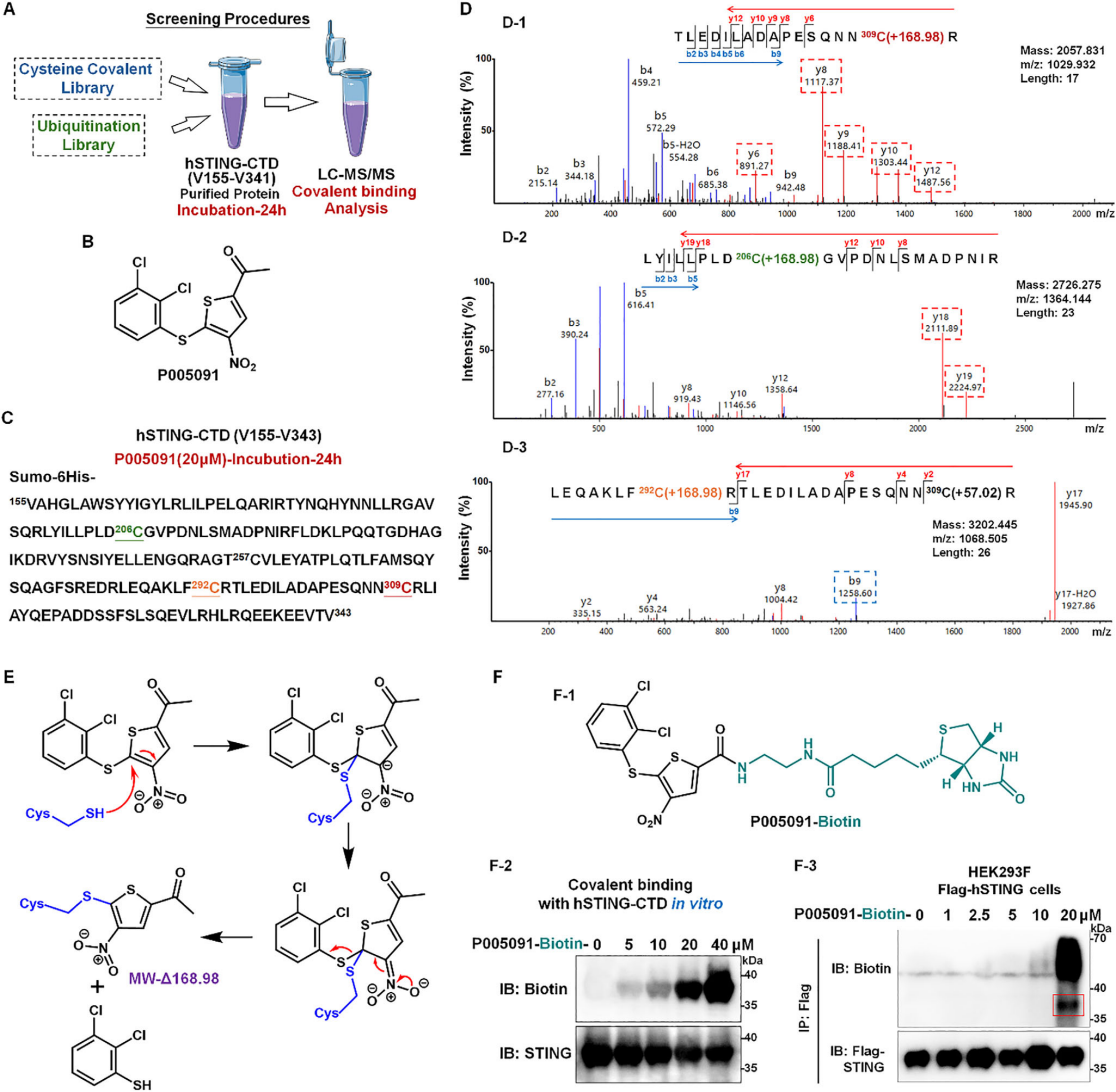
Screening and identification of P005091 as a compound covalently targeting the STING‐CTD. (A) Compound screening workflow: Compounds from a targeted ubiquitination library and a targeted cysteine library (each at 20 µM) were co‐incubated with human N‐Sumo‐6His‐STING‐CTD protein at 4°C for 24 h, followed by covalent analysis via mass spectrometry. (B) Chemical structure of P005091. (C) Amino acid sequence of the N‐Sumo‐6His‐STING (V155‐V343) protein. (D) LC‐MS/MS spectra of STING peptides covalently modified by P005091: (D‐1) Covalent modification at Cys309 residue; (D‐2) Covalent modification at Cys206 residue; (D‐3) Covalent modification at Cys292 residue. (E) Proposed reaction mechanism for the covalent modification of cysteine residues on STING by P005091. (F) Validation of covalent modification of STING‐CTD by P005091 using a biotin tag: (F‐1) Chemical structure of P005091‐Biotin; (F‐2) Binding efficiency of P005091‐Biotin to human STING‐CTD following in vitro incubation at various concentrations (0‐40 µM); (F‐3) Binding efficiency of P005091‐Biotin to Flag‐STING in HEK293F cells following incubation at various concentrations (0‐20 µM). All data were represented from 3 independent biological replicates.

The cytosolic C‐terminal domain (CTD) of the STING protein is increasingly recognized as the key conformational structure for functional oligomerization in subsequent STING activation steps. Thus, we speculate that disulfide bond formation between Cysteine residues within this domain will be an important mechanism for oligomer formation. Based on this analysis, we hypothesized that screening for small molecules capable of covalently binding to the Cysteine residues in the STING‐CTD to prevent intermolecular disulfide bond formation would significantly inhibit STING oligomerization and subsequent activation. This approach will also lay substantial groundwork for developing novel STING inhibitors, as this targeting concept is innovative. Capturing Cysteine primarily involves targeting its sulfhydryl group, enabling the sulfhydryl to undergo a covalent reaction with a small molecule via nucleophilic addition. Therefore, our screening strategy involved clustering known small molecules possessing sulfhydryl‐capturing characteristics, incubating them with the STING‐CTD protein in vitro, and finally identifying those with potential to bind STING‐CTD sulfhydryls through mass spectrometry (MS)‐based covalent modification analysis (Figure 1A and Table S1).

Through screening, we identified the molecule P005091 as having the potential to bind to STING‐CTD Cys206, Cys292, and Cys309 (Figure 1B). We found that P005091 bound to three of the four Cysteine residues in the CTD domain (all except Cys257) (Figures 1C and S1), as evidenced by the detection of peptide masses containing covalently bound P005091 within the confidence interval. This multi‐targeting covalent binding property drew our attention to this molecule. We propose that this multi‐targeting covalent binding capability could provide an important direction for exploring novel inhibitor inhibition modes. Notably, P005091 was originally known as an inhibitor of the deubiquitinating enzyme USP7 [21, 22, 23]. Consequently, we will next focus on studying the inhibition mode of this molecule. MS analysis results showed: 1) Significantly more peptide fragments captured with P005091 bound to Cys309 compared to Cys206 and Cys292; 2) The molecular weight increase measured for the Cysteine sulfhydryl was 168.98 Da (Figure 1D and S2). Further analysis of the covalent binding mode revealed that the sulfhydryl group of Cysteine attacks the position ortho to the nitro group on the thiophene ring of P005091, leading to the “displacement” of the thiophenol structure via an electron transfer effect (Figure 1E and S3A–C). This displacement mechanism is distinct from conventional nucleophilic addition reactions.

**FIGURE 2:**
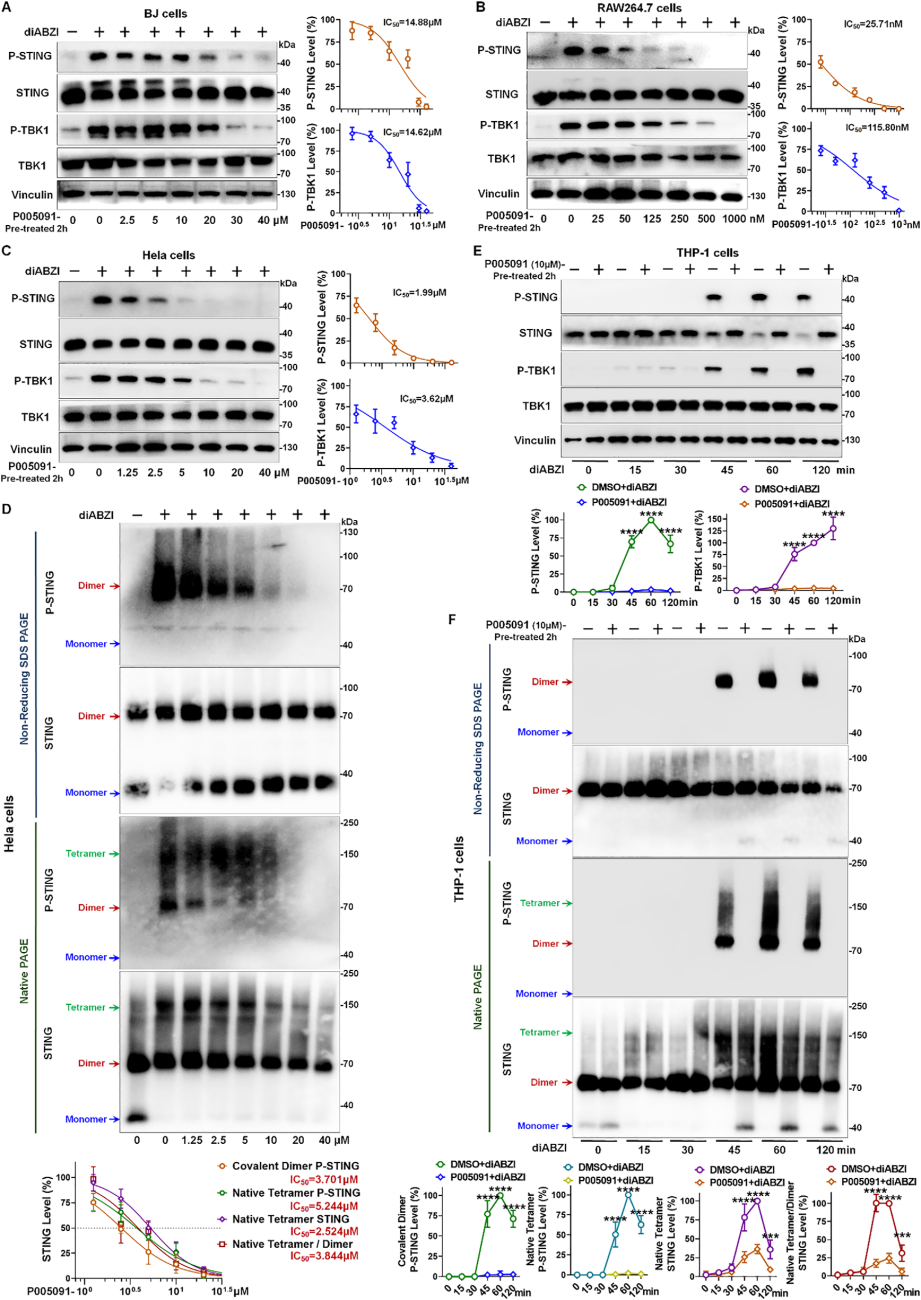
Targeting STING of P005091 suppresses cGAS‐STING pathway activation by abolishing STING oligomerization and trafficking. (A‐C) Dose‐response analysis of P005091 inhibition of STING agonist diABZI (1 µM) or S‐cddA (0.5 µM) ‐induced P‐TBK1 and P‐STING in BJ (A), RAW264.7 (B) and Hela (C) cells. (D) Dose‐response analysis of P005091 inhibition of diABZI (1 µM)‐induced dimerization and oligomerization of STING and p‐STING in HeLa cells. Quantifications of grayscale values data were represented as mean ± S.D. (n = 3), IC_50_ values were determined from 3 independent biological replicates. (E) Kinetic analysis of diABZI (1 µM)‐induced STING activation and its inhibition by P005091 over a 0‐2 h time course in THP‐1 cells. (F) Kinetic analysis of activation levels of STING was assessed by WB, including detection of dimer STING and dimer P‐STING in Non‐reducing SDS PAGE, tetramer STING and tetramer P‐STING in Native PAGE. Quantifications of grayscale values data were represented as mean ± S.D. (n = 3). Statistical significance was determined by two‐way ANOVA (***p < 0.001; ****p < 0.0001).

Subsequently, to further consolidate the covalent targeting capability of P005091 at the molecular level, we conducted verification in two steps: 1) Surface Plasmon Resonance (SPR) and Differential Scanning Fluorimetry (DSF) experiments comprehensively evaluated that P005091 exhibits concentration‐dependent, progressively stronger binding to the STING‐CTD and full‐length STING (Figure S4A–D), the trend observed by DSF is consistent with the covalent binding reported for LB244 (Figure S4E); 2) To evaluate covalent targeting, the P005091 molecule was conjugated with a biotin tag for detection via streptavidin‐based methods (Figure 1F‐1). Results demonstrated that P005091 effectively covalently binds to STING CTD in vitro, exhibiting a pronounced concentration‐dependent effect (Figure 1F‐2). Additionally, time‐dependent binding analysis revealed that P005091 exhibits sustained covalent engagement in vitro incubation with CTD, with binding persistence observed from 2 to 24 h (Figure S5A). To further substantiate the cysteine‐directed covalent binding mechanism, we performed two key validations: 1) after saturating the cysteine residues in the STING CTD with Succinimide, virtually no binding of P005091 to STING was detected (Figure S5B), indicating that cysteine availability is essential for the interaction; 2) co‐incubation with glutathione (GSH) did not alter the binding behavior of P005091 (Figure S6), demonstrating its stability under intracellular reducing conditions. Together, these results reinforce the specificity of P005091 for covalent targeting of cysteine residues within the STING CTD domain. This effect was similarly observed with murine STING (mSTING), which also possesses the four corresponding cysteine residues in its CTD (Figure S7A,B), further supporting the mechanistic conservation across species.

**FIGURE 3:**
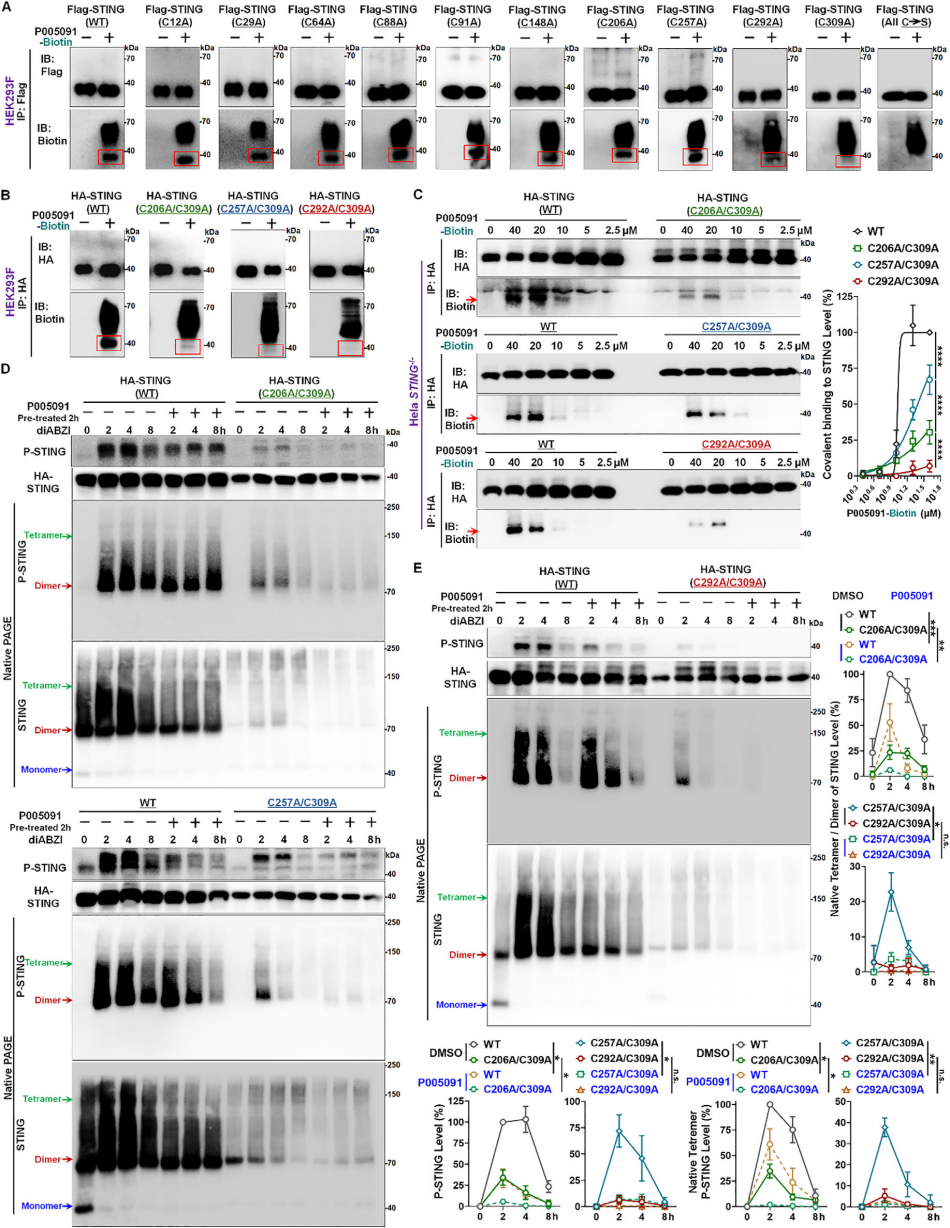
Covalent modification of P005091 at dual sites Cys292/Cys309 blocks STING aggregation and activation. (A) Analysis of P005091‐Biotin (25 µM) covalent binding to STING in lysates from HEK293F cells transfected with Flag‐STING plasmids (WT, C12A, C29A, C64A, C88A, C91A, C148A, C206A, C257A, C292A, C309A and all C to S). (B) Analysis of P005091‐Biotin (25 µM) covalent binding to STING in lysates from HEK293F cells transfected with HA‐STING plasmids (WT, C206A/C309A, C257A/C309A and C292A/C309A). (C) Kinetic analysis of P005091‐Biotin (0‐40 µM) covalent binding to STING in HeLa‐STING KO cells transfected with HA‐STING plasmids (WT, C206A/C309A, C257A/C309A and C292A/C309A). (A‐C) Data was represented from 3 independent biological replicates. (D) Time‐course analysis of diABZI (5 µM) activation and P005091 inhibition in HEK293T cells transfected with HA‐STING (WT, C206A/C309A and C257A/C309A). (E) Time‐course analysis of diABZI (5 µM) activation and P005091 inhibition in HEK293T cells transfected with HA‐STING (WT and C292A/C309A). Quantification of grayscale values for native tetrameric STING and phosphorylated tetrameric p‐STING complexes are presented as mean ± S.D. (n = 3). Statistical significance was determined by two‐way ANOVA (N.S., no significance; *p* > 0.05; *p < 0.05; **p < 0.01; ***p < 0.001; ****p < 0.0001).

Collectively, these findings strengthen the confirmation of the covalent targeting binding mode conclusion for P005091. More importantly, whether this covalent binding mode can occur efficiently within cells—the essential functional environment for a molecule to inhibit STING activation—needed verification. We added the P005091‐Biotin molecule to cells, enriched the STING protein via immunoprecipitation (IP) assay, and then characterized the covalent binding level. Results demonstrated that P005091‐Biotin possesses the capability to covalently capture STING within cells (Figure 1F‐3 and S8A). Furthermore, the binding stability of P005091 to STING was confirmed by time‐course experiments showing sustained interaction over 24 h (Figure S8B). This finding is corroborated by the observation that P005091 binding to mSTING in murine cells reduces its thermal stability (Figure S9A), in a pattern similar to LB244 but even more pronounced (Figure S9B). In summary, we have established that P005091 possesses the ability to covalently target Cysteine residues in the STING‐CTD both in vitro and intracellularly.

As previously established, P005091 is well‐known as a USP7 inhibitor. Therefore, further clarification was required to explain its off‐target covalent engagement with STING. Mass spectrometry analysis confirmed that P005091 also covalently binds to Cysteine 315/334 of USP7 in an identical mechanism (Figures S10 and S11A,B). However, this dual‐targeting property does not compromise its ability to engage STING within cells; in fact, P005091 exhibits a stronger binding preference for STING over USP7 (Figure S12). The functional implications of this dual‐targeting profile on the inhibition of STING signaling will be the subject of further investigation.

### 2.2 P005091 Inhibits STING Functional Oligomerization and the Subsequent Activation Cascade

Having established that P005091 covalently targets Cysteine residues in the STING‐CTD region, we next evaluated its inhibitory efficacy on the STING signaling pathway. We assessed P005091's inhibitory potency (determined by IC_50_ values from dose‐response curves) in human cells—fibroblasts (BJ), monocytes (THP‐1), and tumor cells (HeLa)—under conditions stimulated by STING agonists. Results showed that P005091 inhibited STING self‐activation in these human cells with IC_50_ values ranging from 1 to 15 µM. Its ability to inhibit the activation of TBK1, the core kinase in the STING pathway, was comparable to its inhibition of STING (Figure 2A,C and S13A). Notably, TBK1 activation requires recruitment to STING after its oligomerization, and TBK1 subsequently phosphorylates STING. Furthermore, P005091 exhibited significantly stronger inhibitory potency in murine cells, including macrophages (RAW264.7), fibroblasts (L929), and dendritic cells (DC2.4). In these cells, inhibition of STING self‐activation was maintained at approximately 0.025–1 µM, and inhibition of TBK1 activation ranged from approximately 0.1–1 µM (Figure 2B and S13B,C). All activations used nucleotide‐based STING agonists derived from the cGAMP structure. To better demonstrate P005091's inhibition of activation by endogenous cGAMP, we also delivered cGAMP into cells via transfection. Results consistently showed potent inhibition by P005091 in RAW264.7 cells (Figure S13D). Moreover, the P005091‐Biotin conjugate used in our study also exhibited significant inhibitory activity against STING activation, albeit slightly weaker than that of the unconjugated P005091 due to the presence of the biotin tag (Figure S13E,F). To address potential off‐target effects on USP7, the primary target of P005091, we used XL177A, a potent USP7 inhibitor, as a control. XL177A itself exhibited no inhibitory effect on the STING pathway. However, when combined with P005091, clear inhibition was observed, indicating that USP7 inhibition does not affect STING pathway activation (Figure S14A). Consistent with this conclusion, in STING‐knockout cells, we observed neither activation by STING agonists nor any inhibitory effect of P005091 (Figure S14B). This confirms that the inhibitory effect of P005091 on the STING pathway is directly attributable to its covalent targeting of STING.

We further characterized the impact of P005091's covalent targeting of STING. Given that P005091 inhibited STING self‐activation and TBK1 activation with similar potency, we hypothesized that its inhibitory effect likely occurs prior to STING trafficking. Therefore, we first investigated whether it affects STING oligomerization upon activation. We evaluated this in two ways: 1) Since P005091 targets Cysteine residues in the STING‐CTD, potentially involved in disulfide bond formation for oligomerization, we analyzed STING under non‐reducing SDS‐PAGE conditions (preserving disulfide bonds). Results showed that the activated form of STING (phosphorylated STING) predominantly existed as covalent dimers upon activation, those were observed in both human (BJ, Hela) and murine (RAW264.7) cells (Figure 2D and S15A,B). Treatment with increasing concentrations of P005091 gradually diminished this activated, covalent dimeric form of P‐STING. It is noteworthy that the baseline level of covalent dimerization in resting STING varied across different cell types. Collectively, these data indicate that P005091 inhibits the formation of the activated, covalent dimeric form of STING. 2) We further employed Native PAGE to characterize changes in the native oligomeric state of STING during activation. For P‐STING, we observed a shift to the tetrameric position (considered the functional oligomeric state) upon activation in both human and murine cells. This tetrameric P‐STING band diminished with increasing concentrations of P005091 (Figure 2D and S15A,B). Similarly, the activated tetrameric forms of total STING observed in some cells also disappeared upon P005091 treatment. Taken together, these findings demonstrate that P005091 similarly inhibits both the formation of activated covalent dimers (P‐STING) and the functional oligomerization (tetramerization) of STING under native conditions.

To delineate the temporal dynamics and mechanistic basis of P005091‐mediated inhibition, we first profiled the activation kinetics of the STING pathway. Agonist stimulation induced a rapid activation cascade, with P‐STING becoming detectable at 45 min, peaking at approximately 60 min, and subsequently declining. TBK1 activation followed a congruent kinetic profile. Strikingly, P005091 treatment completely abrogated STING pathway activation at all points examined in THP‐1, Hela and BJ cells (Figure 2E and S16A,B). Since activated STING is known to be rapidly degraded via autophagy, we examined the long‐term effects. We found that while agonist treatment indeed led to rapid STING activation followed by degradation, P005091 alone unexpectedly accelerated STING turnover, potentially due to its covalent action, ultimately leading to a net increase in STING protein (Figure S17A,B). This effect on STING stability likely explains the delayed degradation observed when P005091 is co‐administered with an agonist. Having established the inhibitory effect of P005091 on the activation kinetics, we next focused on the dynamic process of STING oligomerization. We found that: 1) The kinetics of STING activation (P‐STING) detected in reducing‐denaturing gels aligned perfectly with its appearance in both covalent dimers (non‐reducing gels) and tetramers (native PAGE). Importantly, this activation‐dependent oligomerization trend was also consistent with the kinetics of STING's own tetramer formation observed under native conditions. 2) However, the entire oligomerization‐activation process described in 1) was abolished by P005091 treatment (Figure 2F and S18A,B). This clearly demonstrates that P005091 inhibits the functional oligomerization of STING. Furthermore, it is well‐established that activated STING traffics from the ER to the Golgi apparatus, where it recruits and is phosphorylated by TBK1. Using immunofluorescence (IF) to capture trafficking, we found that P005091 indeed significantly inhibited STING trafficking (Figure S19). In summary, P005091 inhibits the STING signaling pathway by covalently targeting Cysteine residues in the STING‐CTD region. This prevents its functional oligomerization, consequently blocking downstream trafficking and the activation cascade.

### 2.3 P005091 Inhibits STING Functional Oligomerization by Covalently Targeting Both Cys292 and Cys309

As previously described, P005091 covalently targets Cys206, Cys292, and Cys309 within the STING CTD domain in vitro. Furthermore, we have established that P005091 exerts its inhibitory function by restricting the formation of functional STING oligomers. Therefore, we sought to further elucidate the precise mechanism by which P005091 mediates inhibition, specifically clarifying its covalent targeting‐inhibition mode. First, we comprehensively characterized the covalent targeting of STING cysteine residues by P005091 in cells to identify potential primary inhibitory sites. Specifically, we individually mutated each of the 10 cysteines on STING to Ala or Ser, transfected these mutants into cells, added the P005091‐Biotin molecule for binding, performed IP to enrich the respective STING proteins, and finally detected binding using streptavidin. The results showed: 1) mutation of all cysteines on the STING protein to Ser abolished P005091 binding to STING. This confirms that P005091 indeed binds via covalent targeting of cysteines. 2) mutation of Cys309 to Ala significantly weakened P005091 binding to STING, with a markedly pronounced reduction. Mutation of Cys292 also showed a weakening trend, but it was less pronounced than that observed with the Cys309 mutation. 3) mutations at other sites, including Cys206, showed binding levels indistinguishable from the wild‐type (WT) protein (Figure 3A). Together, these findings indicate that P005091 function is associated with covalent targeting of Cys292 or Cys309.

Subsequently, we investigated whether single mutation of the Cys292 or Cys309 sites affected STING activation function and further observed whether the inhibitory effect of P005091 was diminished or abolished following single mutation of any cysteine. The results showed that single mutation of several cysteines, including Cys91 and Cys148 (known to be involved in STING oligomerization or trafficking), mutation to Ala did not significantly inhibit STING agonist‐induced activation (Figure S20A). Moreover, the inhibitory effect of P005091 was not diminished or abolished upon single mutation of these cysteines (Figure S20B). Combined with the multi‐site covalent targeting property of P005091, this suggests that P005091 inhibition likely does not result solely from binding to a single cysteine. Building on the result that P005091 binding efficacy was significantly reduced in STING C309A cells, we designed validation experiments using double mutants: C206A/C309A, C257A/C309A, or C292A/C309A. First, we examined the binding efficacy of P005091 to these mutants. Surprisingly, P005091 binding efficacy was significantly reduced for all three double mutants. Notably, binding to the C292A/C309A double mutant was almost completely abolished. Integrating these findings with the single mutant binding results, this indicates that mutation of Cys309 can affect binding to other sites, and the C292A/C309A double mutation likely renders P005091 inhibitory capability ineffective (Figure 3B). To thoroughly validate the binding interactions, we conducted a concentration‐dependent binding assay of P005091 in cells expressing each of the three double mutants. The results were standardized against the WT control run on the same gel to ensure comparability. The data revealed a significant reduction in binding capability for all double mutants compared to WT. Specifically, P005091 binding to the C257A/C309A mutant was substantially stronger than its binding to the C206A/C309A mutant, while its binding to the C292A/C309A double mutant was the most severely impaired (Figure 3C). This hierarchy of binding affinity underscores C309 as a primary binding site and suggests that C292 and C309 together constitute a preferential binding pocket for P005091.

We then characterized the state of STING functional oligomerization to determine whether P005091's inhibitory efficacy is achieved through dual targeting of Cys292 and Cys309. To determine if P005091 inhibits STING oligomerization by dual targeting Cys292 and Cys309, we assessed this process in 293T cells reconstituted with WT STING or the C292A/C309A, C206A/C309A, and C257A/C309A double mutants, thereby excluding background effects. The strategy involved STING agonist stimulation followed by dynamic observation using Native‐PAGE. The results showed: 1) In cells expressing the C206A/C309A or C257A/C309A double mutants, agonist‐induced STING activation—as measured by the levels of P‐STING and P‐TBK1‐was significantly attenuated compared to wild‐type STING. This was accompanied by a marked reduction in the formation of STING tetramers relative to dimer levels at the 2‐h activation peak. The overall kinetic profile of activation was also substantially weaker in these mutants. Notably, despite this compromised activation, P005091 still effectively inhibited both oligomerization and phosphorylation in both the C206A/C309A and C257A/C309A mutant backgrounds (Figure 3D and S21). 2) In stark contrast, the C292A/C309A double mutant exhibited a much more severe phenotype. Both the degree of STING activation and the level of tetramer formation observed 2 h post‐stimulation were drastically lower than those in the C206A/C309A and C257A/C309A mutant groups. Furthermore, P‐STING, which typically migrates at the tetramer position, was nearly undetectable in this mutant (Figure 3E and S21). This indicates that the simultaneous mutation of Cys292 and Cys309 potently disrupts STING oligomerization and subsequent activation. Crucially, in the C292A/C309A mutant background, the inhibitory effect of P005091 was completely abolished. To extend these findings, we expressed murine WT STING and its corresponding C291A/C308A double mutant (homologous to human C292A/C309A) in cells. The same patterns were recapitulated: the double mutation strongly suppressed oligomerization and activation, and P005091 lost its inhibitory effect (Figure S22).

Collectively, these results demonstrate that the inhibitory efficacy of P005091 is dependent on its covalent targeting of Cys292 and Cys309, with the C292A/C309A double mutation completely abrogating its function.

### 2.4 P005091 Inhibits STING‐Activated Type I IFN Responses In Vitro and In Vivo

**FIGURE 4:**
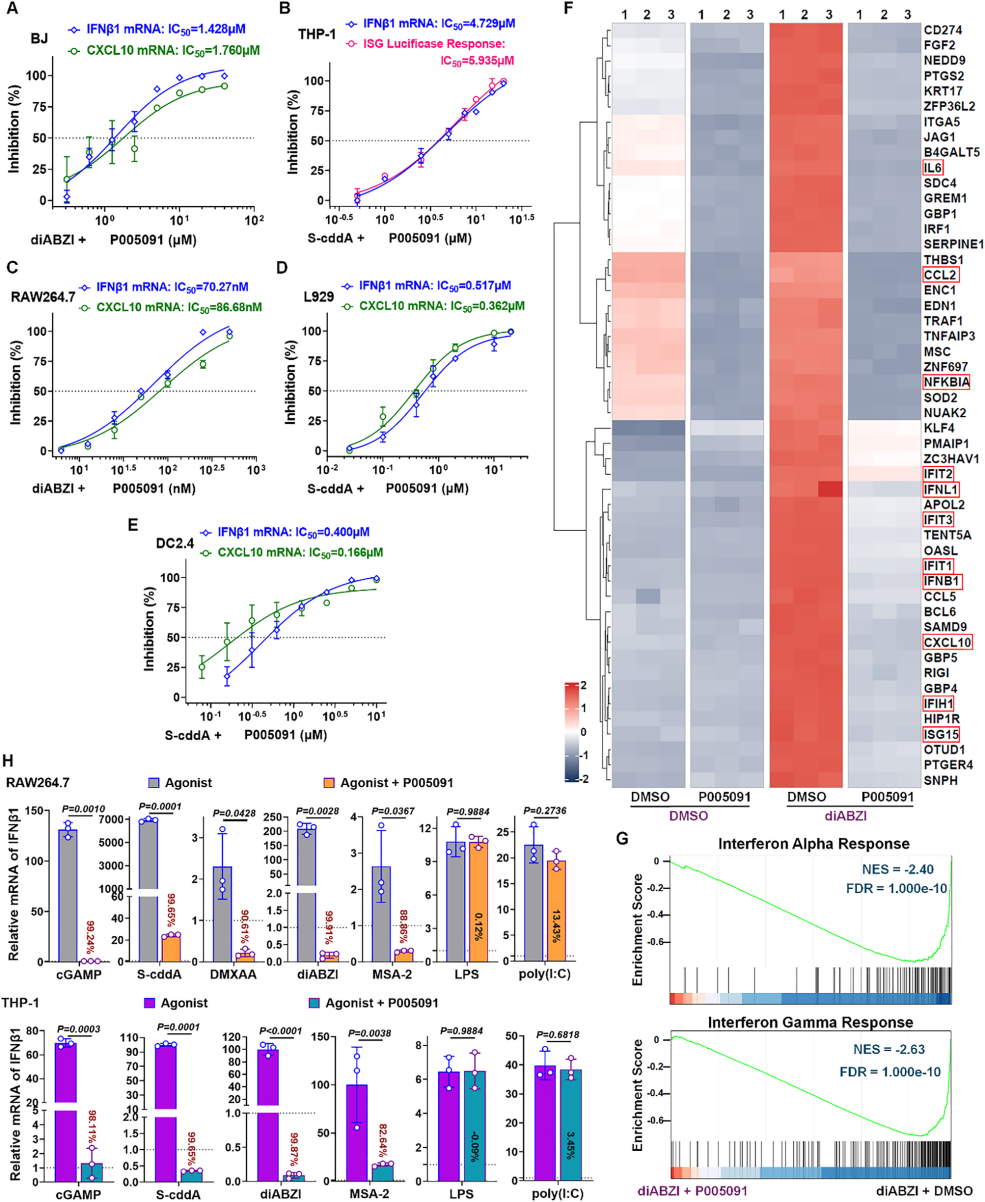
Inhibitory efficacy of P005091 on the STING signaling pathway. (A) Dose‐response analysis of P005091 inhibition of diABZI (1 µM)‐induced IFN‐β1 and CXCL10 responses in BJ cells. (B) Dose‐response analysis of P005091 inhibition of S‐cddA (0.5 µM)‐induced IFN‐β1 response and ISG sensing levels in THP‐1 cells. (C) Dose‐response analysis of P005091 inhibition of diABZI (10 µM)‐induced IFN‐β1 and CXCL10 responses in RAW264.7 cells. (D) Dose‐response analysis of P005091 inhibition of S‐cddA (0.5 µM)‐induced IFN‐β1 and CXCL10 responses in L929 cells. (E) Dose‐response analysis of P005091 inhibition of S‐cddA (5 µM)‐induced IFN‐β1 and CXCL10 responses in DC2.4 cells. (A–E) IC_50_ values were determined from 3 independent biological replicates. (F) The four‐cluster heatmap of total mRNA from BJ cells pre‐incubated with P005091 (20 µM) and then co‐treated with diABZI (1 µM), followed by RNA sequencing (n=3/group). (G) Gene set enrichment analysis (GSEA) of total mRNA from BJ cells pre‐incubated with P005091 (20 µM) and then co‐treated with diABZI (1 µM), followed by RNA sequencing (n=3/group). (H) Top: Dose‐response analysis of IFN‐β1 inhibition by P005091 (2 µM pre‐incubation) in RAW264.7 cells co‐treated for 2 h with STING agonists cGAMP (1 µM), S‐cddA (0.5 µM), DMXAA (20 µM), diABZI (10 µM), MSA‐2 (40 µM), for 12 h with LPS (10 µg/mL), or for 3 h with Poly I:C (5 µg/mL). Below: Dose‐response analysis of IFN‐β1 inhibition by P005091 (10 µM pre‐incubation) in THP‐1 cells co‐treated for 2 h with STING agonists cGAMP (25 µM), S‐cddA (0.1 µM), diABZI (10 µM), MSA‐2 (40 µM), for 12 h with LPS (10 µg/mL), or for 3 h with Poly I:C (5 µg/mL). Data were represented as mean ± S.D. (n = 3). Statistical significance was determined by two‐way ANOVA (N.S., no significance; *p* > 0.05; *p < 0.05; **p < 0.01; ***p < 0.001; ****p < 0.0001).

P005091 demonstrates definitive inhibitory efficacy against STING‐activated type I IFN inflammatory responses both in vitro and in vivo. STING is the central executor of the type I interferon response (innate immune response) triggered by dsDNA. The inhibitory effect on the STING pathway resulting from P005091's covalent targeting of STING, and consequently the degree of suppression of the type I IFN response, required immediate and thorough evaluation. This determination is crucial for assessing P005091's potential as an anti‐inflammatory drug candidate. The most direct characterization of the type I IFN response involves detecting type I IFN expression levels, complemented by assessing the response level or expression of interferon‐stimulated genes (ISGs). For inhibition rate characterization, gradient dose‐response results were used for fitting. The results showed that in human BJ and THP‐1 cells, P005091 inhibited type I IFN expression with IC_50_ values ranging approximately from 1 to 5 µM. The inhibition of the key downstream ISG chemokine CXCL10 and the inhibition of ISG transcriptional response (assessed via reporter gene assay) also yielded IC_50_ values largely within the range of 1 to 6 µM (Figure 4A,B and S23A,B). In murine cells, the inhibitory effect was significantly stronger: P005091 inhibited type I IFN expression with IC_50_ values ranging from 0.07 to 0.5 µM, and inhibited CXCL10 expression with IC_50_ values ranging from 0.08 to 0.4 µM (Figure 4C–E and S23C–E). Subsequently, the inhibitory performance of P005091 was further examined by varying the concentration of STING agonists. The results revealed that even strong activation effects induced by high concentrations of STING agonists, including cGAMP, were completely inhibited within the effective concentration range of P005091 (Figure S24A,B). Moreover, even under conditions ensuring strong type I IFN activation (by adding pro‐activating targeting factors) [24, 25], P005091 consistently exhibited high inhibitory potency (Figure S24C). Additionally, the USP7 inhibitor XL177A was employed to demonstrate that the observed effects of P005091 were not due to off‐target inhibition of USP7 (Figure S24D). Furthermore, in STING‐ KO cells, we observed neither agonist‐induced activation nor any inhibitory effect of P005091, confirming that both processes are strictly STING‐dependent (Figure S24E). Characterization also revealed that P005091 has only weak proliferative inhibitory activity on cells over extended periods, supporting its safety profile (Figure S24F).

Transcriptomic analysis (RNA‐seq) of total intracellular mRNA levels further showed that: 1) STING agonists induced a clear transcriptional activation response downstream of type I IFN, which also included activation of the IRF3‐parallel NF‐κB signaling pathway; 2) This activation process was blocked by the addition of P005091, reducing the levels to baseline transcriptional levels of the relevant pathways (Figure 4F,G); 3) Importantly, P005091 alone did not affect the transcription of IFN‐I, IFN‐II, or other unrelated immune pathways, underscoring the specificity of its inhibitory action (Figure S25). This indicates that P005091 exerts a significant inhibitory effect on type I IFN signaling. Furthermore, to robustly confirm that the inhibitory effect of P005091 specifically targets the STING‐activated type I IFN pathway (i.e., specificity validation), multiple STING agonists were used. P005091 exhibited strong inhibitory activity against the type I IFN responses induced by these agonists in both human and murine cells. However, it showed no inhibitory efficacy against responses activated non‐specifically by LPS or poly (I:C) (Figure 4H), thereby fully validating its specificity for inhibiting STING‐activated type I IFN responses.

**FIGURE 5:**
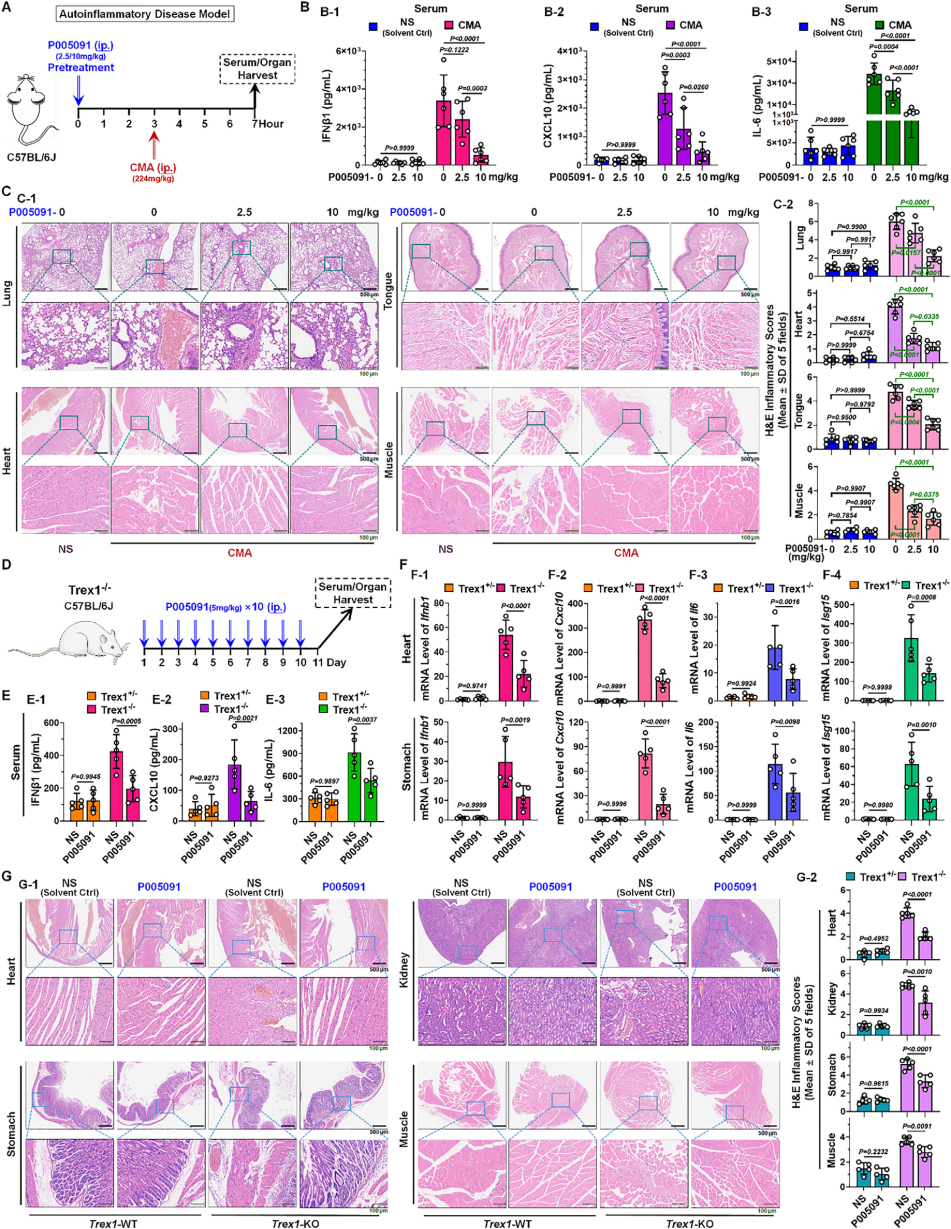
P005091 alleviates CMA‐induced acute inflammation and Trex1 deficiency‐mediated autoimmune disease. (A) Schematic diagram of the experimental model for CMA‐induced acute inflammation disease in mice. (B) Levels of inflammatory cytokines in mice serum: (B‐1) IFN‐β1; (B‐2) CXCL10; (B‐3) IL‐6. (C) H&E staining analysis of mice lungs, tongues, hearts, and muscle tissues: (C‐1) Representative H&E staining images and corresponding magnified views (insets); (C‐2) H&E inflammation scores. (D) Schematic diagram of the experimental model for autoimmune disease in Trex1^−/−^ mice. (E) Levels of inflammatory markers in mice serum: (E‐1) IFN‐β1; (E‐2) CXCL10; (E‐3) IL‐6. (F) mRNA expression levels of inflammatory markers in heart and stomach tissues from Trex1‐WT and Trex1‐KO mice: (F‐1) IFN‐β1; (F‐2) CXCL10; (F‐3) IL‐6; (F‐4) ISG15. (G) H&E staining analysis of heart, kidney, stomach, and muscle tissues from Trex1‐WT and Trex1‐KO mice: (G‐1) Representative H&E staining images and corresponding magnified views (insets); (G‐2) H&E inflammation scores. Data were represented as mean ± S.D. (n ≥ 5). Statistical significance was determined by two‐way ANOVA (N.S., no significance; *p* > 0.05; *p < 0.05; **p < 0.01; ***p < 0.001; ****p < 0.0001).

We then proceeded to evaluate P005091 in vivo. First, an acute inflammation model in mice was established using a very high concentration of the STING agonist CMA. P005091 intervention was administered via pre‐injection (Figure 5A). Analysis of collected serum samples showed that a single dose of P005091 effectively suppressed the surge in serum type I IFN and its downstream factors caused by CMA, exhibiting a clear dose‐response relationship. Notably, at a dose of 10 mg/kg, the inhibitory effect exceeded 80% (Figure 5B). Furthermore, in assessing the degree of inflammatory damage in various organs, P005091 demonstrated a significant dose‐response relationship in alleviating damage to the lungs, heart, tongue, smooth muscle, liver, and kidneys. At the 10 mg/kg dose, the alleviation rate exceeded 50% in all cases (Figure 5C and S26 and S27). This indicates that P005091 exerts significant anti‐acute inflammatory efficacy in vivo. Further evaluation was conducted in a model of intrinsic STING activation in vivo. The principle relies on the global knockout of the TREX1 exonuclease, which impairs the dsDNA degradation system, leading to the release and accumulation of dsDNA. This results in persistent activation of the STING pathway [14]. 4‐week‐old TREX1‐KO mice were treated with consecutive injections for 10 days (Figure 5D), followed by characterization of serum markers and organ damage levels; WT mice served as controls. The results showed: 1) Continuous treatment with P005091 significantly reduced the levels of type I IFN and its downstream factors in the serum of TREX1‐KO mice. Serum marker levels in WT mice were significantly lower than in KO mice, and P005091 had minimal effect on WT mice (Figure 5E); 2) Assessment of type I IFN and its downstream factor expression levels (mRNA) in heart and stomach tissue revealed that P005091 markedly inhibited the surge in expression caused by TREX1 knockout, with efficacy generally exceeding 50% (Figure 5F); 3) In evaluating organ inflammatory damage, P005091 demonstrated highly prominent alleviating effects on damage to the heart, kidneys, stomach, smooth muscle, liver, and lungs (Figure 5G and S28).

Collectively, these results demonstrate that the inactivation of the STING pathway caused by P005091's covalent targeting and inhibition of STING leads to a clear inhibitory effect on type I IFN‐mediated inflammatory responses.

### 2.5 P005091 Exhibits Significant Efficacy in Treating Inflammatory Bowel Disease

**FIGURE 6:**
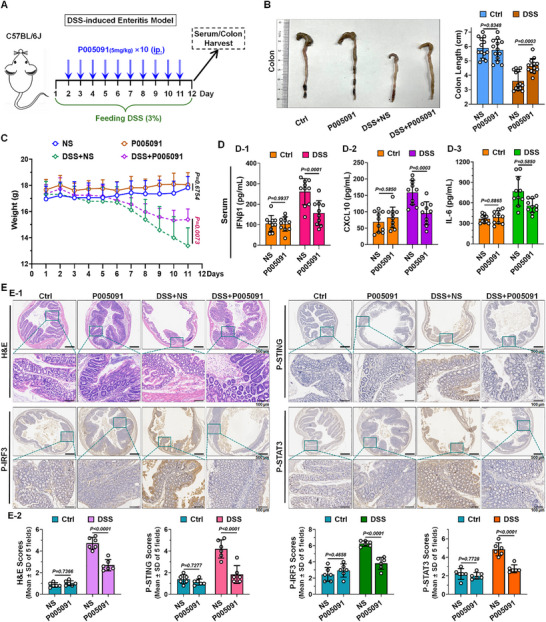
P005091 alleviates DSS‐induced colitis in mice. (A) Schematic diagram of the experimental model for DSS‐induced colitis in mice. (B) Representative photographs and length measurements of colons harvested from mice at the end of the experiment. (C) Daily recording of mouse body weight. (D) Levels of inflammatory markers in serum from Ctrl and DSS‐treated mice: (D‐1) IFN‐β1; (D‐2) CXCL10; (D‐3) IL‐6. (E) H&E staining and IHC staining analysis for P‐STING, P‐IRF3 and P‐STAT3 in colon tissues from Ctrl and DSS‐treated mice: (E‐1) Representative H&E and IHC staining images; (E‐2) Corresponding H&E and IHC scores. Data were represented as mean ± S.D. (n ≥ 6). Statistical significance was determined by two‐way ANOVA (N.S., no significance; *p* > 0.05; **p < 0.01; ***p < 0.001; ****p < 0.0001).

The pathogenesis of inflammatory bowel disease (IBD) is closely associated with the activation of innate immunity in the gut microenvironment. Damaged intestinal epithelial cells release damage‐associated molecular patterns (DAMPs), including dsDNA and mtDNA, which activate the type I IFNs pathway predominantly mediated by cGAS‐STING [9]. The dextran sulfate sodium (DSS)‐induced IBD model is a classic and widely used experimental animal model for studying the pathogenesis of such diseases and evaluating drug efficacy [26]. Therefore, we also conducted an in vivo DSS‐induced experiment to assess the therapeutic effect of P005091. We employed a prolonged continuous DSS induction model and administered P005091 continuously for 10 days starting 1 day after induction initiation (Figure 6A). The results showed that: 1) DSS induction caused significant intestinal toxicity, manifested as a substantial reduction in colon length and sustained weight loss in mice starting from day 6 of induction (Figure 6B,C). 2) Intervention with P005091 significantly mitigated the trend of colon shortening and slowed the decline in mouse body weight, demonstrating a clear therapeutic effect (Figure 6B,C). 3) Analysis of serum indicators revealed that levels of I‐IFNs and their downstream factors released into the bloodstream due to DSS‐induced intestinal inflammation were considerably high, typically increasing several‐fold. However, P005091 intervention markedly reduced the levels of these inflammatory factors in serum (Figure 6D). 4) Histopathological evaluation by H&E staining indicated that P005091 significantly ameliorated DSS‐induced colon damage and effectively reduced inflammatory cell infiltration (Figure 6E). 5) Assessment of STING pathway activation in the intestine revealed that P005091 significantly suppressed the DSS‐induced activation of the STING pathway (as indicated by P‐STING and P‐IRF3) and the associated downstream inflammatory marker (P‐STAT3) (Figure 6E).

To further validate in vivo that the anti‐inflammatory efficacy of P005091 is mediated through covalent targeting of STING rather than via off‐target inhibition of USP7, we conducted experiments using three distinct murine models of inflammatory bowel disease (IBD). 1) Evaluation of a distinct USP7 inhibitor: We assessed whether XL177A, a structurally unrelated USP7 inhibitor, could ameliorate colitis. Results showed that XL177A failed to improve DSS‐induced intestinal inflammation (as assessed by colon length) or systemic toxicity (as indicated by body weight change) (Figure S29), suggesting that USP7 inhibition alone does not replicate the therapeutic effect of P005091. 2) Functional interaction with a canonical STING inhibitor: We next performed a combination study with H‐151, a known STING inhibitor. H‐151 monotherapy significantly alleviated colitis. Notably, no additive or antagonistic effects were observed when H‐151 was co‐administered with P005091 (Figure S30). This functional non‐interference indicates that both compounds likely share the same molecular target in vivo. 3) Genetic validation in STING‐KO mice: To provide direct genetic evidence, we subjected STING‐KO mice to the DSS model. While DSS still induced body weight loss in STING‐KO mice, P005091 treatment no longer conferred any therapeutic benefit, as measured by colon length or body weight recovery (Figure S31). Collectively, these in vivo results robustly demonstrate that the anti‐inflammatory effect of P005091 is specifically dependent on targeting STING. Additionally, we evaluated the in vivo safety of P005091. A 15‐day consecutive administration regimen revealed no significant adverse effects on body weight or organ toxicity in either WT or STING‐KO mice (Figure S32), indicating that P005091 is well‐tolerated and exhibits a favorable safety profile.

In summary, these findings indicate that the STING‐inhibitory effect of P005091 has a significant positive impact on the treatment of DSS‐induced IBD. P005091 can serve as a lead compound for exploring treatments for diseases including Crohn's disease and ulcerative colitis, providing strong preclinical evidence for the development of novel IBD therapeutics.

### 2.6 Initial Optimization of the P005091 Molecular Structure Shows Enhanced Activity

**FIGURE 7:**
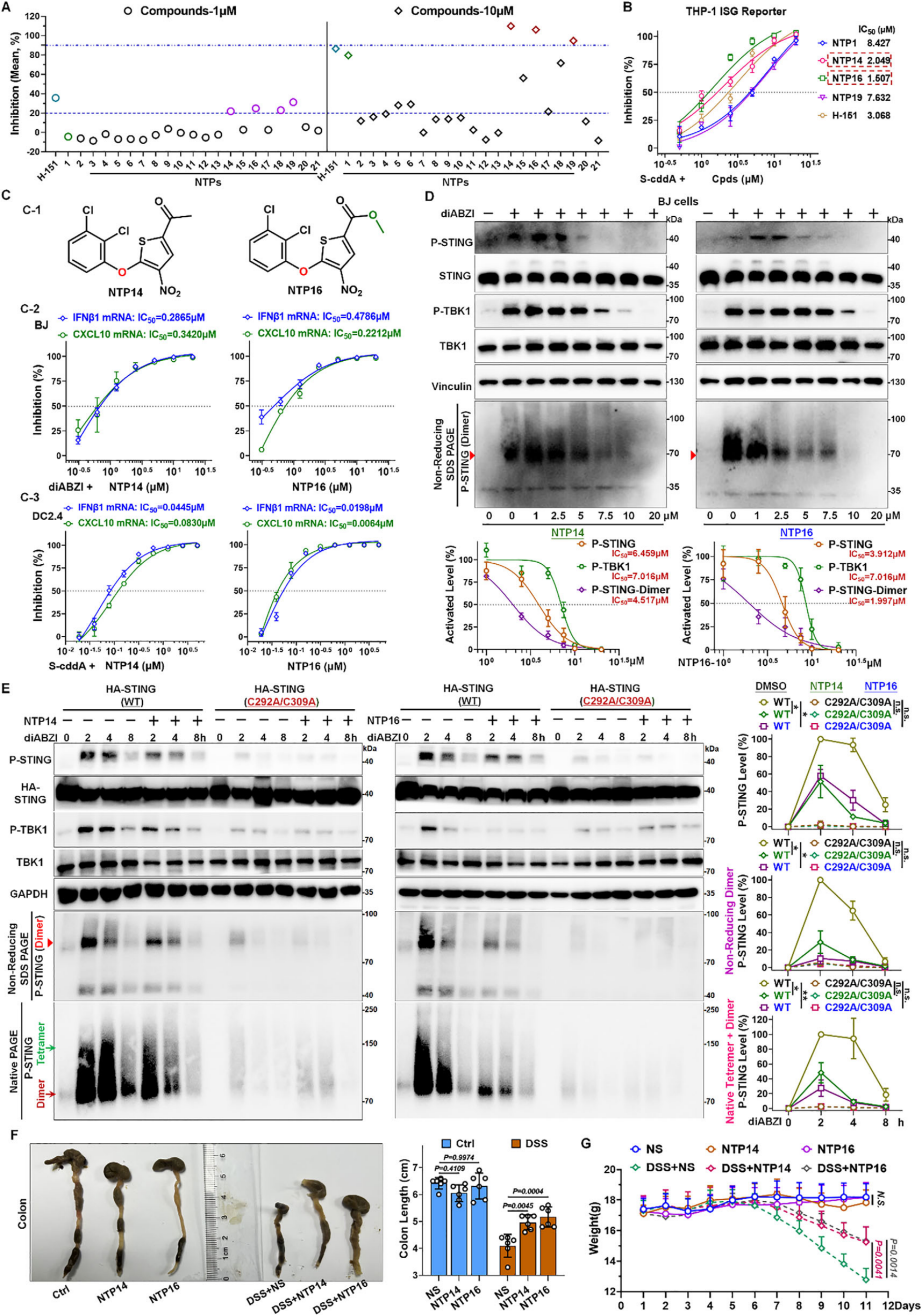
Structural analogs NTP14 and NTP16 exhibit enhanced STING inhibitory activity in vitro and in vivo. (A) Inhibitory activity screening of P005091 structural analogs in THP1‐Lucia ISG cells. (B) Dose‐response analysis and IC_50_ determination for prioritized compounds from (A). (A‐B) Data was represented from 3 independent biological replicates. (C) Dose‐dependent inhibition of diABZI (1 µM) and S‐cddA (0.5 µM) induced IFN‐β1 and CXCL10 responses by NTP14 and NTP16: (C‐1) Chemical structures of NTP14 and NTP16; (C‐2) Dose‐response curves and IC_50_ values in BJ cells; (C‐3) Dose‐response curves and IC_50_ values in DC2.4 cells. IC_50_ values were determined from 3 independent biological replicates. (D) Dose‐dependent inhibition of diABZI (1 µM)‐induced STING and TBK1 phosphorylation by NTP14 and NTP16 in BJ cells. Top: Representative immunoblots under reducing and non‐reducing SDS‐PAGE conditions; Bottom: Densitometric quantification of p‐TBK1, p‐STING, and p‐STING‐dimer with IC_50_ calculations. Quantifications of grayscale values data were represented as mean ± S.D. (n = 3), IC_50_ values were determined from 3 independent biological replicates. (E) Time‐course analysis of STING activation by diABZI (5 µM) and inhibition by NTP14 and NTP16 in HEK293T cells transfected with HA‐STING (WT and C292A/C309A). Quantification of grayscale values for native tetrameric STING and phosphorylated tetrameric p‐STING complexes are presented as mean ± S.D. (n = 3). (F) Colon photographs and length measurements at experimental endpoint in the DSS‐induced acute colitis model. (G) Daily body weight recording (n = 6). Data were represented as mean ± S.D. (n ≥ 3). Statistical significance was determined by two‐way ANOVA (N.S., no significance; *p* > 0.05; *p < 0.05; **p < 0.01; ***p < 0.001).

As previously described, P005091 was identified through covalent targeting screening against the cysteine in the CTD of STING, employing a “drug repurposing” strategy to discover compounds with known structures and targets. Consequently, guided by drug‐likeness considerations, structural optimization of P005091 as a lead molecule, with a primary focus on enhancing its activity, became the critical next step. Based on structural analysis and initial modification efforts, and considering its covalent targeting feature (renamed NTP1, reflecting its Nitro group‐Thiophene‐Phenyl group core), modifications were primarily directed toward two aspects: 1) Diversity modification of substituents on the benzene ring of the benzenethiol leaving group post‐covalent binding, yielding molecules NTP2‐13. Additionally, the bridging sulfur (S) atom was replaced with the more electronegative oxygen (O) atom, resulting in molecule NTP14. 2) Diversity modification of the ortho‐substituent on the thiophene ring of the covalent binding motif, yielding molecules NTP15‐21, wherein NTP16 also features an O‐bridge (Figure S33). Subsequently, the inhibitory activities of these 20 new molecules, alongside controls NTP1 and the known inhibitor H‐151, were evaluated using an ISG reporter assay in THP1 cells. The results showed that: 1) At a low concentration (1 µM), four molecules (NTP14, NTP16, NTP18, NTP19), including the control H‐151, exhibited inhibition rates exceeding 20%. Notably, this surpassed the inhibition effect of NTP1 (Figure 7A); 2) At a high concentration (10 µM), NTP14 and NTP16 achieved near‐complete inhibition, with inhibition rates higher than those of H151, NTP1, and the other molecules (Figure 7A). 3) Further dose‐response evaluation confirmed a clear enhancement in activity for NTP14 and NTP16 compared to both H‐151 and NTP1 (Figure 7B and S34). Therefore, the inhibitory potency of these two molecules was selected for comprehensive assessment.

The structural changes in NTP14 (S bridge to O) and NTP16 (S bridge to O, and acetyl group ortho to the thiophene replaced with a methoxycarbonyl group in NTP16) suggest that the observed activity enhancement may arise from the stronger covalent targeting ability of the O‐bridged molecules, and the methoxycarbonyl modification in NTP16 is beneficial for activity enhancement (Figure 7C‐1). Subsequently, the inhibitory potency on the I‐IFN response was evaluated. Compared to NTP1, NTP14 showed a five to tenfold increase in inhibitory activity in both human and murine cells. NTP16 exhibited comparable potency to NTP14 in human cells but demonstrated over a 20‐fold increase in murine cells (Figure 7C‐2/3 and S35A–D).

We then conducted an in‐depth mechanistic analysis:  Initial characterization by SPR revealed that NTP14 and NTP16 bind to STING with markedly higher affinity than NTP1 (Figure S36A,B). This enhanced binding capability provided a molecular basis for their superior functional performance: both compounds, particularly NTP16 (which showed an approximately fivefold increase in potency), more effectively inhibited the formation of functional P‐STING tetramers (Figure 7D). This explains the superior inhibitory activity of NTP14 and NTP16 on the STING pathway compared to NTP1 and validates the preliminary success of our dual‐modification strategy. We next investigated whether NTP14 and NTP16 share the same binding mode with STING as the parent compound P005091 (NTP1). Using a cysteine reactivity assay, we found that both new compounds engaged the thiol groups of STING cysteine residues in a manner identical to P005091, adhering to the same reaction principle involving “displacement” of the thiophenol structure (Figure S37A,B). To further corroborate this, we assessed their activity in cells expressing the C292A/C309A STING double mutant. Notably, the ability of both NTP14 and NTP16 to inhibit STING pathway activation and functional oligomerization was virtually abolished in this mutant background (Figure 7E and S38). These results conclusively demonstrate that the structural optimizations in NTP14 and NTP16 did not alter their mechanism of covalent engagement with STING, confirming mechanistic consistency with the parent scaffold.

In the validation of pharmacodynamics for IBD treatment, NTP14 and NTP16 demonstrated potent effects in mitigating colon shortening and body weight loss in mice. Notably, both molecules showed an advantage over NTP1 in slowing weight loss, and their efficacy levels were nearly equivalent (Figure 7F,G). Further assessment of intestinal inflammatory damage revealed that NTP14 and NTP16 significantly ameliorated tissue damage and suppressed inflammation (Figure S39A). Concurrently, serum levels of inflammatory cytokines were markedly reduced following NTP14/NTP16 intervention (Figure S39B). Collectively, the results confirm the validity of the modification strategy aimed at enhancing pharmacodynamics. These also demonstrates significant potential for further structural optimization based on the NTP1 (P005091) scaffold, providing more diverse candidate molecules for the treatment of IBD and other inflammatory diseases.

## 3 Discussion

Innate immunity constitutes a fundamental defense mechanism against pathogenic invasion and internal stress signals. Dysregulated innate immune activation, particularly through pattern recognition receptors like STING, is increasingly implicated in the pathogenesis of chronic inflammatory and autoimmune diseases [27, 28]. The cGAS‐STING pathway acts as a critical sensor for cytosolic dsDNA, a potent DAMP, triggering robust type I IFN and NF‐κB inflammatory responses [29]. While essential for host defense, persistent or inappropriate STING activation drives pathological inflammation in conditions such as lupus, Aicardi‐Goutières syndrome, and notably, IBD [30, 31]. Current IBD therapies primarily target adaptive immune components (e.g., anti‐TNFα antibodies) or downstream inflammatory mediators, leaving a significant unmet need for agents that modulate upstream innate immune drivers like STING [9]. Our study demonstrates the potent therapeutic efficacy of the covalent STING inhibitor P005091 and its optimized derivatives (NTP14/16) in a DSS‐induced IBD model. By specifically targeting and inhibiting STING functional oligomerization, P005091 effectively suppressed the STING‐dependent type I IFN cascade, significantly reducing serum inflammatory cytokines (e.g., IFNβ, CXCL10) and ameliorating multi‐organ damage (with >50% alleviation rates at 10 mg/kg). Notably, although P005091 was originally reported as a USP7 inhibitor, our findings reveal its potent off‐target effect through direct STING engagement. To confirm that the observed anti‐IBD efficacy is not mediated by USP7 inhibition‐particularly since USP family proteins, including USP7, have been implicated in IBD pathogenesis via inflammasome or NF‐κB pathways [32]‐we evaluated an alternative USP7 inhibitor, XL177A, in vivo. The lack of significant intestinal protection or anti‐inflammatory effects with XL177A strongly supports that P005091's therapeutic benefits are attributable to its direct targeting of STING rather than USP7. These findings underscore the critical role of STING hyperactivation in sterile inflammation and establish covalent STING inhibitors as promising candidates for developing novel, mechanism‐based anti‐inflammatory therapeutics for IBD and potentially other STING‐driven pathologies like systemic autoimmunity, neuroinflammation, and fibrosis.

Current strategies for STING inhibition largely focus on the transmembrane domain (TMD), exemplified by covalent inhibitors like H‐151, which targets Cys91 to prevent palmitoylation and subsequent STING activation and trafficking [14]. Other inhibitors, such as GHN105 [33], BPK‐25 [34], NO_2_‐FAs [35] (Cys91), LicoD [36], and 4‐octyl itaconate [37] (Cys148), also act within the TMD. While non‐covalent inhibitors targeting the cytosolic CTD (e.g., SN‐011 [17], Ginkgetin [38], PTS [39]) show promise, covalent targeting of the CTD remained largely unexplored, with only LB244 tentatively linked to Cys292 [18]. Our study breaks new ground by establishing a novel inhibition paradigm: covalent disruption of STING functional oligomerization via dual targeting of Cys292 and Cys309 within the CTD. P005091 achieves this through a unique, non‐classical nucleophilic addition mechanism involving electron transfer and thiophenol displacement. Its mechanism presents three key innovations: 1) Targeting Mechanism Innovation: We provide the first direct evidence that a disulfide network involving Cys292 and Cys309 is a crucial structural basis for STING CTD oligomerization. Covalent modification of these residues by P005091 prevents the essential conformational transition from monomeric/resting states to the functional oligomeric state, as rigorously validated by complementary techniques (non‐reducing SDS‐PAGE showing loss of activated dimers and Native PAGE showing loss of functional tetramers). 2) Site‐Selectivity Breakthrough: Unlike single‐site covalent inhibitors targeting the TMD (e.g., Cys91), P005091 exhibits a unique dependence on dual covalent engagement of Cys292 and Cys309. Single mutations (C292A or C309A) significantly reduced binding, while the C292A/C309A double mutation completely abrogated both P005091 binding and its inhibitory effect on agonist‐induced oligomerization and activation. This dual‐site requirement overcomes the potential for functional compensation often seen with single cysteine mutations. 3) Inhibitory Efficiency Advantage: The optimized derivative NTP16, benefiting from structural modifications enhancing its covalent reaction and cellular activity, demonstrated a fivefold increase in potency for inhibiting functional P‐STING tetramer formation compared to the parent P005091 (NTP1). This potency surpasses many reported non‐covalent CTD inhibitors (e.g., SN‐011) and highlights the potential efficiency gain achievable with covalent targeting. This discovery provides a novel paradigm for tackling the challenging dynamic protein‐protein interaction (PPI) interface involved in STING oligomerization, opening new avenues for covalent drug design targeting PPIs.

Our investigation into P005091's mechanism yielded unexpected insights into the heterogeneity of STING basal states. We observed cell‐type‐specific differences in the presence of covalent STING dimers under resting conditions: prominent in BJ fibroblasts but minimal in HeLa or RAW264.7 cells. This suggests that BJ cells may harbor STING in a non‐activating, pre‐formed covalent dimeric conformation, poised for conversion into an active oligomeric state upon ligand binding. In contrast, HeLa and RAW264.7 cells appear to rely more heavily on agonist‐induced dimerization/oligomerization initiated from monomeric or loosely associated states. P005091, by covalently locking key cysteines (C292/C309) involved in both basal and activation‐induced oligomerization interfaces, proved to be an invaluable tool molecule for dissecting these distinct pathways. Its ability to selectively inhibit the transition to the functional tetramer, regardless of the starting oligomeric state, underscores its utility beyond therapeutic potential as a precise probe for mechanistic studies of STING activation dynamics [40, 41]. This mechanistic insight further justifies our approach to optimize its binding and inhibitory properties. Additionally, although the cysteine residues targeted by P005091 (Cys292/Cys309 in human STING; corresponding to Cys291/Cys308 in murine STING) are conserved, the surrounding amino acid sequences exhibit subtle differences [42, 43]. Our analysis of the protein structures suggests that the binding pocket in murine STING might be slightly more accessible or possess a local chemical environment that favors the covalent binding reaction of P005091. This increased binding efficiency likely underpins the approximately tenfold higher potency we observed in murine cells compared to human cells. While we were unable to obtain a high‐resolution structure of the murine STING CTD in complex with P005091 to definitively confirm this hypothesis, it represents a compelling explanation for the potency difference and a focus for future structural studies. the species‐specific potency difference appears to stem from quantitative differences in binding affinity/efficiency influenced by the local protein structure, rather than from a qualitative difference in the mechanism of action. The core finding that P005091 acts through covalent modification of these two specific cysteines is robustly validated across both species.

The identification of P005091 (renamed NTP1) as a covalent STING CTD inhibitor provided a robust foundation for structure‐activity relationship (SAR) studies. Our focused optimization yielded significant improvements: 1) Pharmacophore Refinement: Replacing the thioether bridge with an oxygen ether (NTP14) enhanced covalent reactivity, likely by lowering the dissociation energy of the benzenethiol leaving group. Esterification at the thiophene ortho‐position (NTP16) further boosted cellular potency, particularly in murine systems (up to 20‐fold lower IC_50_ vs NTP1), potentially by optimizing interactions with a proximal hydrophobic pocket or influencing electronic effects on the nitro group. 2) Validation of Dual‐Pronged Optimization: The systematic exploration of modifications on both the covalent warhead (benzenethiol substituents: NTP2‐13) and the adjacent region (thiophene ortho‐substituents: NTP15‐21, including the O‐bridged NTP16) successfully identified compounds (NTP14, NTP16) with 5–20‐fold enhanced activity. These findings solidify the P005091 scaffold, particularly NTP16, as a promising lead candidate for anti‐inflammatory drug development. The pronounced species difference in NTP16's potency highlights the need for humanized models in further preclinical evaluation. Future translational efforts should explore strategies like colon‐targeted delivery systems to maximize local efficacy in IBD while minimizing systemic exposure, or combination therapies with agents targeting complementary pathways (e.g., anti‐integrins). The demonstrated efficacy in TREX1‐KO mice also suggests potential applicability in STING‐associated autoinflammatory disorders.

This study establishes a novel therapeutic strategy by covalently targeting the Cys292/Cys309 molecular switch within the STING CTD to disrupt its essential functional oligomerization. Leveraging the “old drug” P005091 (NTP1), we not only validated this innovative mechanism but also developed highly potent optimized derivatives, NTP14 and NTP16. Their ability to potently suppress the type I IFN inflammatory cascade and ameliorate pathology in both acute inflammation and IBD models underscores their potential as promising leads for treating STING‐driven inflammatory diseases. It should be noted, however, that preliminary pharmacokinetic (PK) characterization following intraperitoneal injection revealed that the new derivatives, such as NTP14, did not exhibit a superior bioavailability compared to P005091 (Figure S40). This limited PK profile may partly explain why the efficacy improvement in vivo was less pronounced than that observed at the cellular level. Consequently, future medicinal chemistry efforts will need to focus on optimizing the drug‐like properties of these compounds to fully translate their potent in vitro activity into improved in vivo performance. Future research will focus on: 1) Determining high‐resolution crystal structures of NTP16 bound to STING CTD to precisely map the covalent interaction and guide the rational design of next‐generation irreversible inhibitors with improved selectivity and properties. 2) Evaluating the efficacy of NTP14/NTP16 in models of STING gain‐of‐function mutations, such as those seen in SAVI (STING‐associated vasculopathy with onset in infancy), to assess their potential for treating these severe autoinflammatory conditions.

**FIGURE 8:**
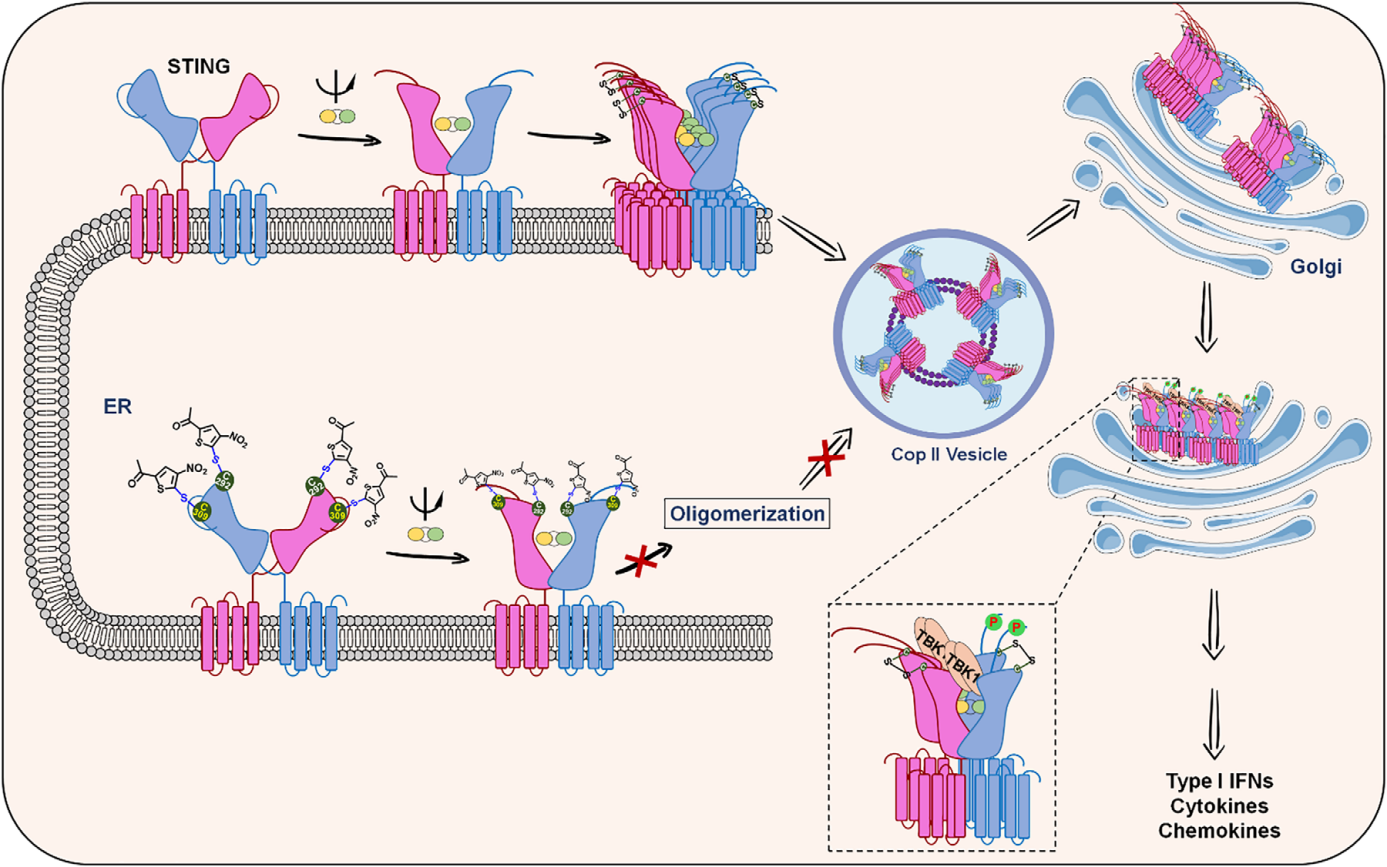
Schematic model. This study identifies a novel class of cGAS‐STING pathway inhibitors that act through covalent dual‐modification of STING at Cys292/Cys309. This action blocks disulfide bond‐mediated oligomerization and subsequent COP‐II‐dependent Golgi translocation, thereby shutting down downstream signaling. In autoimmune and inflammatory bowel disease models, the inhibitors ameliorate pathology by suppressing STING‐driven hyperproduction of I‐IFNs, cytokines, and chemokines.

The covalent targeting strategy unveiled here for STING oligomerization inhibition provides a powerful new tool for both fundamental research and the development of novel therapeutics against a spectrum of inflammatory and autoimmune diseases (Figure 8).

## 4 Materials and Methods

### 4.1 Cell Lines and Culture

RAW264.7, L929, BJ, HeLa, HeLa‐STING KO, HEK293T‐Flag STING and COS7‐HA STING cells were cultured in DMEM medium supplemented with 10% fetal bovine serum (FBS), 100 units/mL penicillin, and 100 µg/mL streptomycin. THP‐1 cells and DC2.4 cells were cultured in RPMI‐1640 medium supplemented with 10% FBS, 100 units/mL penicillin, and 100 µg/mL streptomycin. THP‐1‐Lucia ISG cells were cultured in RPMI‐1640 medium supplemented with 10% FBS, 100 units/ml penicillin, 100 µg/mL streptomycin, and 50 µg/mL zeocin. HEK293F cells were maintained in Union293 UP1000 serum‐free medium (Union‐Biotech, China). All cells were cultured at 37°C in a humidified atmosphere with 5% CO_2_. THP‐1, THP‐1 Lucia ISG and HEK293F cells were cultured in suspension using shake flasks on an orbital shaker at 120 rpm. All cell lines underwent Short Tandem Repeat (STR) profiling with reports provided by the vendors, and tested negative for both sterility and mycoplasma contamination. Cells were frozen in liquid nitrogen and used for experiments at passages 3 to 10 after thaw. The catalog number, Research Resource Identifier (RRID), source, and date of acquisition for all cell lines are detailed in Table S2 of the Supplementary Information.

### 4.2 Plasmid Transfection

HEK293F cells were resuspended at a density of 2 × 10^6^ cells/mL and allocated to cell culture shake flasks, yielding a total of 2 × 10^7^ cells per flask. Cells were transfected with 10 µg of the following plasmids: Flag‐hSTING (WT, C12A, C29A, C64A, C88A, C91A, C148A, C206A, C257A, C292A, C309A, CS), HA‐hSTING (WT, C206/309A, C257/309A, C292/309A), Flag‐mSTING (WT). Transfection was performed using PEI 40 000 (ApexBio, USA) according to the manufacturer's protocol. Transfected cells were cultured for 48 h, and the culture medium was replaced with fresh medium containing the indicated drugs for treatment.

HeLa‐STING KO or HEK293T cells (3 × 10^6^ cells per dish) were seeded in a 10‐cm cell culture dish and allowed to adhere. Adherent cells were transfected with 10 µg of the following plasmids: HA‐hSTING (WT, C206/309A, C257/309A, C292/C309A), HA‐mSTING (WT, C291/309A) and Myc‐USP7. Transfection was performed using PEI 40 000 according to the manufacturer's instructions. After culturing for 24 h, cells were trypsinized, resuspended, and seeded into 6‐well plates at a density of 8 × 10^5^ cells per well. After 24 h of culture, cells were subjected to drug treatments.

### 4.3 Biotin Pulldown Assay

HEK293F or HeLa‐STING KO cells (2 × 10^7^ cells per flask) were seeded into cell culture shake flasks at a density of 2 × 10^6^ cells/mL. Cells were transiently transfected with plasmids encoding the following constructs: Flag‐tagged human STING (hSTING) and its mutants: WT, C12A, C29A, C64A, C88A, C91A, C148A, C206A, C257A, C292A, C309A and all CS; Flag‐tagged murine STING (mSTING): WT; HA‐tagged human STING (hSTING) and its mutants: WT, C206/309A, C257/309A, C292/309A. Transfected cells were cultured on an orbital shaker at 130 rpm for 48 h, then P005091‐Biotin (0–20 µM for HEK293F‐Flag hSTING, 20 µM HeLa‐STING KO‐ HA hSTING, 0–40 µM for HEK293F‐Flag mSTING) was added for 2 h, or subsequently harvested by centrifugation, and the supernatant was discarded. The HEK293F cell pellet was lysated in cell lysis buffer, and the lysate was clarified by centrifugation at 13 000 rpm for 10 min at 4°C. HEK293T‐Flag STING cells treated with P005091‐Biotin were directly washed with PBS buffer and lysated, centrifuged using the same method.

Protein concentration in the supernatant was determined using a BCA assay kit. For each sample, 1.5 mg of total protein was incubated with 1.5 µL of the corresponding anti‐Flag or anti‐HA antibody on a rotary shaker at 4°C for 12 h. Protein A/G agarose beads (Beyotime, China) were then added, and incubation was continued on the rotary shaker at 4°C for 2 h. The bead‐antibody‐protein complexes were pelleted by centrifugation at 2500 rpm for 3 min at 4°C. For HEK293F cells lysis samples, the beads were washed three times with cell lysis buffer and resuspended followed by division into two equal aliquots. P005091‐Biotin (40 µM final concentration) or an equivalent volume of DMSO (vehicle control) was added to the respective aliquots, and samples were incubated on the rotary shaker at 4°C for 3 h (for the samples used in the intracellular P005091‐Biotin dose‐response experiment, it is no need to add P005091‐Biotin for incubation at this step). Following incubation, the beads were pelleted by centrifugation at 2500 rpm for 3 min at 4°C and washed three times with cell lysis buffer. Bound proteins were eluted by resuspending the beads in 1×SDS‐PAGE loading buffer (diluted in PBS) and heating at 95°C for 10 min. For HEK293T‐Flag STING cells lysis samples, beads were directly resuspended in 1×SDS‐PAGE loading buffer and heated.

For the in vitro incubation assay of the STING‐CTD recombinant protein, 15 µL (12 µg) of N‐Sumo‐6His‐STING protein was mixed with P005091‐Biotin (0–40 µM) at 37°C for 3 h; or mixed with P005091‐Biotin (10 µM) at 37°C for 0–24 h; or mixed with P005091‐Biotin (0–20 µM) at 37°C for 2 h following by treated with succinimide (500 µM, 1 h).Subsequently, 5 × loading buffer was added to the reaction mixture prior to heating at 95°C for 10 min.

### 4.4 Immunoblotting Assays and Data Analysis

BJ and HeLa cells (1 × 10^6^) were seeded in 6‐well plates and allowed to adherd, THP‐1 cells (1.2 × 10^6^) were seeded in 6‐well plates. Then pretreated with P005091(0–40 µM) for 2 h, then co‐incubated with diABZI (1 µM) for another 2 h, or pretreated with NTP14, NTP16 (0–20 µM) for 2 h, then co‐incubated with diABZI (1 µM) for another 2 h. For time gradient, BJ/HeLa cells were pretreated with P005091 (20 µM), then washed with PBS and treated with diABZI (1 µM) for the indicated time points (0.25/0.5/0.75/1/2 h). 1 × 10^6^ RAW264.7/DC2.4 cells were seeded and adhered, the cells were then pretreated with P005091 (0–1000 nM or 0–10 µM) for 2 h, then co‐incubated with diABZI (5 µM) or S‐cddA (0.5 µM) for another 2 h. THP‐1 and RAW264.7 cells were pretreated with P005091‐Biotin (0–40 µM, 2 h), then co‐incubated with diABZI (1 or 10 µM) for another 2 h.

Total protein was extracted using Western and IP lysis buffer (NCM Biotech, China) with 1× phosphatase inhibitors and 1× PMSF. The cell suspension was centrifuged at 13 000 rpm for 15 min at 4°C. The supernatant was subjected to protein quantification using a BCA assay kit (NCM Biotech, China). The supernatant was mixed with 5× reducing loading buffer (NCM Biotech, China) or 5× denaturing non‐reducing loading buffer (Biosharp, China), mixed thoroughly, and heated at 95°C for 10 min to prepare the protein sample for SDS page analysis. The supernatant was mixed with 5× non‐denaturing non‐reducing loading buffer (NCM Biotech, China), mixed thoroughly, and used directly to prepare the protein sample for native page analysis. Following protein separation by gel electrophoresis, proteins were transferred to a PVDF membrane. The membrane was then incubated with the corresponding primary antibody (STING, Phospho‐STING, TBK1, Phospho‐TBK1, vinculin, GAPDH, Biotin, HA, DYKDDDDK, Myc diluted at 1:1000) at 4°C for 12 h, followed by incubation with a horseradish peroxidase‐conjugated secondary antibody at room temperature for 2 h. The protein bands were visualized using FDbio‐Dura ECL Kit (NCM Biotech, China) and quantitatively analyzed by ImageJ software.

To enhance the quantitative reliability of Western blot analyses across all experimental conditions, we implemented a rigorous semi‐quantitative correction approach. Grayscale quantification for all key protein targets (e.g., p‐STING, p‐TBK1, and STING Oligomerization) was performed consistently, regardless of the sample preparation system used‐including reducing, non‐reducing, and native conditions. For each target, analysis was conducted using images captured at multiple exposure times within the linear detection range, and data were normalized to respective loading controls. Dose response curves and subsequent IC_50_ calculations were derived from this corrected and normalized dataset, based on at least three independent biological replicates. Crucially, all conditions for a given experiment were processed and analyzed on the same membrane to ensure internal consistency and minimize technical variability. Reported IC_50_ values are presented with significant figures that appropriately reflect the precision of the bioassay.

### 4.5 Mice and In Vivo Studies

Female C57BL/6J mice (Stock No: N000013) at 4 or 6 weeks of age and female C57BL/6J Trex1^−^/^−^ mice (Stock No: TO13987) at 6 weeks of age were purchased from GemPharmatech Co., Ltd (Nanjing, China). Animal experiments were approved by the Animal Welfare and Ethics Committee of Jinan University. The ethical Approval Number is 20240227‐0076.

### 4.6 CMA‐Induced Acute Inflammation Model

C57BL/6J mice at 6 weeks were randomly divided into four groups: control group, CMA group, P005091 group, and P005091 + CMA group. Mice in the treatment groups were intraperitoneally injected with P005091 (2.5 or 10 mg/kg), dissolved in 3% DMSO and diluted with HS‐15, and finally prepared in 90% NS solution, which was followed, 3 h later, by an injection of CMA (224 mg/kg, dissolved in 3% DMSO and diluted with HS‐15, and finally prepared in 90% NS solution). After 4 h CMA injection, the mice were euthanized. Heart, liver, spleen, lung, kidney, tongue, stomach, skeletal muscle, and blood samples were collected. Organs were fixed in 4% paraformaldehyde, sectioned, and subjected to H&E staining, immunohistochemistry (IHC), and inflammation scoring. Serum levels of IFN‐β1, CXCL10, and IL‐6 were quantified using commercial ELISA kits (JONLNBIO, China) according to the manufacturer's instructions.

### 4.7 Trex1^−^/^−^ Spontaneous Inflammatory Model

C57BL/6J Trex1^−^/^−^ mice and their Trex1^+^/^−^ (or wild‐type) littermate controls at 4 weeks of age were randomly assigned to receive either vehicle or P005091. The treatment group received daily intraperitoneal injections of P005091 (5 mg/kg) for 10 consecutive days. On day 11, all mice were euthanized. Heart, liver, spleen, lung, kidney, tongue, stomach, skeletal muscle, and blood were collected. Tissues were fixed, sectioned, stained, and evaluated for inflammation. Serum levels of IFN‐β1, CXCL10, and IL‐6 were quantified using commercial ELISA kits (JONLNBIO, China) according to the manufacturer's instructions. Total RNA in heart and stomach was extracted using a FastPure Cell/Tissue Total RNA Isolation Kit V2 (Vazyme, China) according to the manufacturer's protocol.

### 4.8 DSS‐Induced Acute Colitis Model

C57BL/6J mice at 6 weeks were randomly divided into four groups: control, DSS, drug‐only (including separate groups for P005091, NTP14, and NTP16), and DSS + drug (similarly including separate combination groups for each compound). Mice in the DSS‐exposed groups (i.e., DSS and DSS + drug groups) received 3% DSS in drinking water ad libitum for 11 days. Mice in the compound‐treated groups received daily intraperitoneal injections of the respective compound (P005091, NTP14, or NTP16) at 5 mg/kg of drugs intraperitoneally each day for 10 days. Body weight was recorded daily. On day 12, all mice were euthanized. Colon length was measured, and colon tissues were collected and fixed in 4% paraformaldehyde for subsequent H&E and IHC staining, pathological scoring, and inflammatory evaluation. Serum levels of IFN‐β1, CXCL10, and IL‐6 were quantified using commercial ELISA kits (JONLNBIO, China) according to the manufacturer's instructions.

### 4.9 Statistical analysis

All statistical analysis was performed using GraphPad Prism 8.0 or SPSS 20.0 software. Differences between groups with dose factors or with more than one variation were determined by two‐way analysis of variance (ANOVA).

Additional methods and spectrograms of the synthesized compounds can be found in Supplementary Information. Information of the small molecule compounds involved in the mass spectrometry‐based screening can be found in Table S1. All information regarding reagents, plasmids, antibodies, commercial assays and cell constructs can be found in Table S2. Abbreviations can be found in Table S3.

## Author Contributions

All authors participated in data acquisition. X. Yue, X. Bu, S. Zhang, and J. Quan contributed to the conception and design of the study. Y. Zhao, L. Huang, W. Qin, B. Zhang, Y. Yang, X. Chen, Xiaoquan Wang, W. Zhou, Z. Xiang, F. Chen, Z. Li, L. Le, Y. Zhang, L. Zhang, F. Wang, and D. Lei did data analysis and interpretation. Z. Cai, Y. Gao, Y. Chen, and Xuecen Wang supervised the project. X. Yue, X. Bu, S. Zhang, J. Quan, Y. Zhao, L. Huang, and W. Qin contributed to the drafting and revision of the manuscript. All authors read and approved of the final manuscript.

## Funding

The National Key Research and Development Program of China (2023YFF1204600 to S. Zhang); This project was supported by the National Natural Science Foundation of China (NSFC) (82573441, 82272743 to X. Yue, 82227802 to S. Zhang, 82373713 to X. Bu, 82373209 to Xuecen Wang, 82302306 to L. Zhang, 82373203 to Y. Chen); The Science and Technology Projects in Guangzhou (202201020022 to S. Zhang, 2023A03J1036 to S. Zhang, 2023A03J1038 to F. Wang, 2025A04J0177 to X. Yue, 2024A04J4633 to Xuecen Wang, 2025A04J7006 to B. Zhang); Open Competition to Select the Best Candidates Key Technology Program for Nucleic Acid Drugs of National Technology Innovation Center for Biopharmaceuticals (NCTIB2022HS03001 to W. Zhou).The Kelin Rising Star Talent Program from The First Affiliated Hospital of Sun Yat‐sen University (R08039 to Xuecen Wang); The Science and Technology Youth Talent Nurturing Program of Jinan University (21623209 to B. Zhang); The Natural Science Foundation of Jiangxi Province (20232BAB206094 to W. Qin).

## Conflicts of Interest

The authors declare no conflicts of interest.

## Supporting information




**Supporting File**: advs74967‐sup‐0001‐SuppMat.docx.

## Data Availability

The data that support the findings of this study are available from the corresponding author upon reasonable request.
