# Supplementary material for: Dual Covalent Targeting of STING Cysteines 292/309 Disrupts Functional Oligomerization and Enables Potent Antagonist Development
Source: Adv Sci (Weinh). 2026 Mar 24;13(32):e22764. doi: 10.1002/advs.202522764 (PMC13252623; doi:10.1002/advs.202522764)
Supplement: Supplementary file 1 — Supporting File: advs74967‐sup‐0001‐SuppMat.docx. [file ADVS-13-e22764-s001.docx]

**Supplementary Information for**

**Dual covalent targeting of STING cysteines 292/309 disrupts functional oligomerization and enables potent antagonist development**

Yuxuan Zhao, Ling Huang, Wenjing Qin, Bin Zhang, Yang Yang, Xue Chen, Xiaoquan Wang, Weilin Zhou, Feiyang Chen, Zhenyu Li, Liyuan Le, Yiqiu Zhang, Zhen Xiang, Lu Zhang, Fei Wang, Dan Lei, Zi-zhe Cai, Ying Gao, Yong Chen, Xuecen Wang, Junmin Quan*, Shuixing Zhang*, Xianzhang Bu*, Xin Yue*

*Email: [quanjm@pku.edu.cn](mailto:quanjm@pku.edu.cn) (J.Q.); [shui7515@126.com](mailto:shui7515@126.com) (S.Z.); [phsbxzh@mail.sysu.edu.cn](mailto:phsbxzh@mail.sysu.edu.cn) (X.B.); [yuex2504@jnu.edu.cn](mailto:yuex2504@jnu.edu.cn) (X.Y.)

**This file contains:**

**Supplementary Figures S1-S40**

**Supplementary Table S1: Information of compounds for LC/MS based screening**

**Supplementary Methods**

**Supplementary Table S2: Information of reagents and resources**

**Supplementary Table S3: Abbreviations**

**Spectrogram for structural characterization of new compounds (^1^H NMR and HRMS)**

**Supplementary Figures**

**Figure S1**


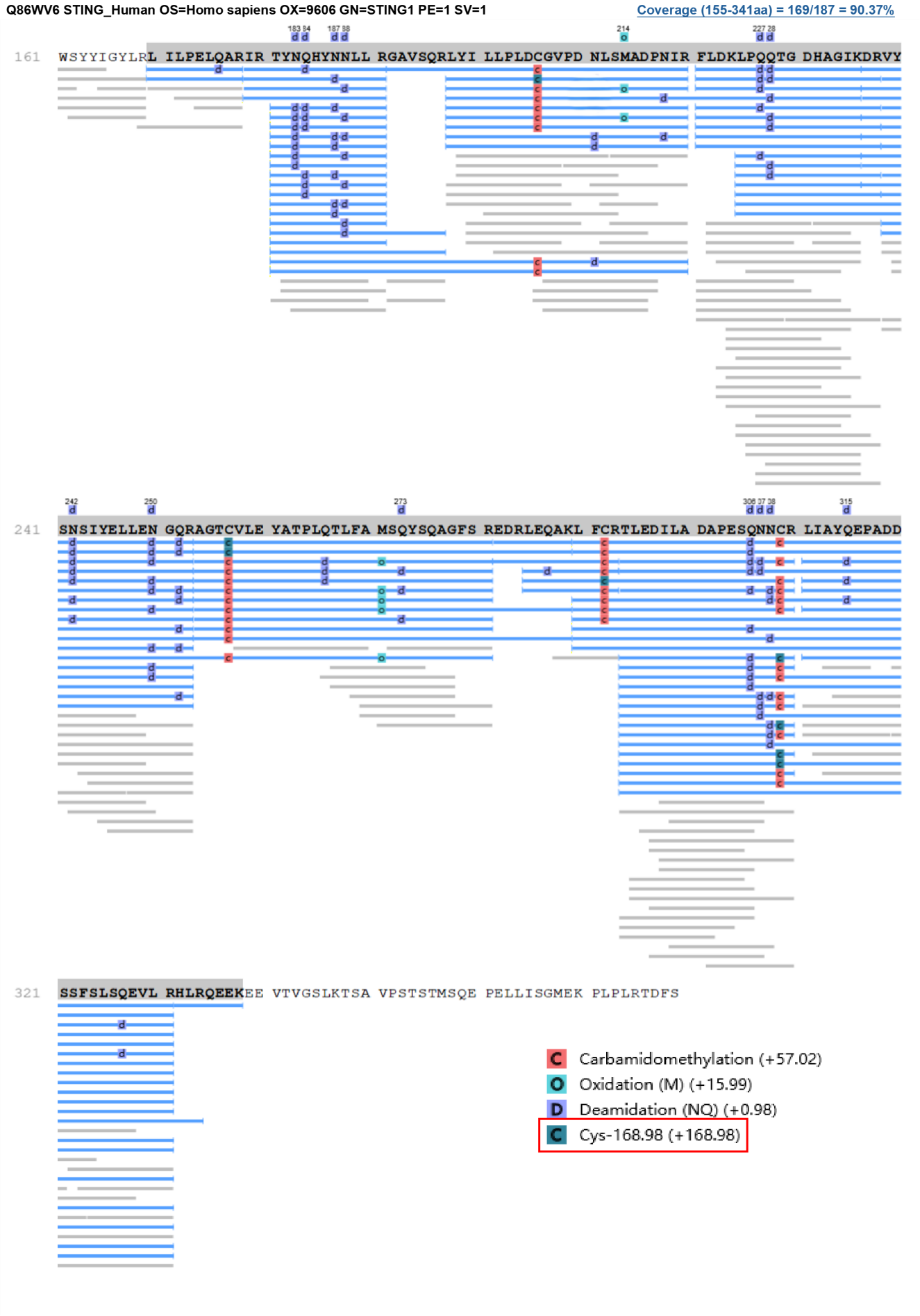


**Figure S1: Overview of mass spectrometry-detected peptides for STING-CTD.**Human STING-CTD protein was co-incubated with P005091 (20 μM) and subjected to mass spectrometry analysis. Carbamidomethylation (+57.02), oxidation (+15.99) and deamidation (+0.98) represent common modifications occurring during sample preparation. Cys-168.98 (+168.98) indicates the expected molecular mass shift corresponding to P005091 modification on a cysteine residue, which was highlight by red a rectangle. Coverage (155-341aa)=90.37%.

**Figure S2**

**Figure S2: Details of mass calculations for peptides covalently modified by P005091.**Region highlighted in red: Mass calculations for the peptide containing covalent modification at Cys309, showing matching y6, y8, y9, y10, and y12 ions. Region highlighted in green: Mass calculations for the peptide containing covalent modification at Cys206, showing matching y18 and y19 ions. Region highlighted in orange: Mass calculations for the peptide containing covalent modification at Cys292, showing matching b9 ion.

**Figure S3:**

**
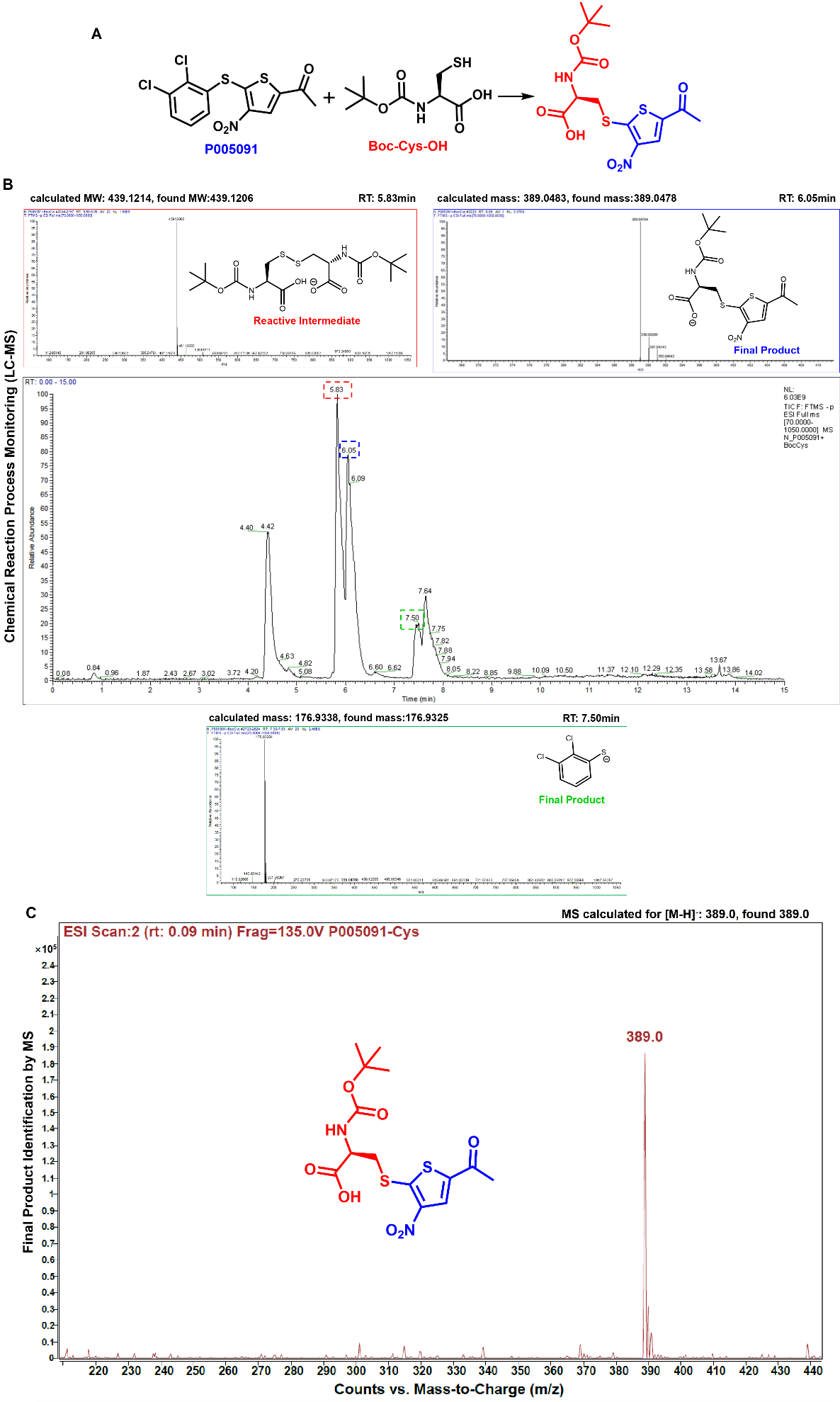
**

**Figure S3: Identification of the reaction product between P005091 and Boc-Cys-OH.** (**A**) Schematic diagram of the reaction between P005091 and Boc-Cys-OH. (**B**) LC-MS analysis of the reaction mixture. Structural assignment was based on the mass-to-charge ratio (m/z) of the detected negative ion peak. (**C**) Mass spectrometry data of the isolated and purified reaction product.

**Figure S4:**


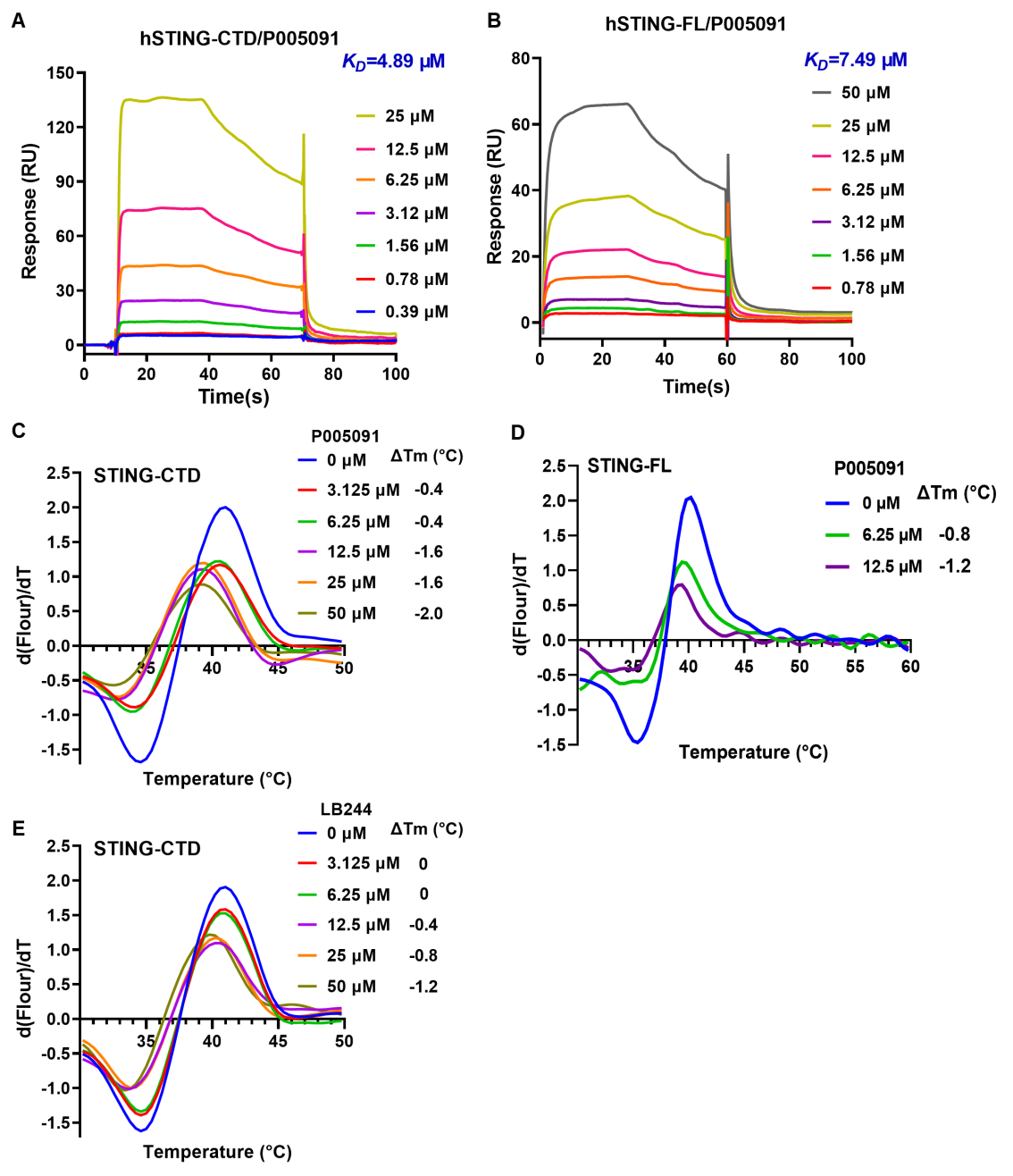


**Figure S4: Interaction between P005091 and STING-CTD and STING-FL. (A-B)** SPR analysis of P005091 with hSTING-CTD protein (**A**) and hSTING-FL protein (**B**). (**C-E**) Detection of ΔTm (℃) values by differential scanning fluorimetry (DSF) assay for co-incubation of P005091 (0-50 μM or 0-12.5 μM) and LB244 (0-50 μM) with hSTING-CTD protein (**C, E**) and hSTING-FL protein (**D**).

**Figure S5**


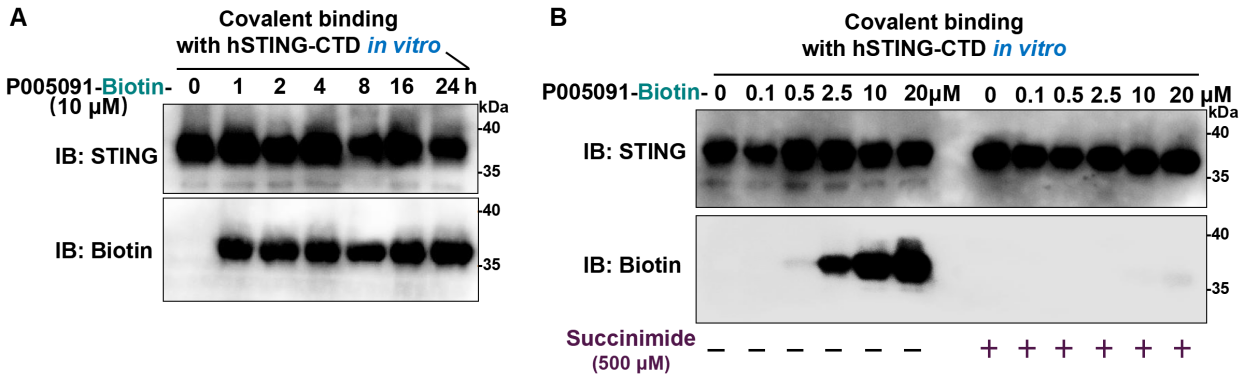


**Figure S5: Verification of covalent binding between P005091 and STING *in vitro*.** (**A**) Time-course analysis of covalent binding between P005091-Biotin and STING-CTD protein. (**B**) Concentration-dependent binding analysis of P005091-Biotin to STING-CTD protein following pretreatment with succinimide (1 hour).

**Figure S6**


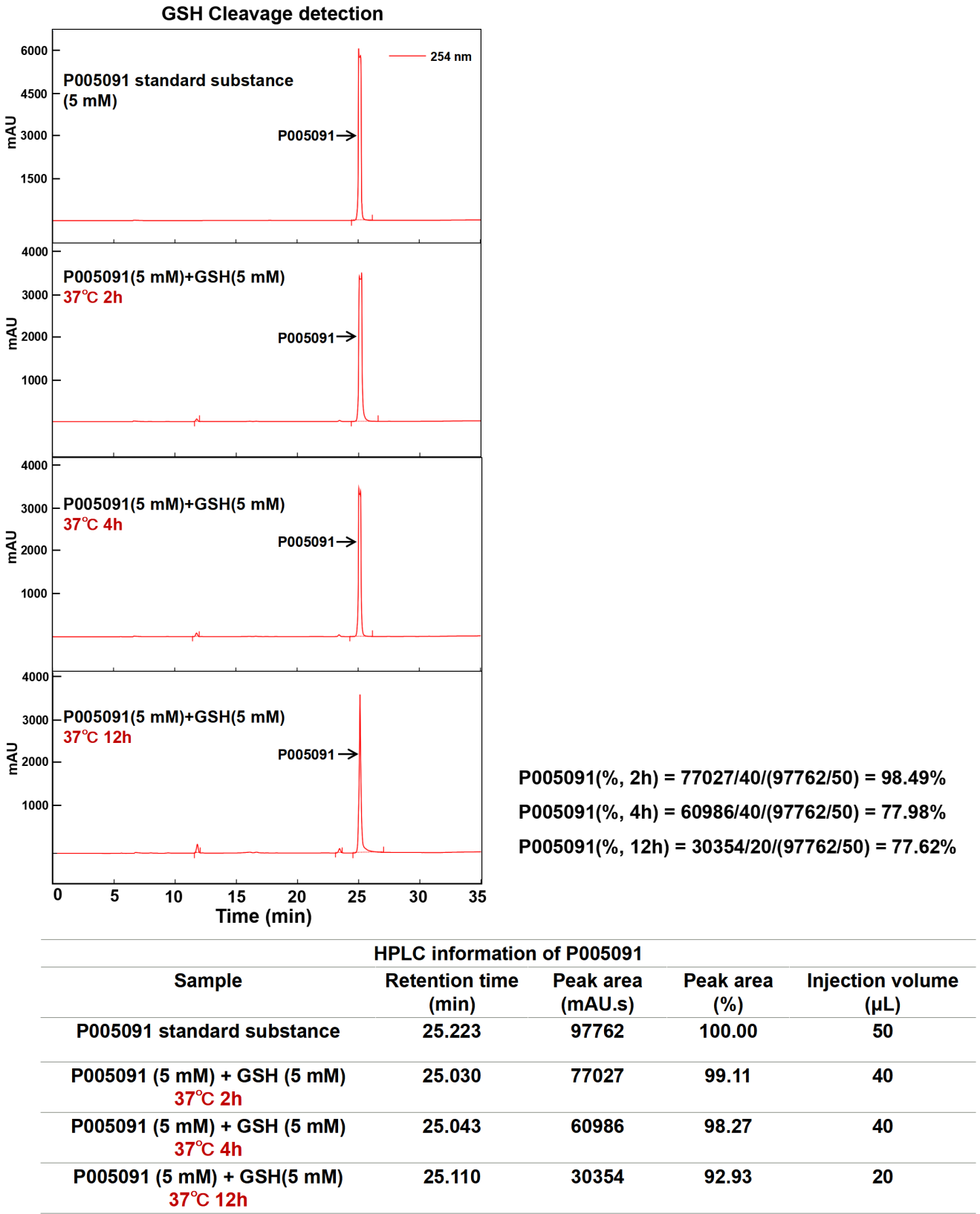


**Figure S6: Analysis of the stability of the P005091-glutathione conjugate.** HPLC analysis of the remaining amount of P005091 in the P005091-glutathione co-incubation system. Quantification of P005091 content after 2 hours, 4 hours and 12 hours of co-incubation using the peak area normalization method.

**Figure S7**


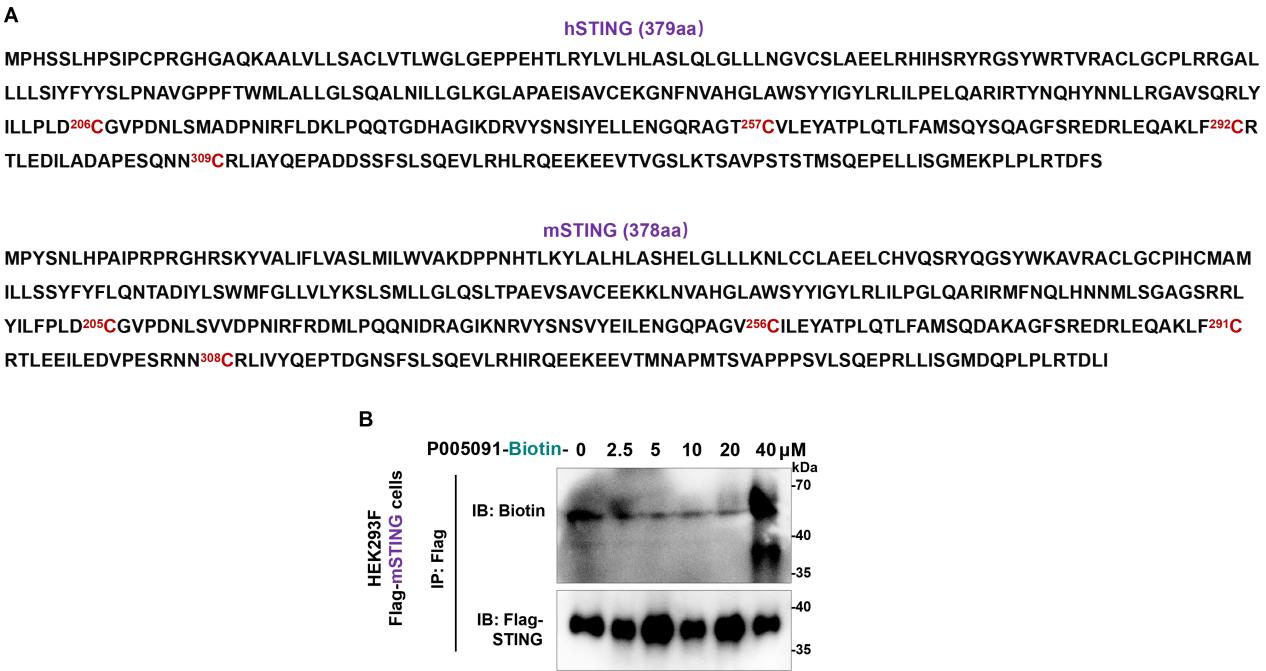


**Figure S7: Binding of P005091 to STING *in vitro*.**(**A**) Amino acid sequences of human STING (hSTING) and murine STING (mSTING). Cysteine residues within the CTD domain are highlighted in red. (**B**) HEK293F cells were transfected with Flag-mSTING plasmid. Cell lysates were subjected to immunoprecipitation using an anti-Flag antibody, followed by incubation with P005091-Biotin (0-40 μM). The samples were analyzed by WB.

**Figure S8**


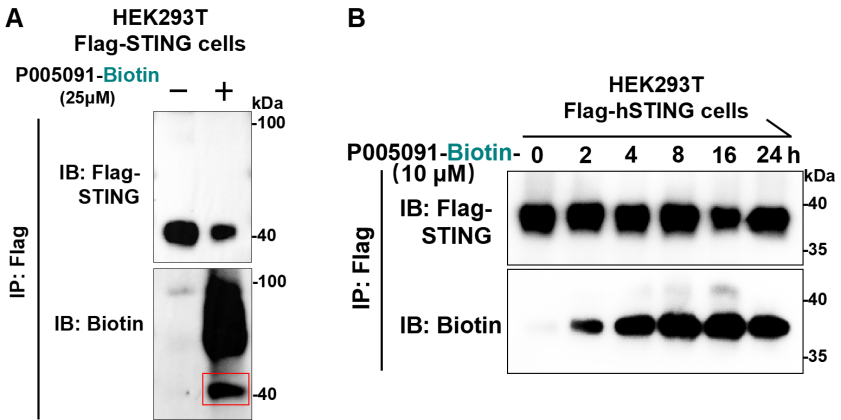


**Figure S8: Analysis of intracellular covalent binding of P005091 to STING.** (**A**) HEK293T cells transfected with Flag-hSTING. After treatment of P005091-Biotin (2 hours), cell lysates were subjected to immunoprecipitation using an anti-Flag antibody, followed by analysis with WB. (**B**) HEK293T cells transfected with Flag-hSTING. After treatment of P005091-Biotin (0-24 hours), cell lysates were subjected to immunoprecipitation using an anti-Flag antibody, followed by analysis with WB.

**Figure S9**


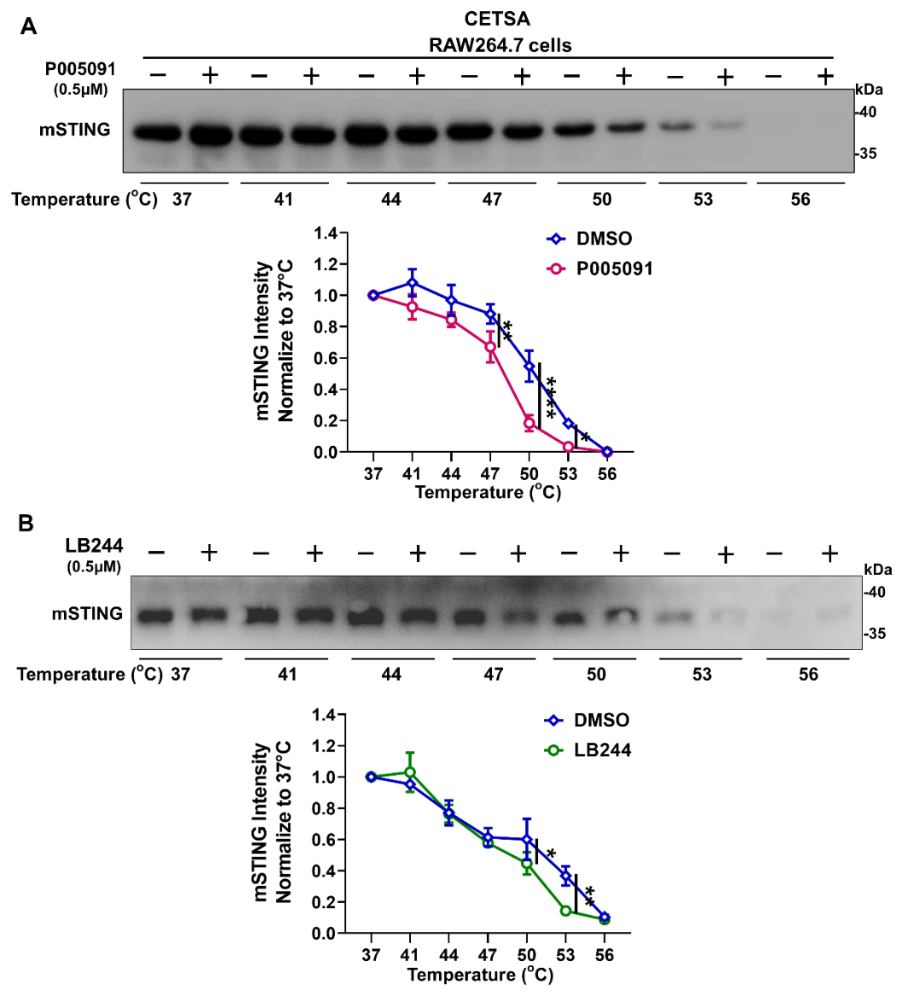


**Figure S9: Analysis of intracellular STING protein thermal stability modulated by P005091 and LB244.**(**A-B**) Cellular thermal shift assay of RAW264.7 cells treated with P005091 (**A**) or LB244 (**B**) for 1.5 hours. Top panel: WB bands of protein samples. Bottom panel: Quantiﬁcations of grayscale values. Data were represented as mean ± S.D. (n = 3). Statistical signiﬁcance was determined by two-way ANOVA (*, P < 0.05; **, P < 0.01; ****, P < 0.0001).

**Figure S10**


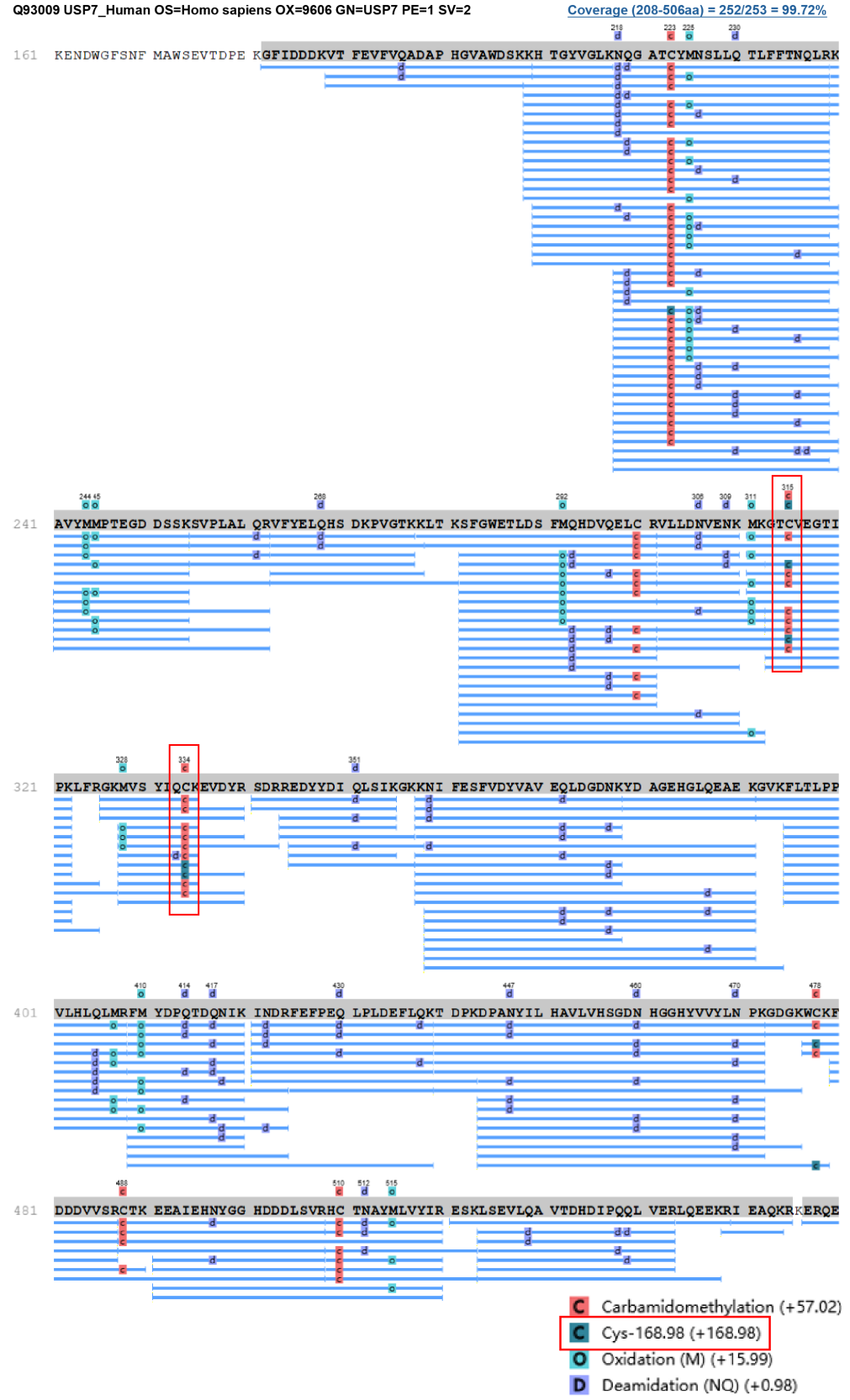


**Figure S10: Mass spectrometry coverage map of USP7 protein after P005091 treatment.** Incubation of USP7 protein (208-560aa) with P005091 (40 μM) at 37 °C for 3 hours, followed by mass spectrometry detection of covalent modifications. Carbamidomethylation (+57.02), oxidation (+15.99) and deamidation (+0.98) represent common modifications occurring during sample preparation. Cys-168.98 (+168.98) indicates the expected molecular mass shift corresponding to P005091 modification on a cysteine residue, which was highlight by a red rectangle. Coverage (208-560aa)=99.72%.

**Figure S11**


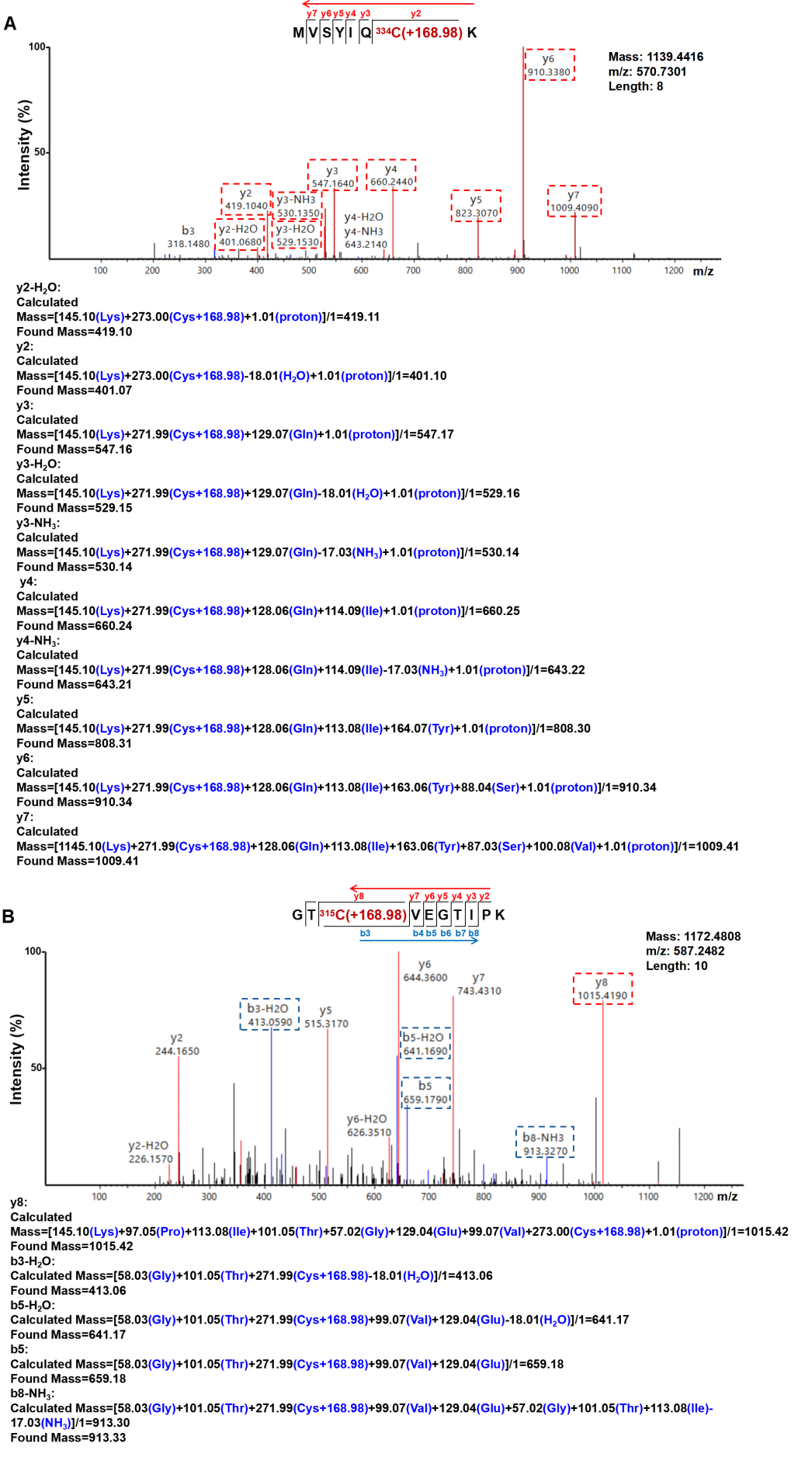


**Figure S11: Details of mass calculations for peptides of USP7 covalently modified by P005091.**(**A**) Region highlighted in red: Mass calculations for the peptide containing covalent modification at Cys334, showing matching y2, y3, y4, y5, y6 and y7 ions. (**B**) Region highlighted in red: Mass calculations for the peptide containing covalent modification at Cys315, showing matching y8 ions. Region highlighted in blue: Mass calculations for the peptide containing covalent modification at Cy315, showing matching b3, b5 and b8 ions.

**Figure S12**


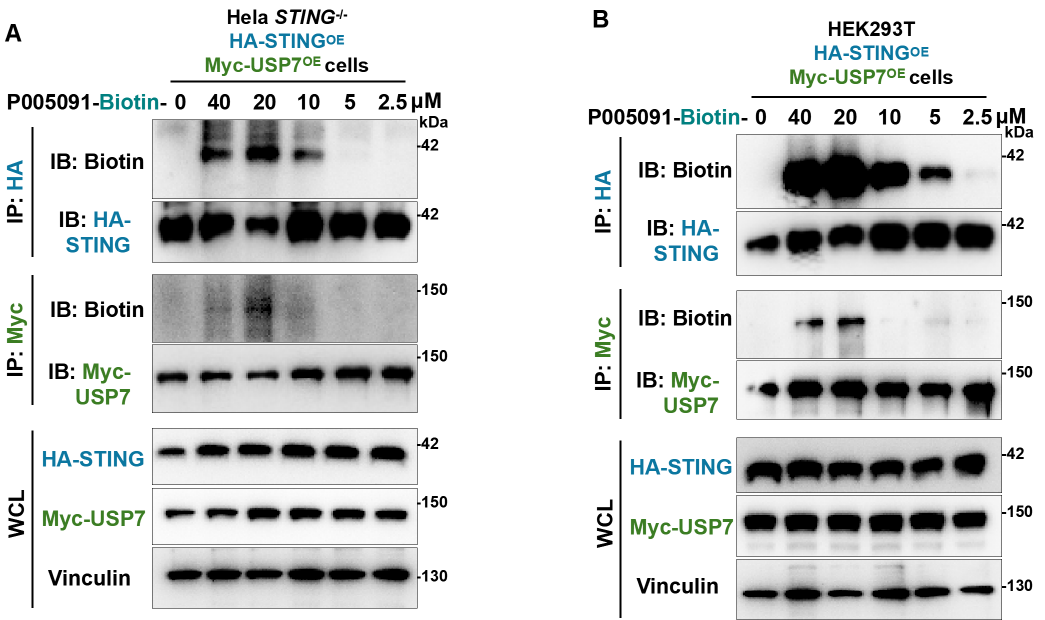


**Figure S12:** **Intracellularly Dose-response covalent binding analysis of P005091 on STING and USP7.** (**A-B**) Co-transfection of HA-hSTING and Myc-USP7 plasmids into HeLa-STING KO cells (**A**) and HEK293T cells (**B**). Treatment with P005091-Biotin (0-40 μM) for 2 hours, followed by immunoprecipitation of total protein using anti-HA and anti-Myc antibodies respectively. Samples analyzed by WB.

**Figure S13**


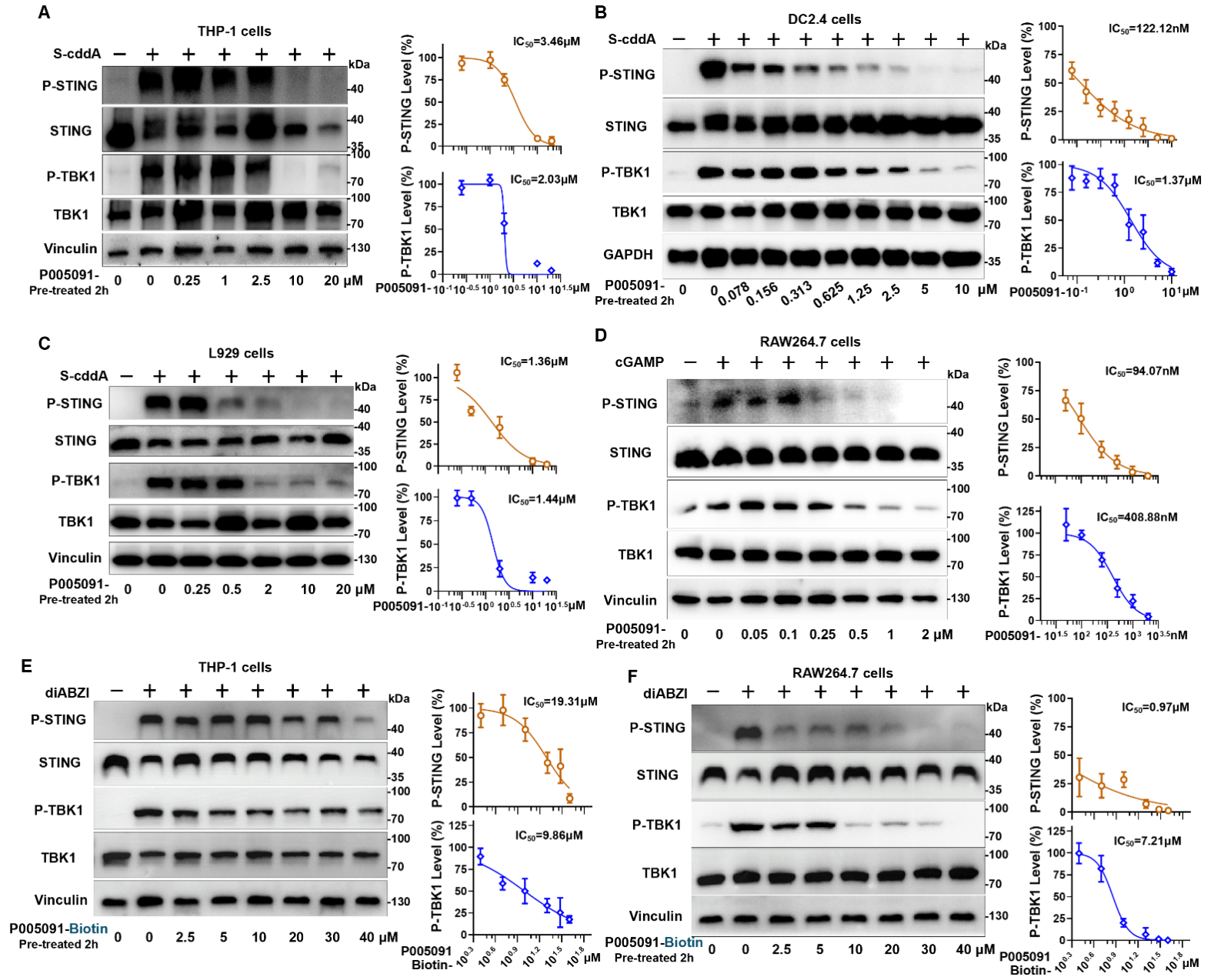


**Figure S13: Evaluation of the inhibitory efficacy of P005091 and P005091-Biotin on the cGAS-STING signaling pathway.**(**A-D**) Dose-dependent inhibitory effects of P005091 on STING pathway activation induced by S-cddA (**A**, 0.5 μM in L929 cells; **B**, 0.5 μM in DC2.4 cells; **C**, 0.1 μM in THP-1 cells) and cGAMP (**D**, 1 μM in RAW264.7 cells). (**E**) Dose-dependent inhibitory effects of P005091-Biotin on STING pathway activation induced by diABZI (1 μM) in THP-1 cells. (**F**) Dose-dependent inhibitory effects of P005091-Biotin on STING pathway activation induced by diABZI (10 μM) in RAW264.7 cells. Quantiﬁcations of grayscale values data were represented as mean ± S.D. (n = 3).

**Figure S14**


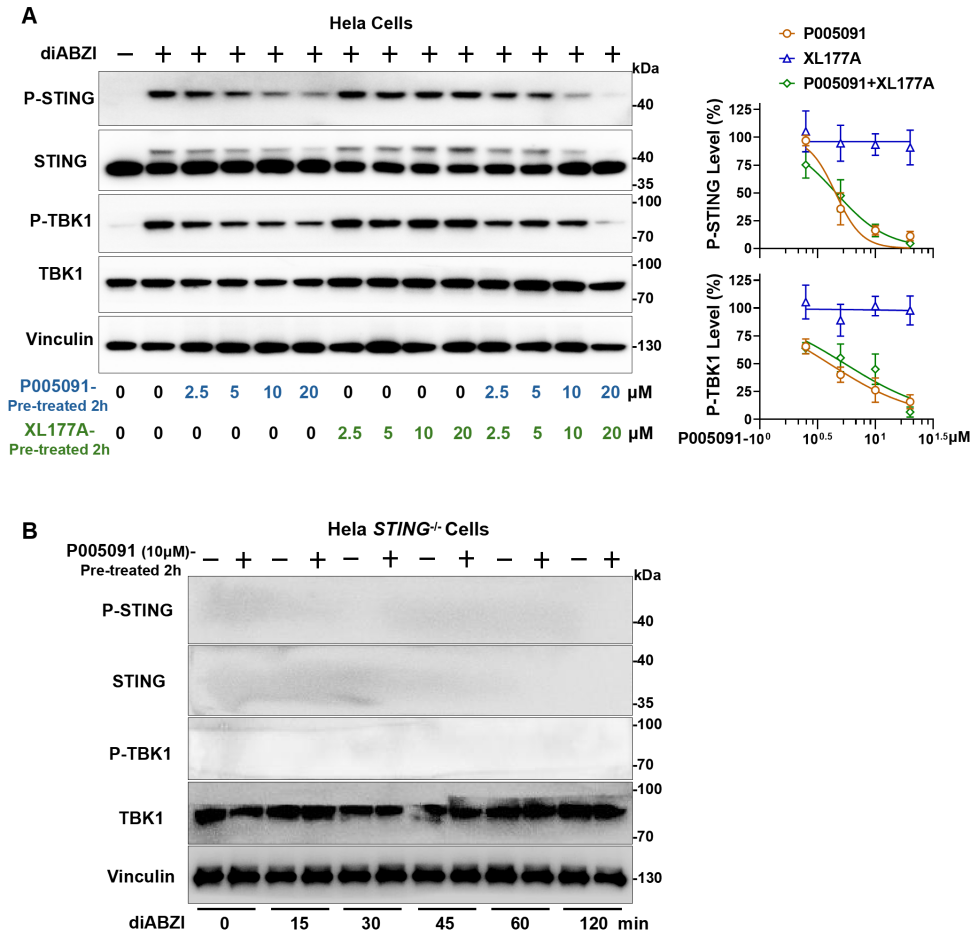


**Figure S14: Analysis of specific STING pathway dependency for P005091 inhibitory activity.** (**A**) Dose-dependent inhibitory effects of P005091, XL177A, and their combination on STING pathway activation induced by diABZI (1 μM) in HeLa cells. Quantiﬁcations of grayscale values data were represented as mean ± S.D. (n = 3). (**B**) Time-course analysis of STING pathway activation by diABZI(1 μM) and its inhibition by P005091 in HeLa-STING KO cells.

**Figure S15**


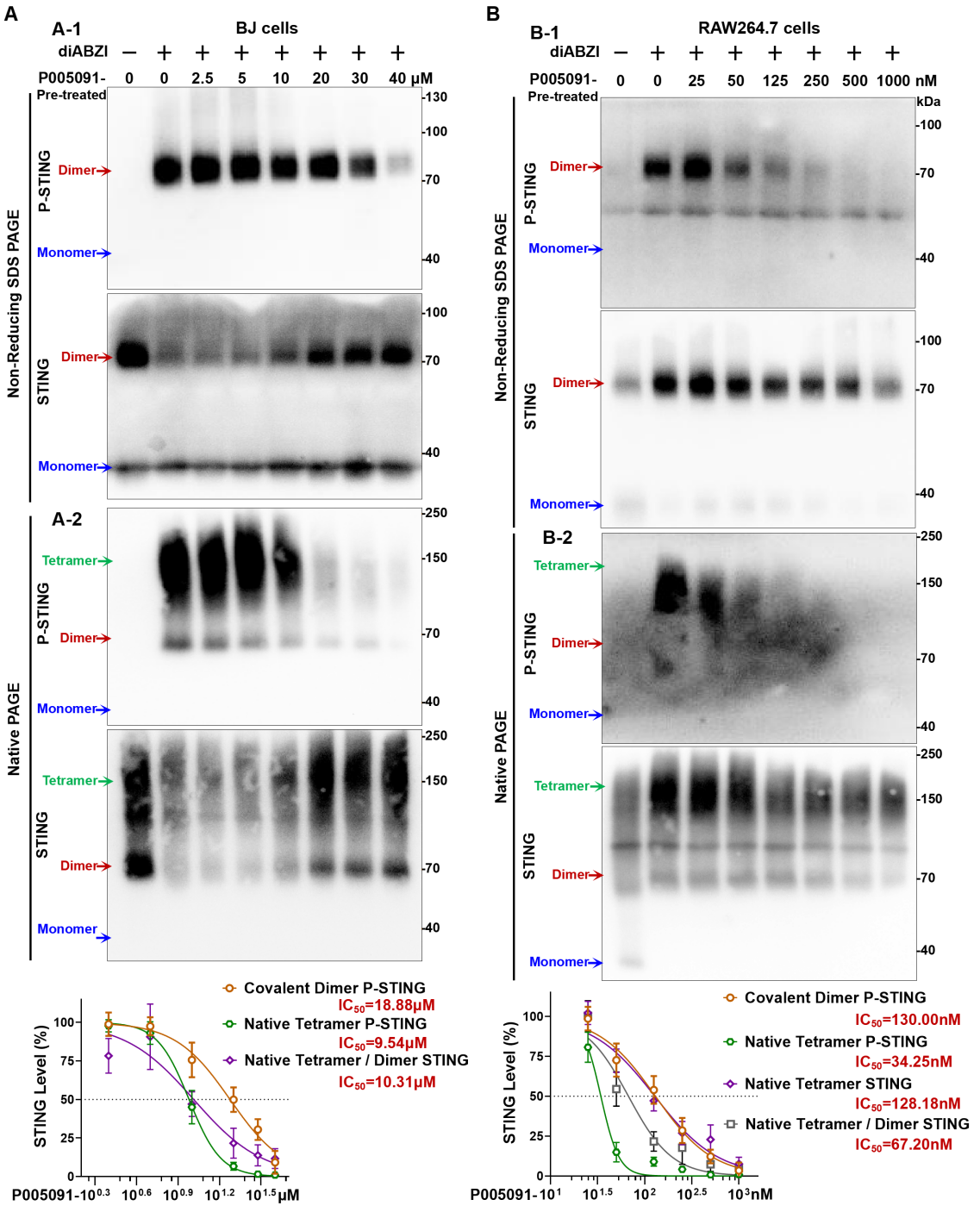


**Figure S15: P005091 inhibits oligomeric STING and P-STING formation in BJ (A) and RAW264.7 (B) cells.** Inhibition of diABZI (10 μM, 2 hours pretreated)-induced dimer and tetramer formation of STING and P-STING by P005091 at the indicated concentrations. Top panel: Non-reducing SDS-PAGE and Native PAGE protein bands detecting STING and P-STING. Bottom panel: Quantiﬁcations of grayscale values for dimer P-STING, tetramer STING and tetramer P-STING. Data were represented as mean ± S.D. (n = 3).

**Figure S16**


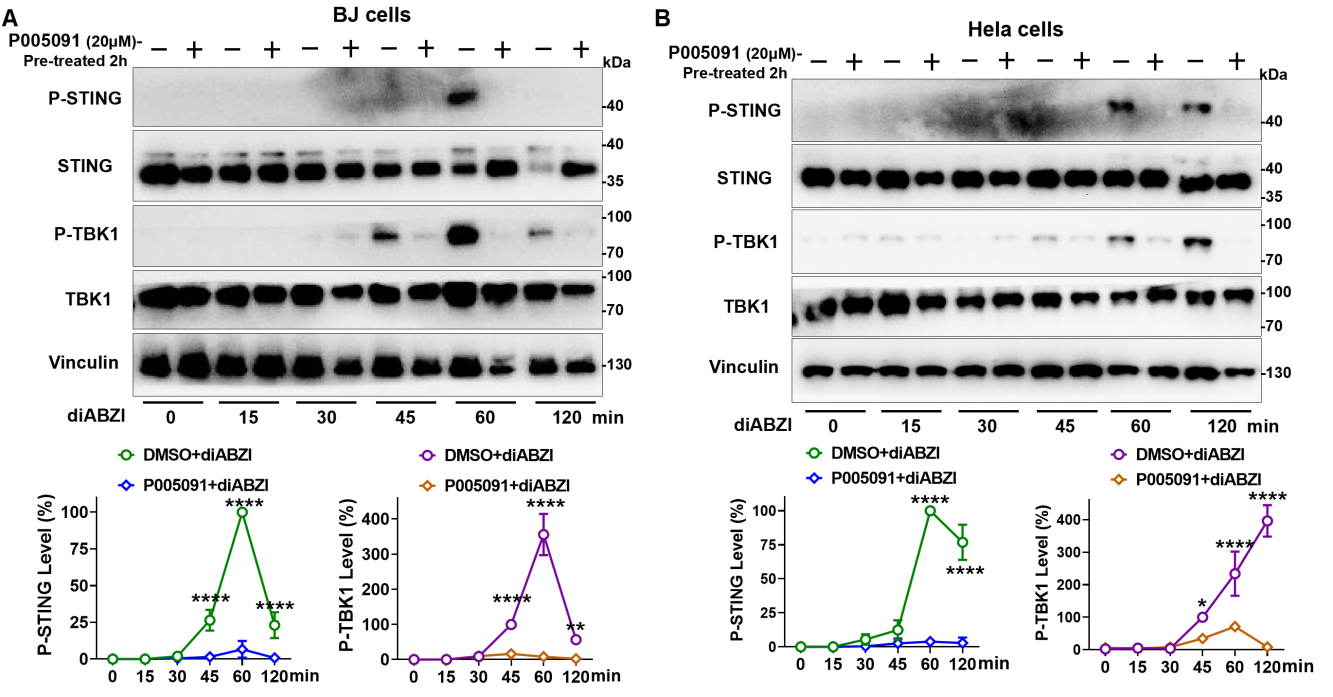


**Figure S16: Time course analysis of P005091 inhibition of the STING pathway.** (**A-B**) Kinetic analysis of diABZI (1 μM)-induced STING activation and its inhibition by P005091 over a 0-2 hour time course in BJ cells (**A**) and HeLa cells (**B**). Data were represented as mean ± S.D. (n = 3). Statistical signiﬁcance was determined by two-way ANOVA (*, P < 0.05; **, P < 0.01; ****, P < 0.0001).

**Figure S17**


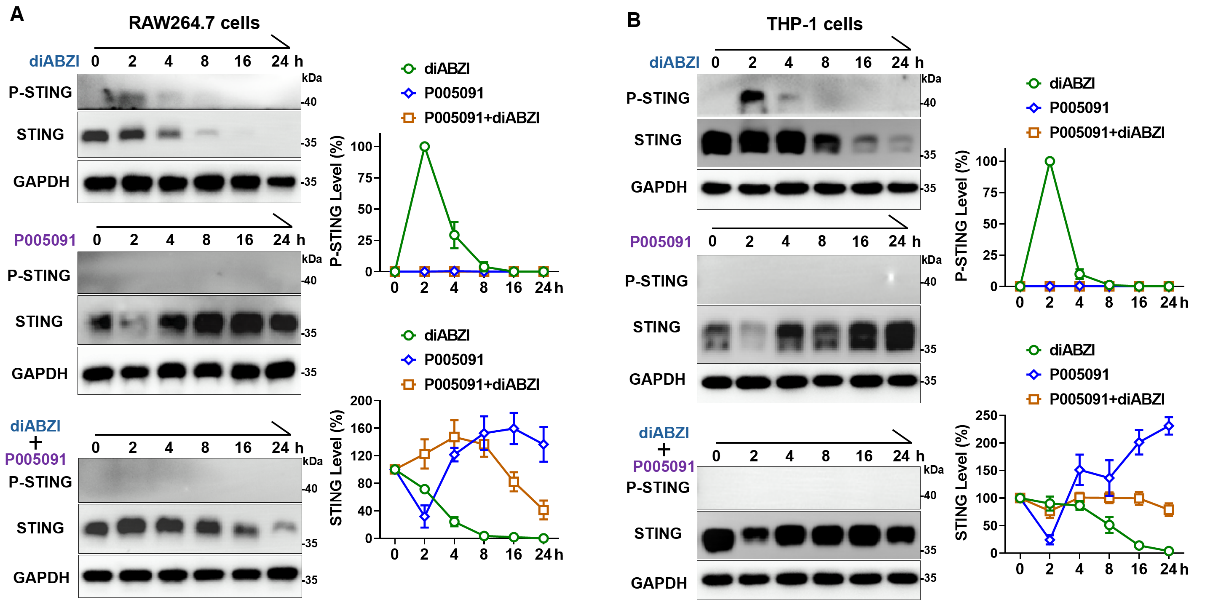


**Figure S17: Extended time course analysis of STING pathway inhibition by P005091.** (**A-B**) WB analysis of P005091-mediated suppression of diABZI (1 μM)-induced STING phosphorylation (0–24 hours) following 2 hours pre-incubation with P005091 in RAW264.7 cells (**A**) and THP-1 cells (**B**). Data were represented as mean ± S.D. (n = 3).

**Figure S18**


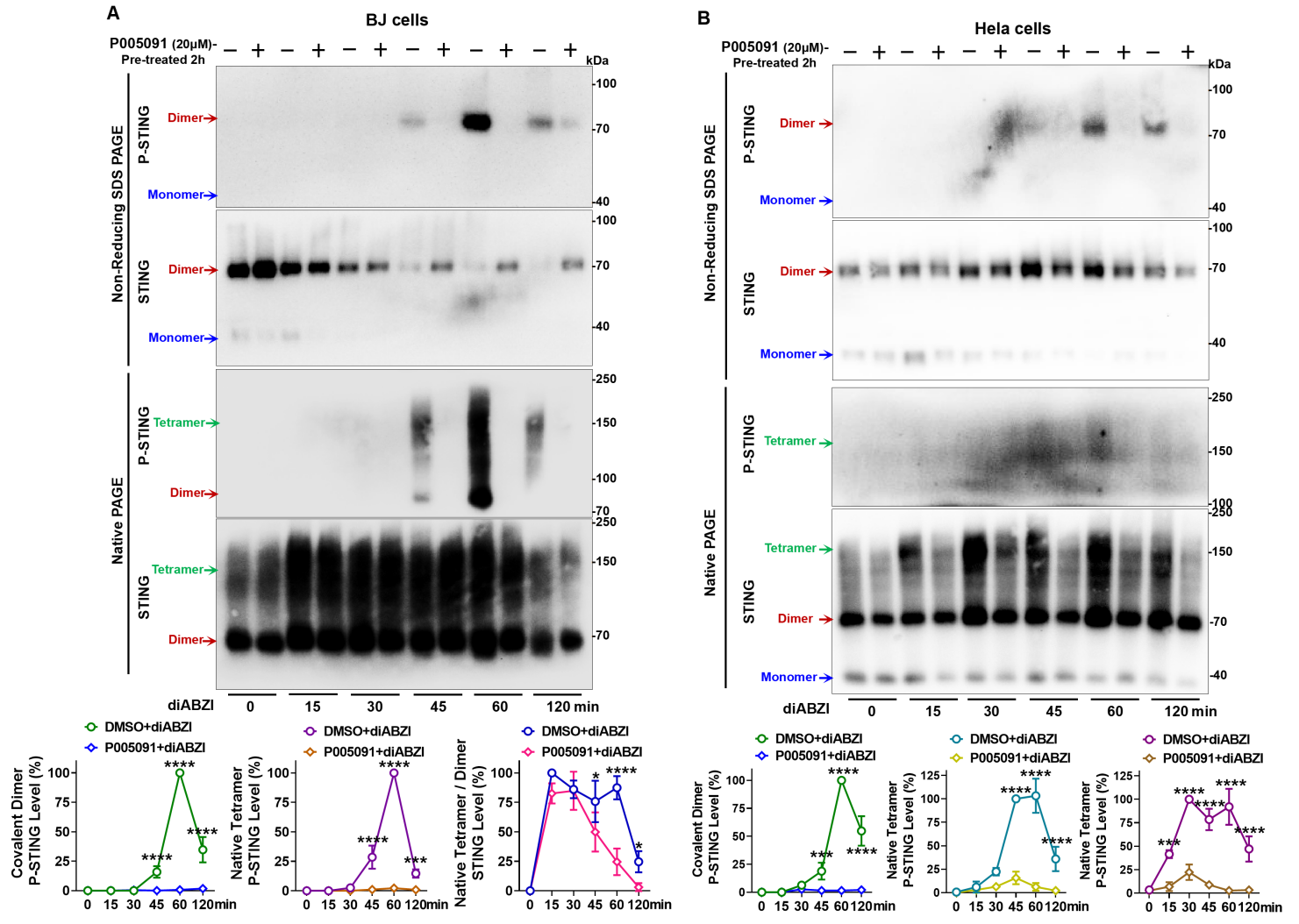


**Figure S18: Analysis of P005091-mediated inhibition of STING and P-STING dimerization and tetramerization.** Kinetic analysis of activation levels of STING induced by diABZI (1 μM) in BJ cells (**A**) and HeLa cells (**B**) was assessed by WB, including detection of dimer STING and dimer P-STING in Non-reducing SDS PAGE, tetramer STING and tetramer P-STING in Native PAGE. Quantiﬁcations of grayscale values data were represented as mean ± S.D. (n = 3). Statistical signiﬁcance was determined by two-way ANOVA (*, P < 0.05; ***, P < 0.001; ****, P < 0.0001).

**Figure S19**


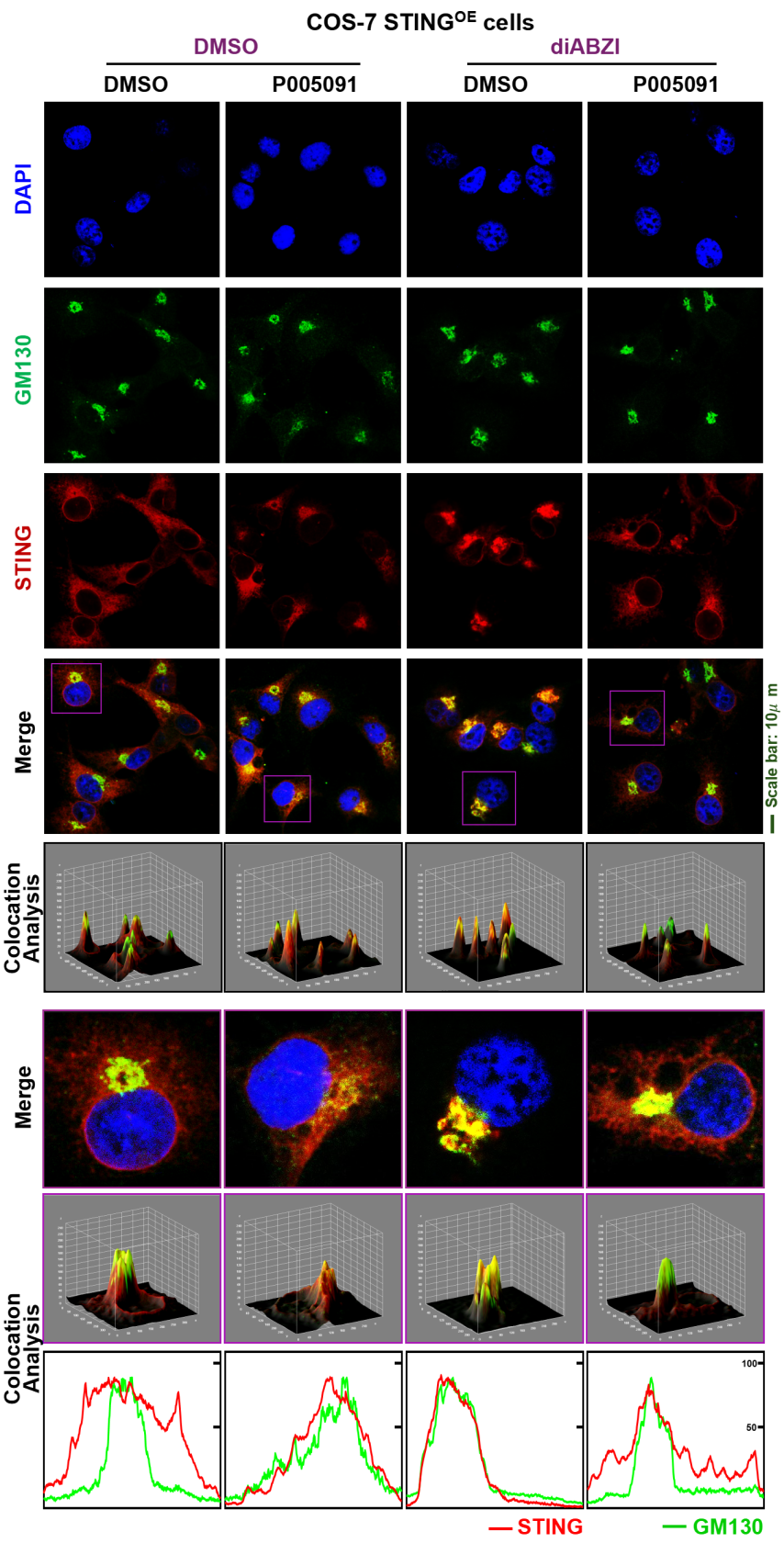


**Figure S19: IF assay captured the distribution of STING and GM130 in COS-7 cells.** Co-localization analysis demonstrates P005091 (20 μM) suppression of diABZI (1 μM)-induced STING translocation to the Golgi apparatus, data were represented from three independent biological replicates.

**Figure S20**


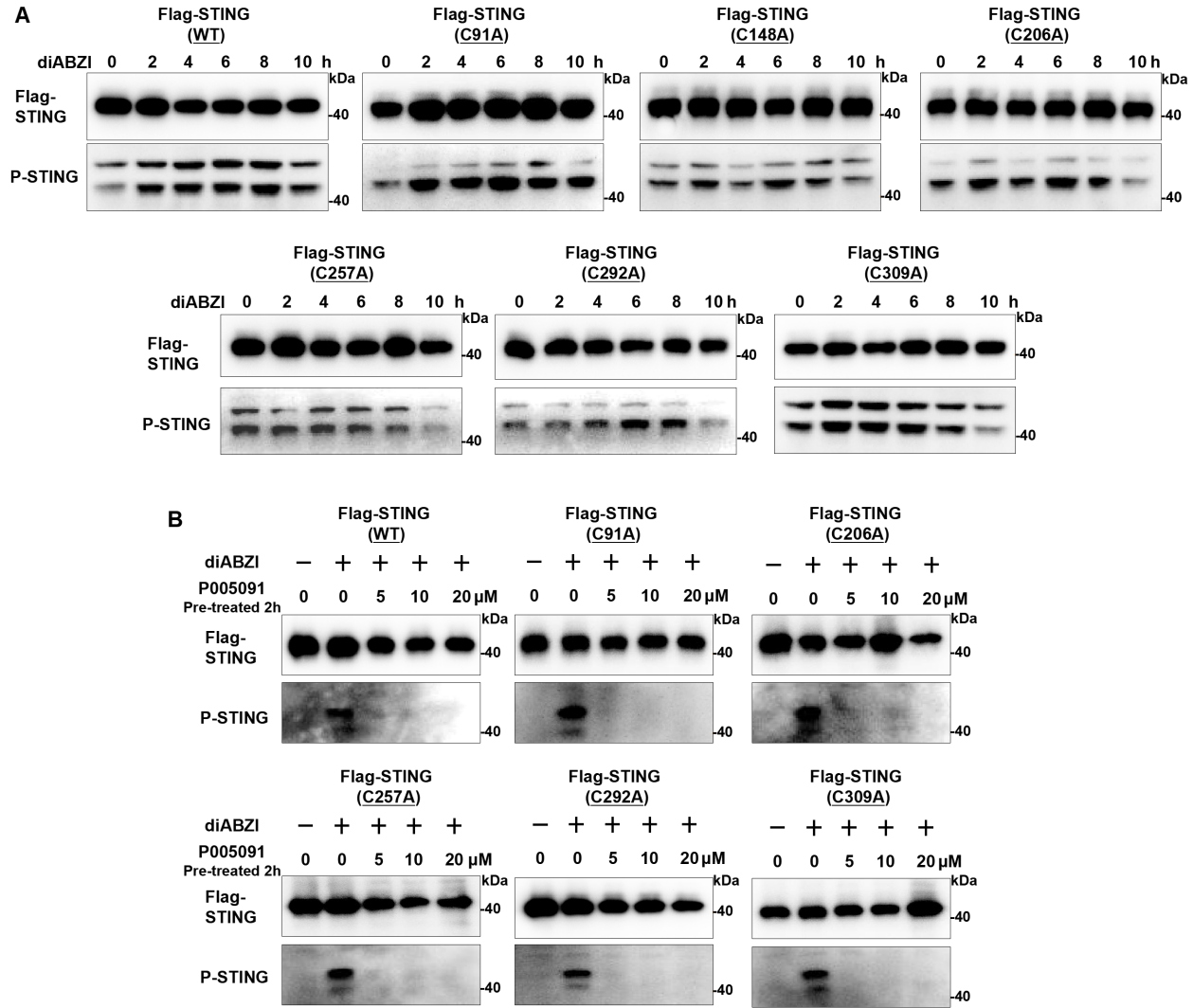


**Figure S20: Activation kinetics of STING single-point cysteine mutations and dose response analysis of P005091 Inhibition.** (**A**) WB analysis of time-dependent kinetic analysis of diABZI (5 μM)-induced activation of Flag-hSTING (WT, C91A, C148A, C206A, C257A, C292A, and C309A) in HEK293F cells. (**B**) WB analysis of dose-dependent inhibitory effects of P005091 (0-20 μM) on diABZI (5 μM, 2 hours)-activated Flag-hSTING (WT, C91A, C206A, C257A, C292A and C309A) in HEK293F cells.

**Figure S21**


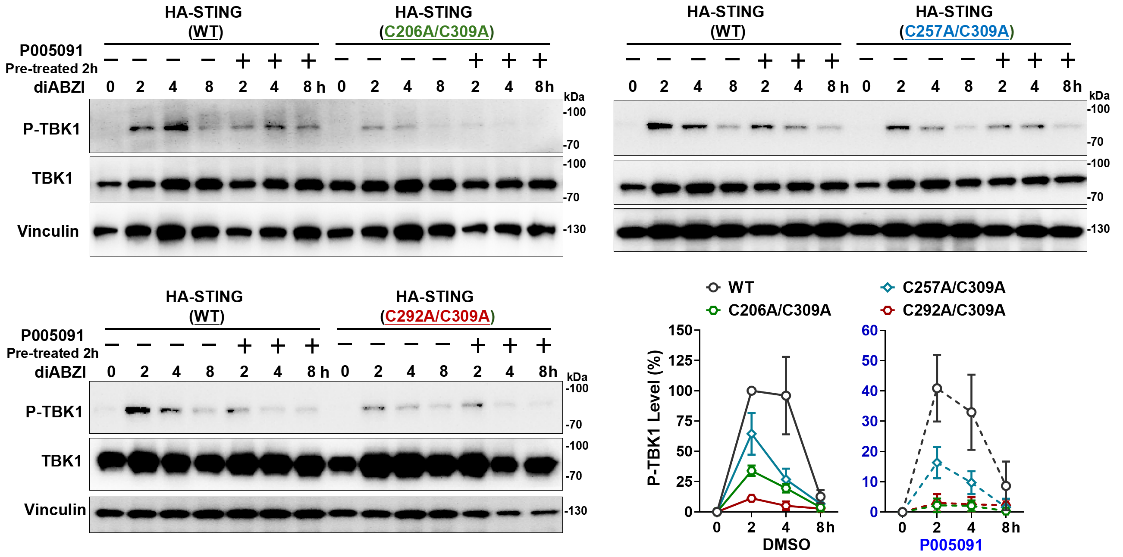


**Figure S21: Time-Course analysis of P005091-mediated suppression of TBK1 phosphorylation in STING cysteine double mutants.** WB analysis of P005091 inhibition on diABZI(5 μM)-induced TBK1 phosphorylation at the indicated time points (0-8 hours) in HEK293T cells. Cells were pre-incubated with P005091 (20 μM) for 2 hours before stimulation with diABZI across STING variants (WT, C206A/C309A, C257A/C309A and C292A/C309A). Data were represented as mean ± S.D. (n = 3).

**Figure S22**


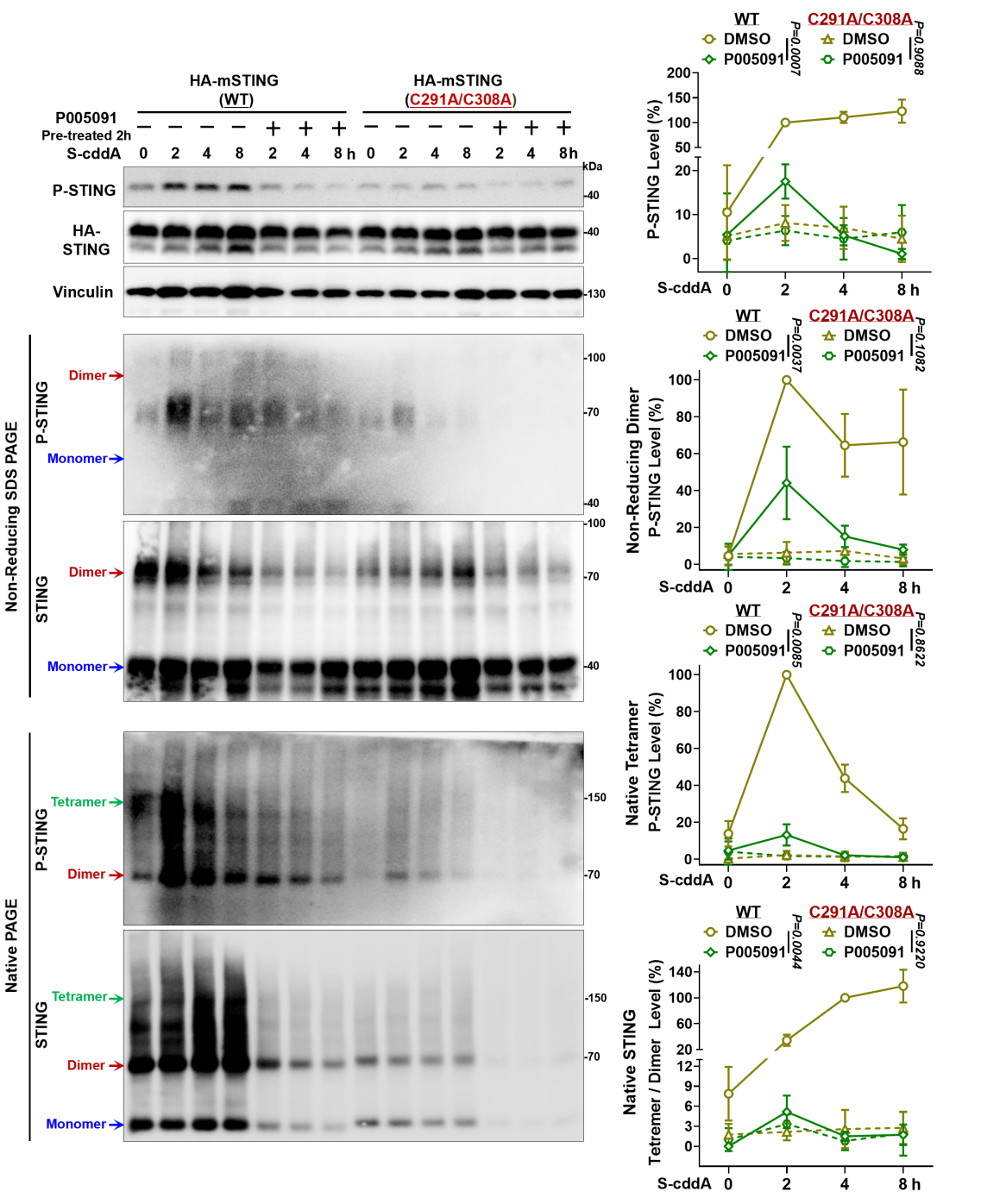


**Figure S22: Time-course analysis of P005091-mediated suppression of murine STING signaling.** Comprehensive analysis of diABZI (5 μM)-induced STING pathway activation in HEK293T cells reconstituted with murine STING (WT or C291A/C308A), evaluating modulation of monomer/dimer/tetramer states of STING and P-STING following 2 hours pre-incubation with P005091(20 μM) and stimulation at indicated time points. Data were represented as mean ± S.D. (n = 3). Statistical signiﬁcance was determined by two-way ANOVA (N.S., no signiﬁcance; P > 0.05; **, P < 0.01; ***, P < 0.001).

**Figure S23**


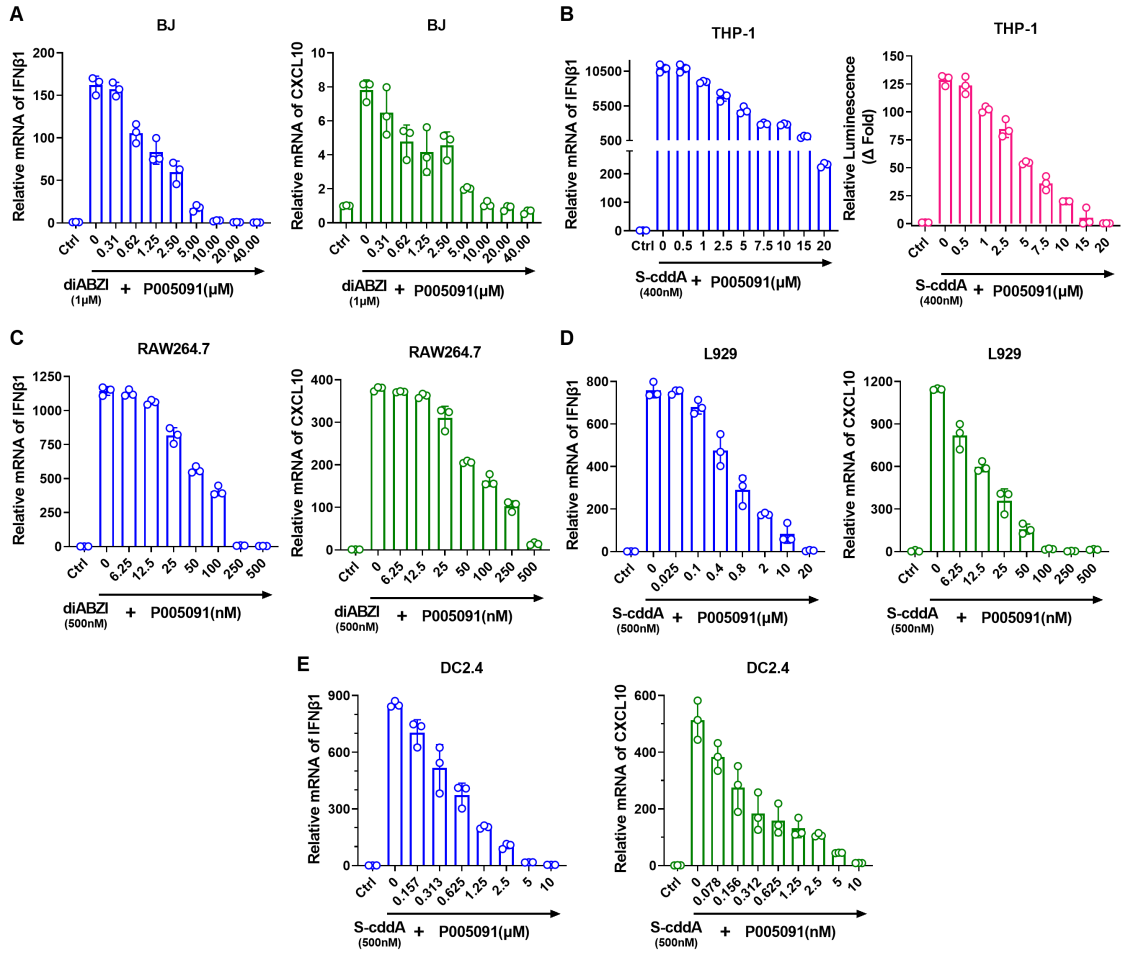


**Figure S23: Dose-response evaluation of the STING inhibitory activity of P005091.** (**A, C**) Dose-response analysis of P005091 inhibiting diABZI-induced IFN-β1 and CXCL10 responses in BJ and RAW264.7 cells, **related to Figure 4A, C**. (**B**) Dose-response analysis of P005091 inhibiting S-cddA-induced IFN-β1 and CXCL10 responses and ISG reporter gene expression (characterized by relative luminescence) in THP1-Lucia ISG cells, **related to Figure 4B**. (**D, E**) Dose-response analysis of P005091 inhibiting diABZI-induced IFN-β1 and CXCL10 responses in L929 and DC2.4 cells, **related to Figure 4D, E**. Data were represented as mean ± S.D. (n = 3).

**Figure S24**


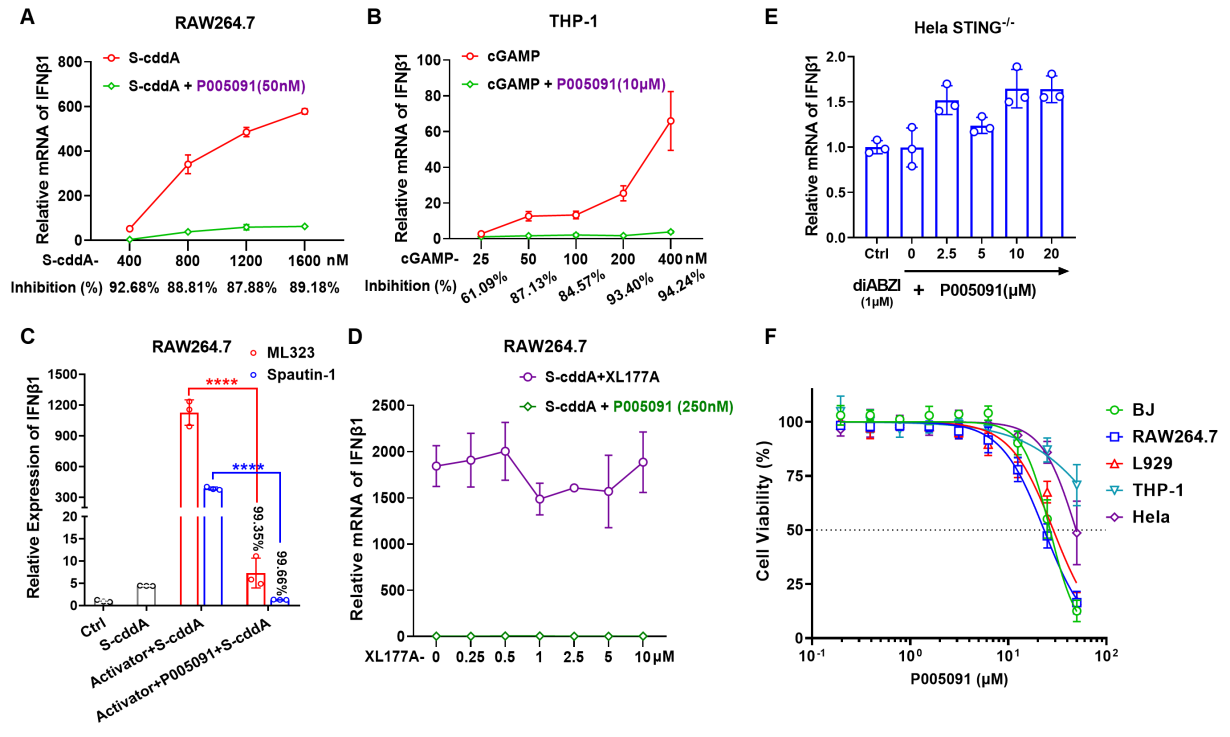


**Figure S24: P005091 inhibits potent STING pathway activation independently of USP7 inhibition.** (**A**) Inhibitory effect of P005091 (50 nM, pretreated for 2 hours) on IFN-β1 responses induced by S-cddA (400-1600 nM) in RAW264.7 cells. (**B**) Inhibitory effect of P005091 (10 μM, pretreated for 2 hours) on IFN-β1 responses induced by cGAMP (25-400 nM) in THP-1 cells. (**C**) Inhibitory efficacy of P005091 (5 μM) on IFN-β1 responses synergistically activated by ML323 (5 μM) or Spautin-1 (5 μM) combined with S-cddA (0.1 μM) for 2 hours in RAW264.7 cells. (**D**) Inhibitory effect of P005091 (250 nM) on IFN-β1 responses induced by XL177A across a range of concentrations in combination with S-cddA (500 nM) in RAW264.7 cells. (**E**) Inhibitory effect of P005091 (0-20 μM) on IFN-β1 responses induced by diABZI (1 μM) in HeLa-STING KO cells. (**F**) Cytotoxicity assessment of P005091 (0-50 μM) after 48-hour treatment on BJ, RAW264.7, L929, THP-1 and HeLa cells using the CCK-8 assay. Data were represented as mean ± S.D. (n = 3). Statistical signiﬁcance was determined by one-way ANOVA (****, P < 0.0001).

**Figure S25**


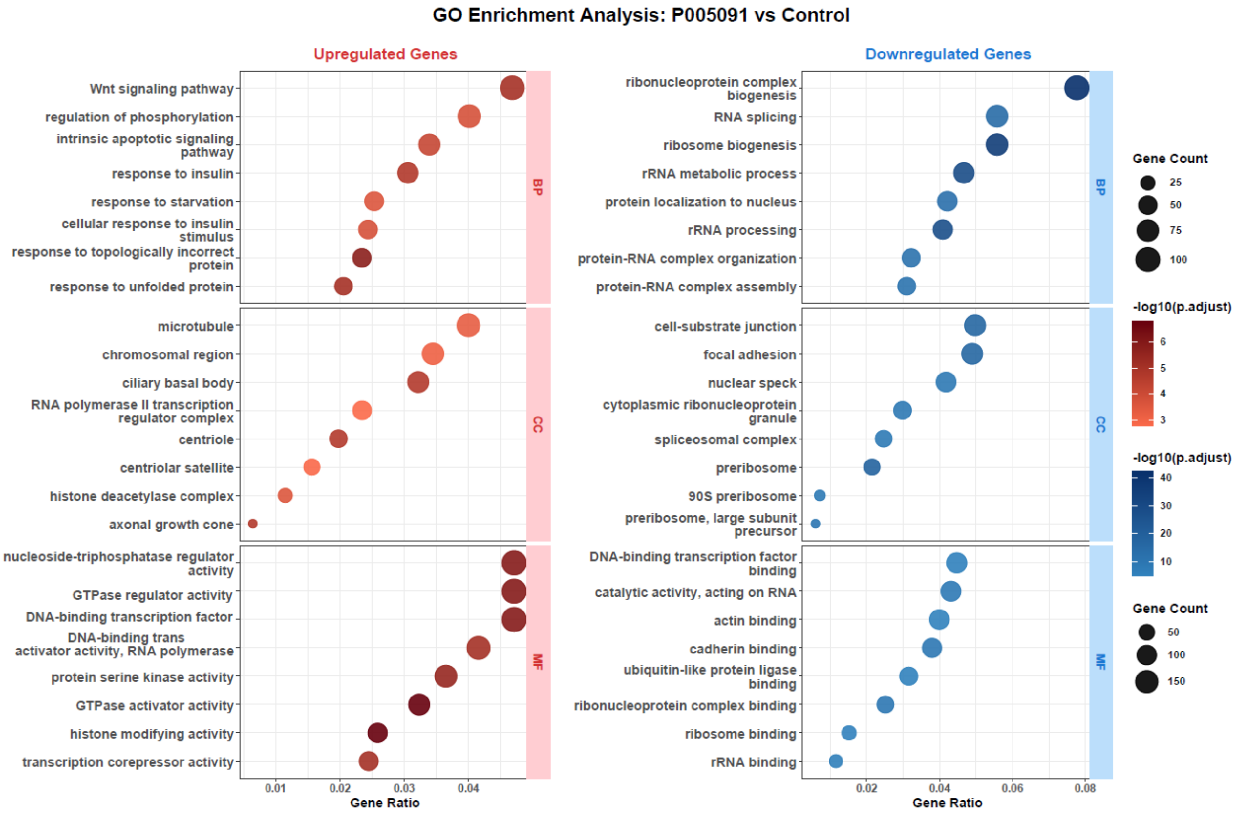


**Figure S25: GO enrichment analysis between the control group and the P005091 treatment group.** Differential gene expression between the Ctrl and P005091 treatment groups was visualized using a bubble plot.

**Figure S26**


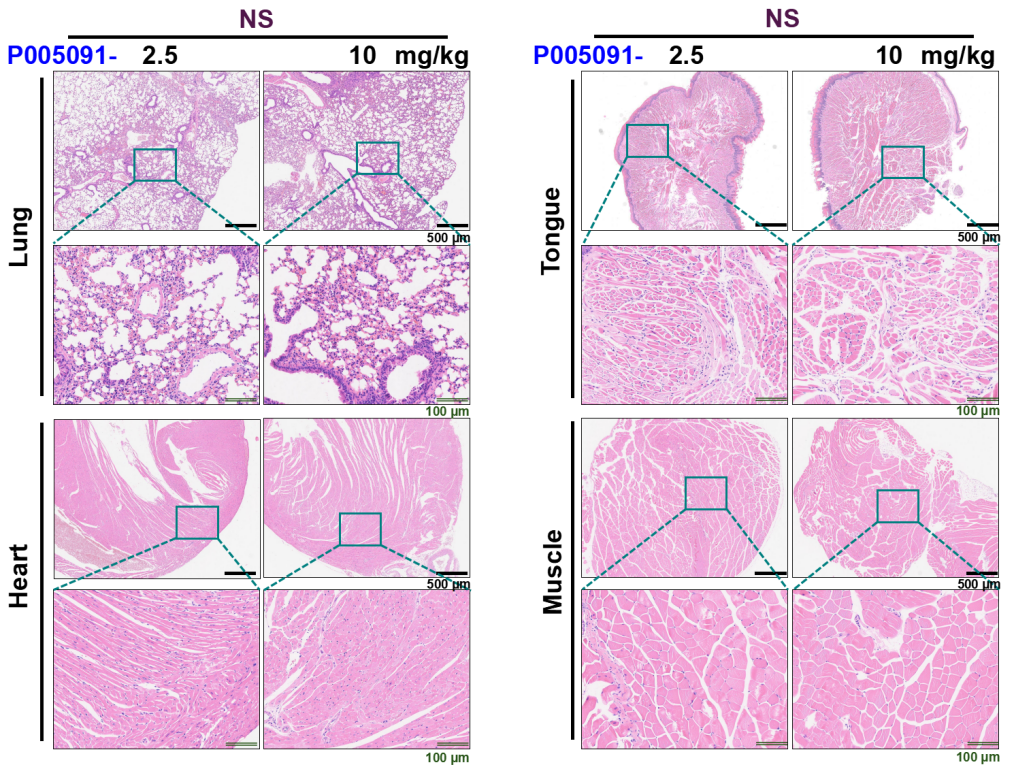


**Figure S26: Effect of P005091 on inflammatory phenotypes in naive mice.** H&E-stained sections of lung, tongue, heart and skeletal muscle tissues of mice from the saline-treated group administered P005091 (2.5 and 10 mg/kg).

**Figure S27**


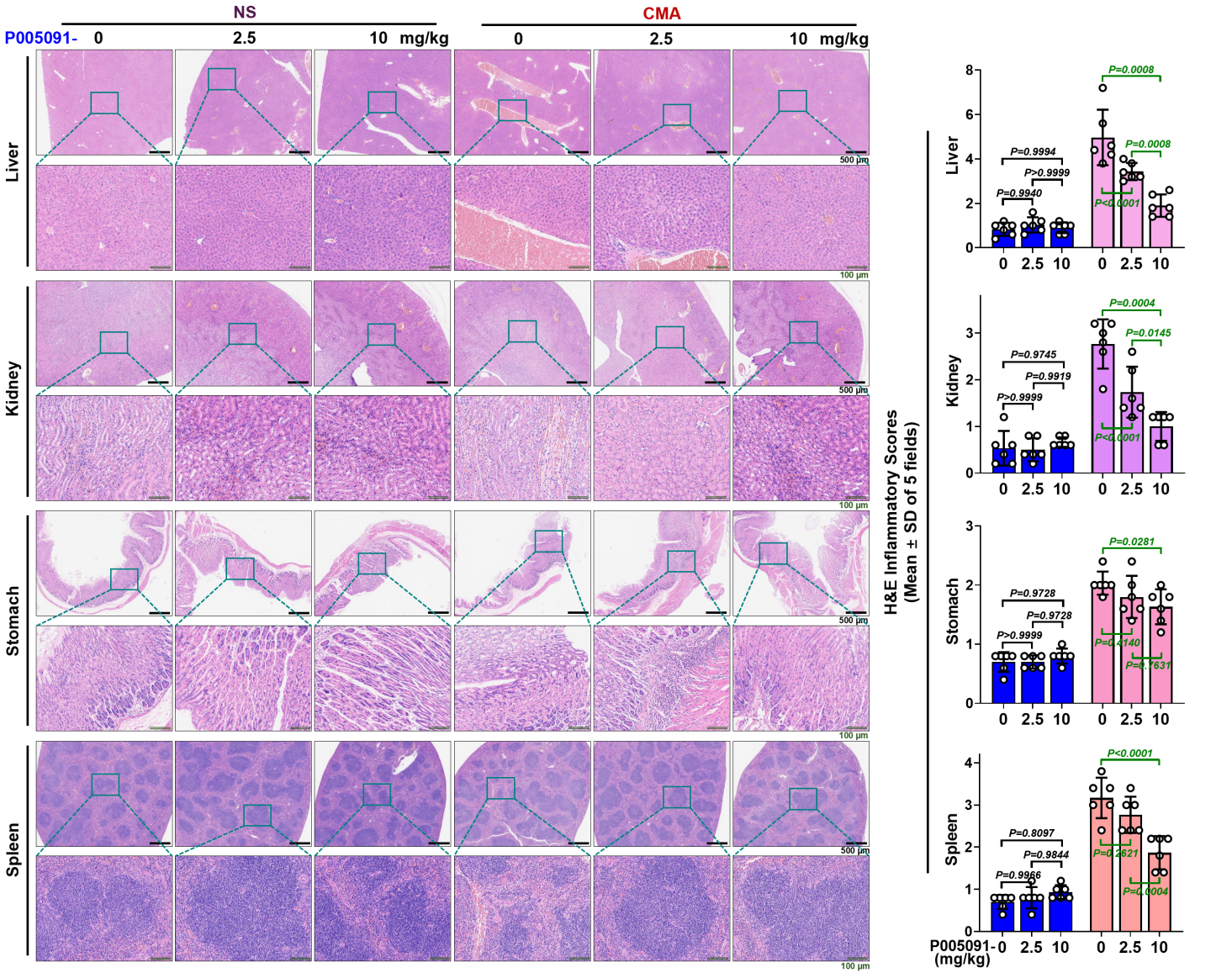


**Figure S27: Ameliorative effects of P005091 on CMA-induced inflammation.**Representative H&E-stained sections of liver, kidney, stomach, and spleen tissues from saline-treated controls and CMA-challenged mice administered P005091 (0, 2.5 and 10 mg/kg), with corresponding histological scores. Data were represented as mean ± S.D. (n = 6). Statistical signiﬁcance was determined by two-way ANOVA (N.S., P>0.05; *, P < 0.05; ***, P < 0.001; ****, P < 0.0001).

**Figure S28**


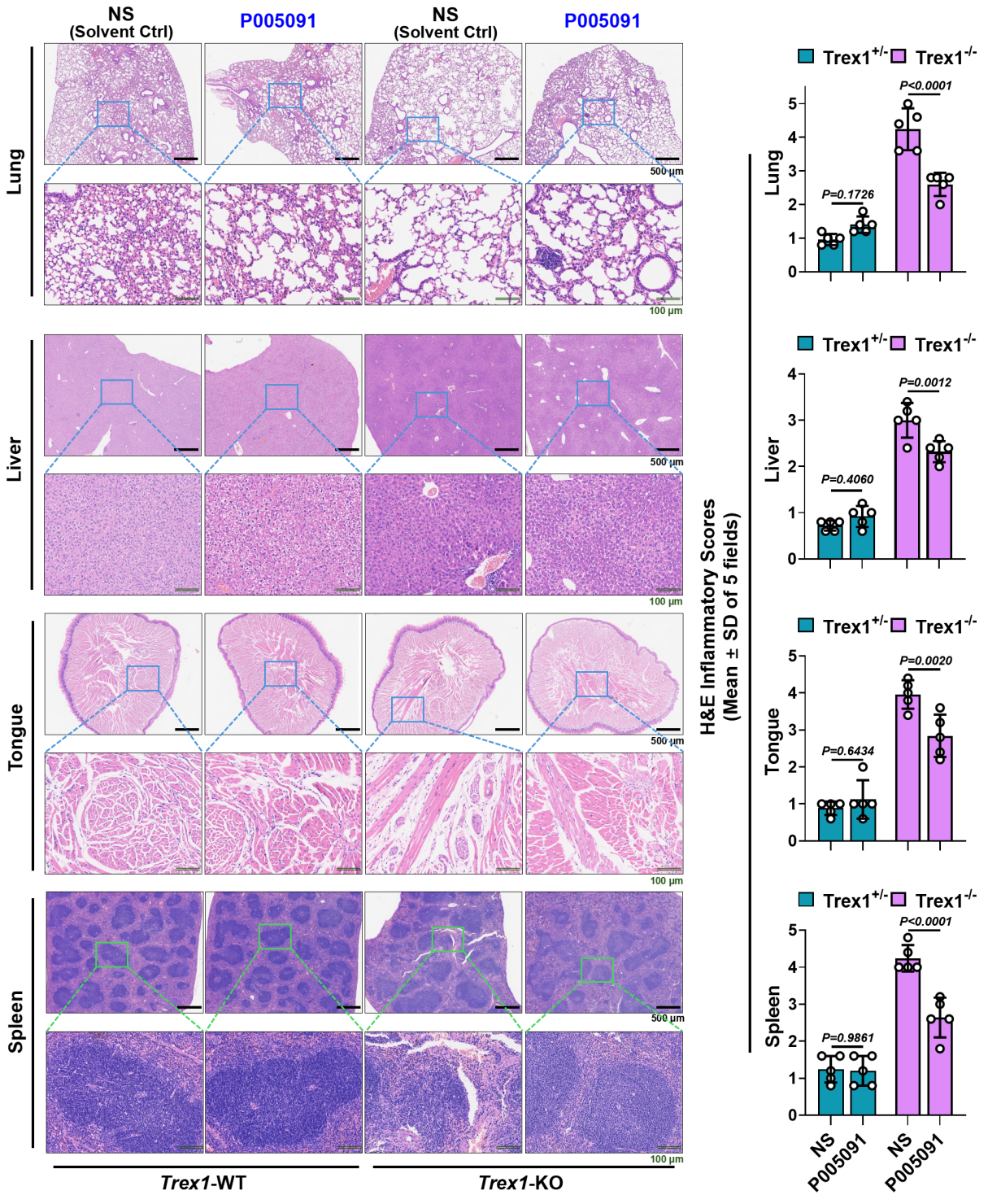


**Figure S28: Alleviation of inflammatory lesions by P005091 in *Trex1*-KO mice.**H&E-stained sections and corresponding histological scores of lung, liver, tongue and spleen tissues from saline or P005091 (5 mg/kg)-treated *Trex1*-WT and KO mice. Data were represented as mean ± S.D. (n = 5). Statistical signiﬁcance was determined by two-way ANOVA (N.S., P > 0.05; **, P < 0.01; ****, P < 0.0001).

**Figure S29**


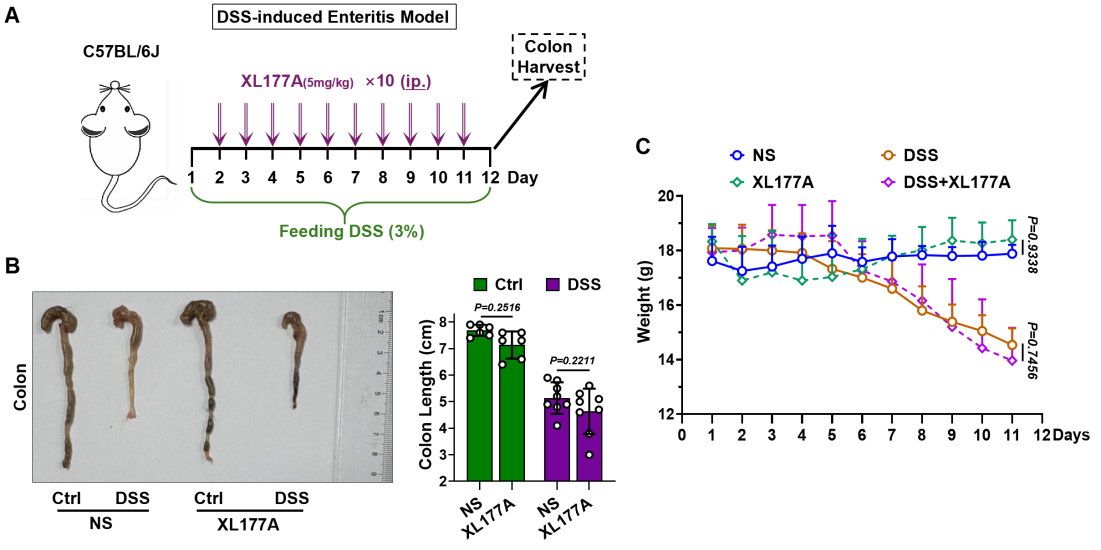


**Figure S29:** **Therapeutic efficacy of XL177A in DSS-induced acute inflammatory bowel disease.** (**A**) Schematic of DSS-induced colitis and XL177A dosing regimen. (**B**) Left: Representative photographic documentation of colons; Right: Quantification of colon length reduction. (**C**) Dynamic monitoring of body weight change throughout disease progression. Data were represented as mean ± S.D. (n = 6). Statistical signiﬁcance was determined by two-way ANOVA (N.S., P > 0.05).

**Figure S30**


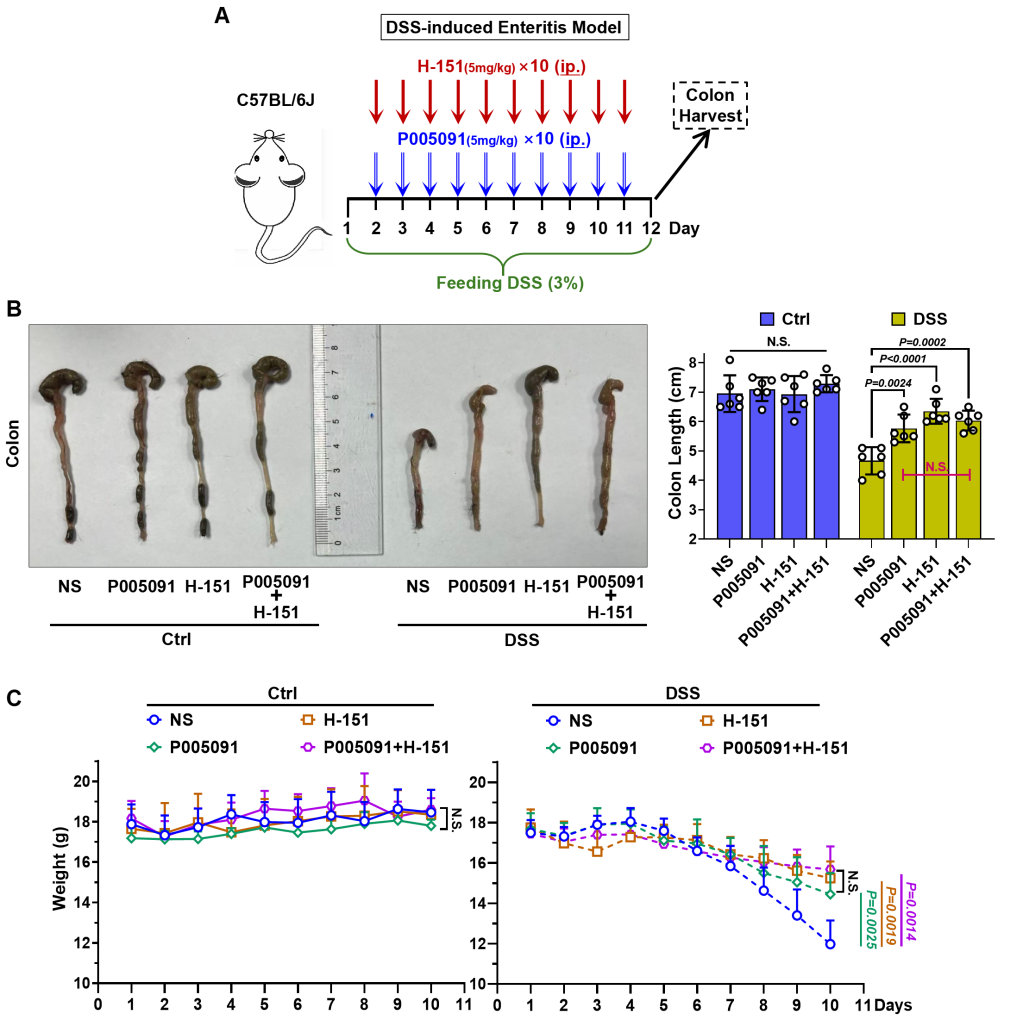


**Figure S30:** **Therapeutic effects of P005091 combined with H-151 on DSS-induced inflammatory bowel disease.** (**A**) Schematic of DSS-induced colitis and dosing regimens for P005091 and H-151. (**B**) Left: Representative photographic documentation of colons; Right: Quantification of colon length reduction. (**C**) Dynamic monitoring of body weight change throughout disease progression. Data were represented as mean ± S.D. (n = 6). Statistical signiﬁcance was determined by two-way ANOVA (N.S., P > 0.05; **, P < 0.01; ***, P < 0.001; ****, P < 0.0001).

**Figure S31**


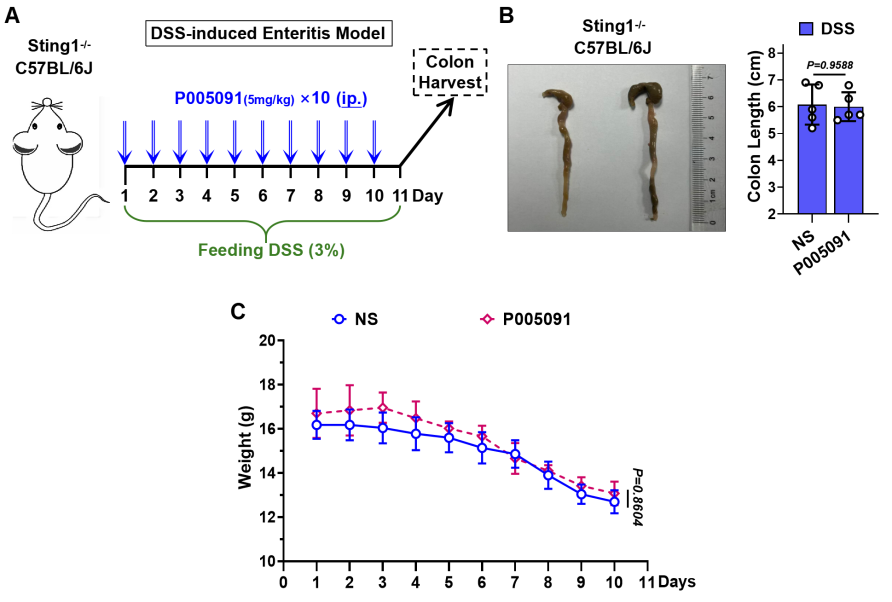


**Figure S31**: **Therapeutic effects of P005091 against DSS-induced inflammatory bowel disease in STING-deficient mice.** (**A**) Schematic of DSS-induced colitis and dosing regimens for P005091. (**B**) Left: Representative photographic documentation of colons; Right: Quantification of colon length. (**C**) Dynamic monitoring of body weight change throughout disease progression. Data were represented as mean ± S.D. (n = 5). Statistical signiﬁcance was determined by two-way ANOVA (N.S., P > 0.05).

**Figure S32**


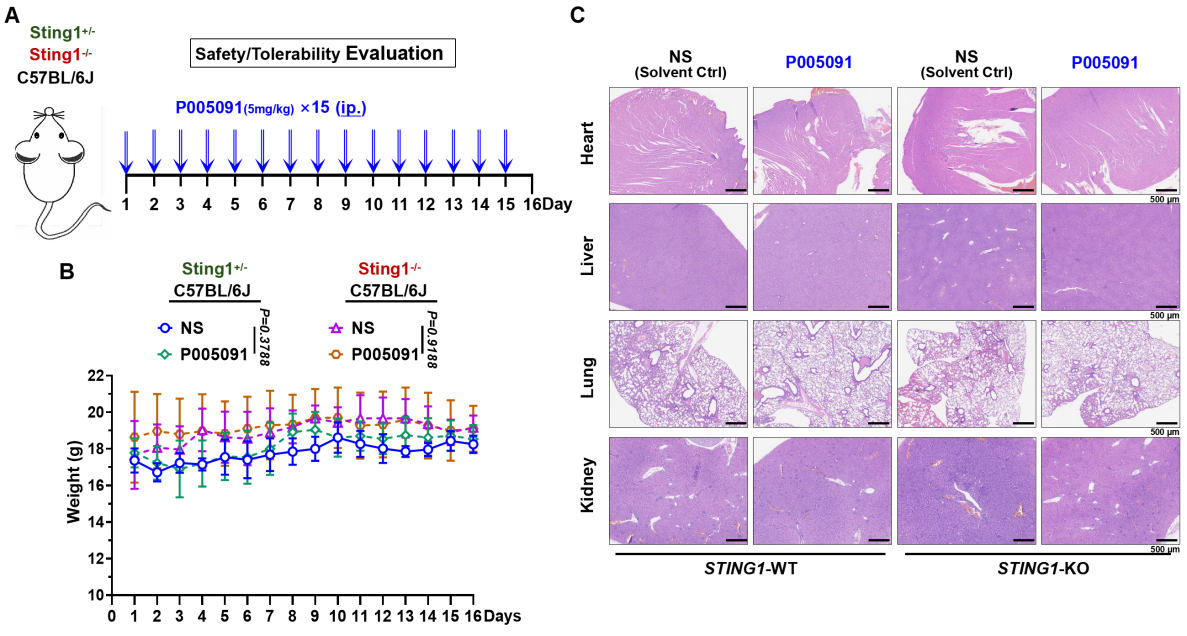


**Figure S32:** **Long-term toxicity assessment of P005091 in wild-type and STING-deficient mice.** (**A**) Schematic of P005091 dosing regimen. (**B**) Body weight monitoring of STING-WT and STING-deficiency mice. (**C**) H&E staining of heart, liver, lung and kidney of STING-WT and STING-deficient mice. Data were represented as mean ± S.D. (n = 3). Statistical signiﬁcance was determined by two-way ANOVA (N.S., P > 0.05).

**Figure S33**


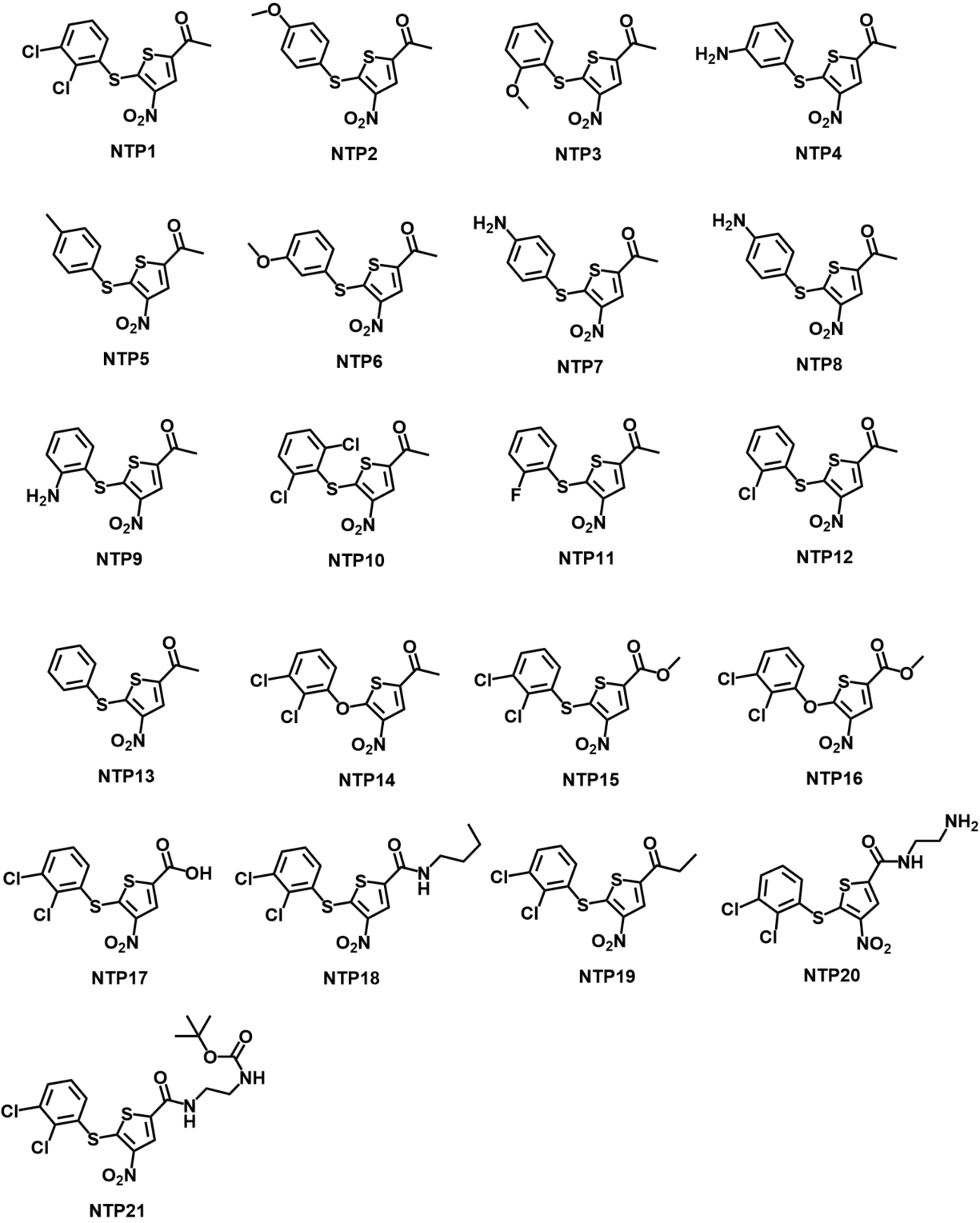


**Figure S33**: Chemical structure of NTP1-NTP21, **related to Figure 7A.**

**Figure S34**


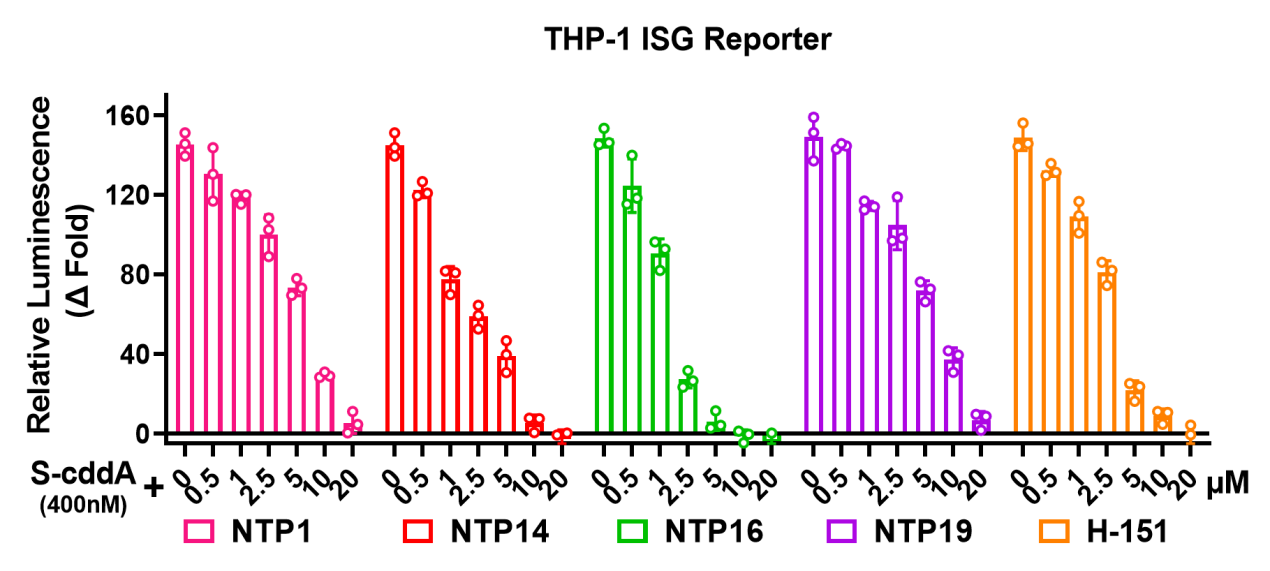


**Figure S34: Dose-response evaluation of structurally analog compounds for inhibiting ISG reporter gene expression.** Dose-response analysis of NTP1, NTP14, NTP16, NTP19 and H-151 on S-cddA-induced interferon-stimulated gene reporter expression in THP-1 Lucia ISG cells, **related to Figure 7B.** Data were represented as mean ± S.D. (n = 3).

**Figure S35**


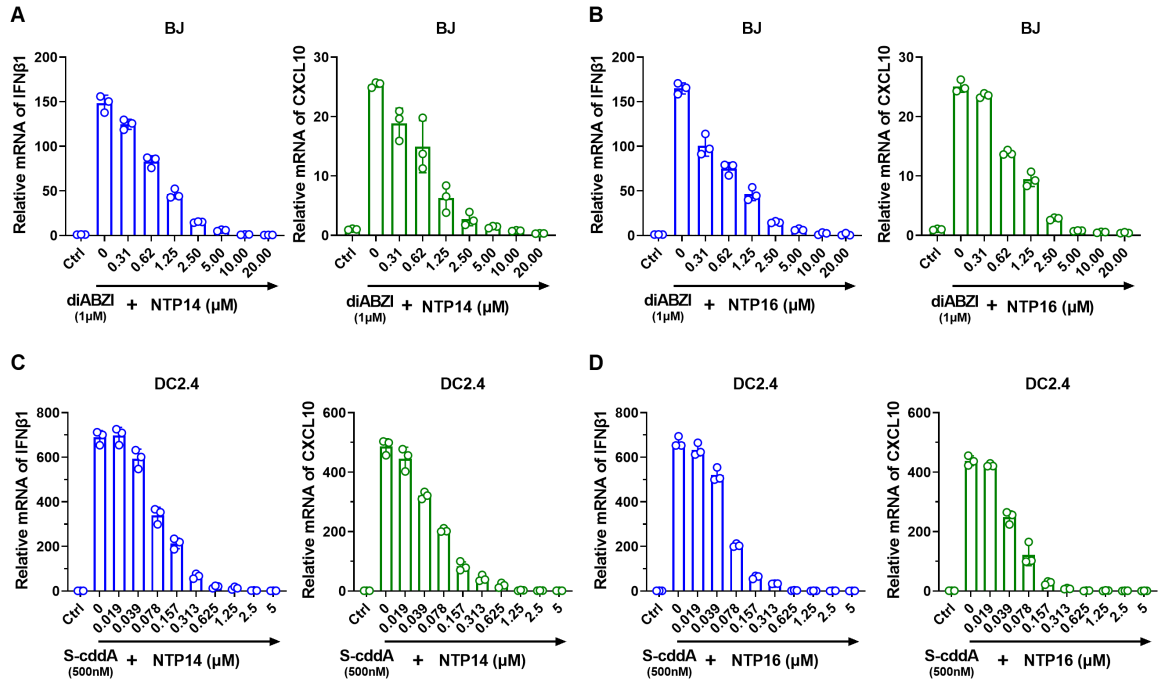


**Figure S35: Evaluation of STING inhibitory activity of NTP14 and NTP16.** Dose-response analysis of NTP14 (**A, C**) and NTP16 (**B, D**) inhibiting diABZI and S-cddA-induced IFN-β1 and CXCL10 responses in BJ cells (**A, B**) and DC2.4 cells (**C, D**). Data were represented as mean ± S.D. (n = 3).

**Figure S36**


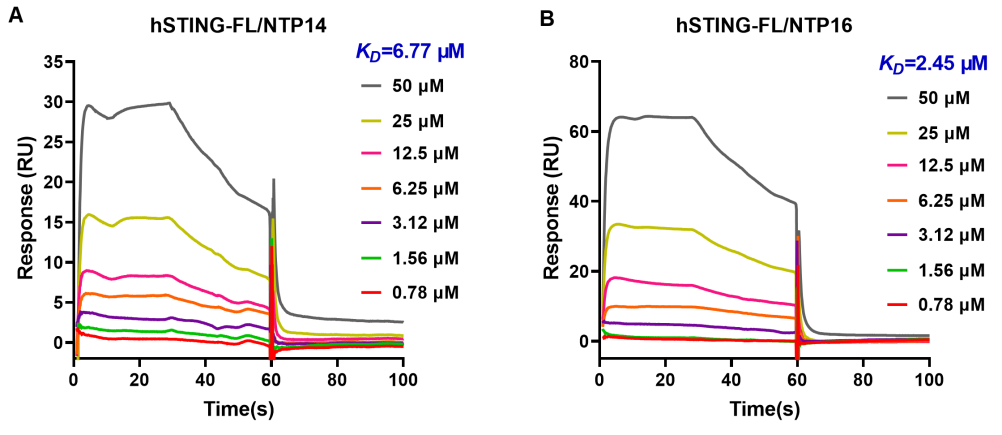


**Figure S36: Binding analysis of NTP14 and NTP16 with STING. (A-B)** SPR binding assays of STING-FL protein with NTP14 (**A**) and NTP16 (**B**) (0.78-50 μM). *K_D_* values were calculated using GraphPad Prism 8 software.

**Figure S37**


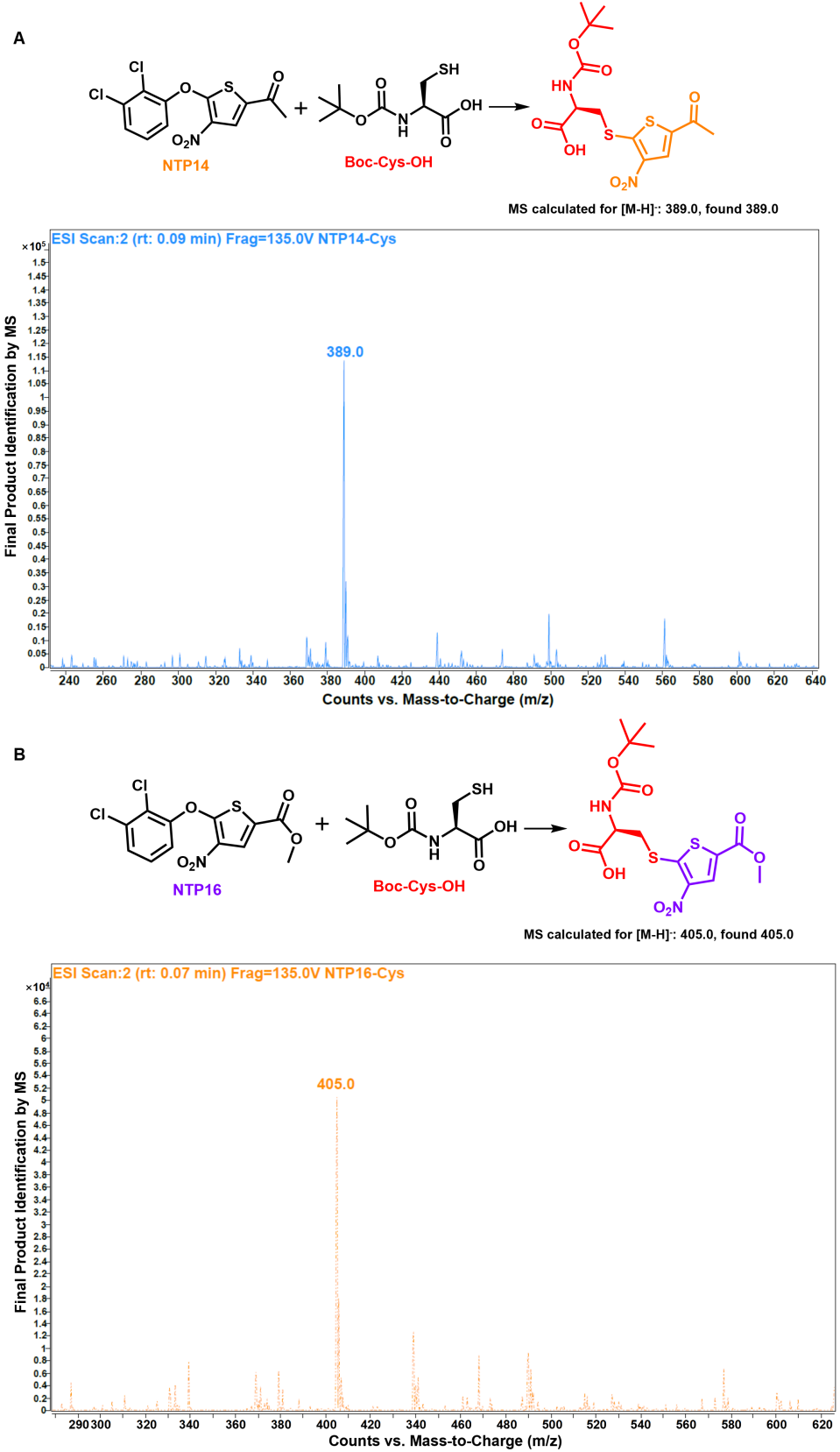


**Figure S37: Structural characterization of reaction products between NTP14/16 and Boc-Cys-OH.** (**A-B**) Nucleophilic substitution reactions of NTP14 (**A**) or NTP16 (**B**) (dissolved in DMF) with Boc-Cys-OH mediated by potassium carbonate. Purified products were structurally characterized by mass spectrometry.

**Figure S38**


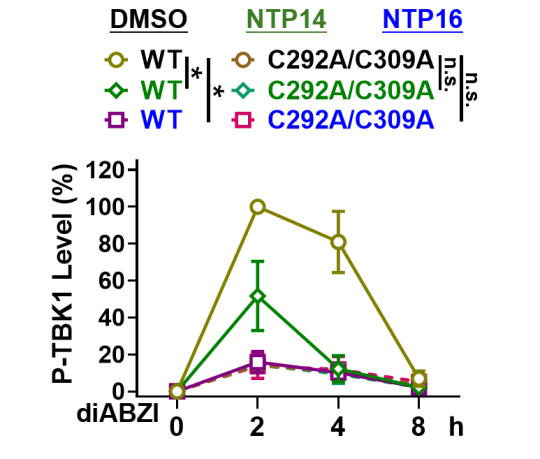


**Figure S38: Inhibitory effects of NTP14 and NTP16 on TBK1 phosphorylation.** HEK293T cells were transfected with STING (WT or C292A/C309A) plasmids. Phosphorylation activation of TBK1 by diABZI (5 μM) at the indicated time points and its inhibition by P005091 were quantified via grayscale analysis. Data were represented as mean ± S.D. (n = 3), **related to Figure 7E.** Statistical signiﬁcance was determined by two-way ANOVA (N.S., P > 0.05; *, P < 0.05).

**Figure S39**


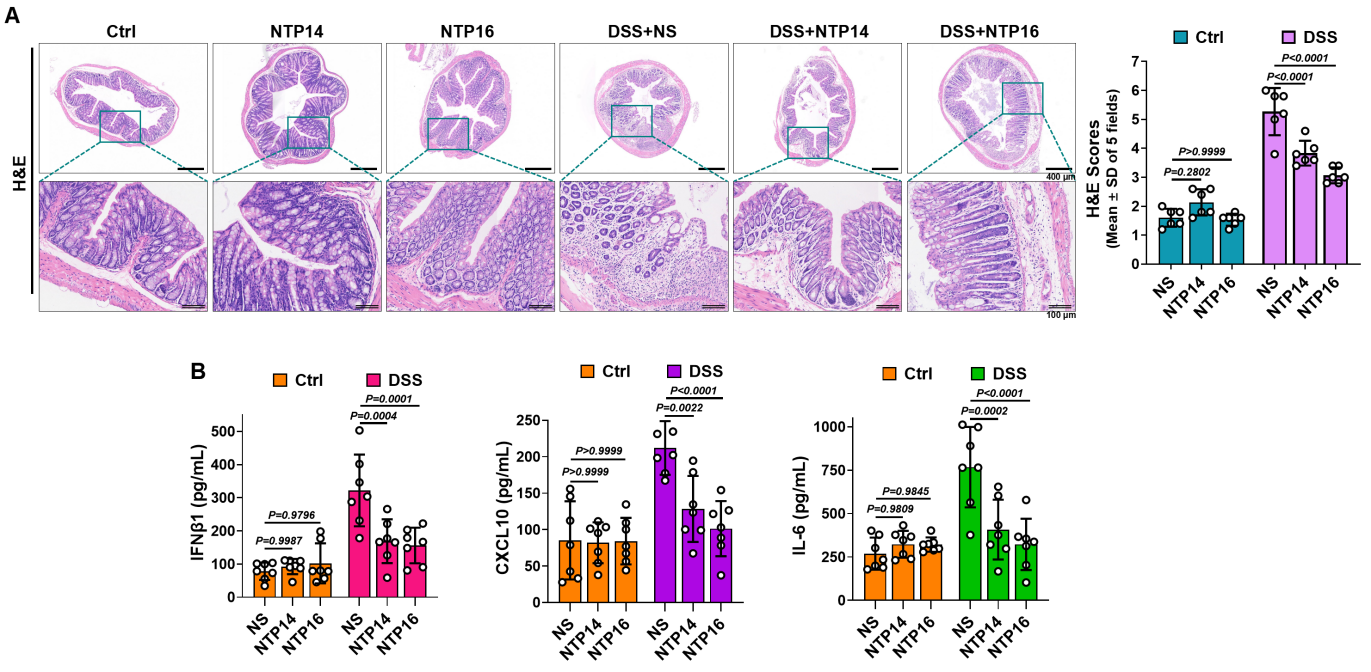


**Figure S39: NTP14 and NTP16 inhibit DSS-induced inflammatory phenotypes in mice.** (**A**) Histopathological analysis and scoring of colon tissues of mice. (**B**) Serum levels of IFN-β1, CXCL10, and IL-6 treated with NTP14 or NTP16 (0 and 5 mg/kg) in DSS-treated groups and water control groups. Data were represented as mean ± S.D. (n ≥ 6). Statistical signiﬁcance was determined by two-way ANOVA (N.S., P > 0.05; **, P < 0.01; ***, P < 0.001; ****, P < 0.0001).

**Figure S40**


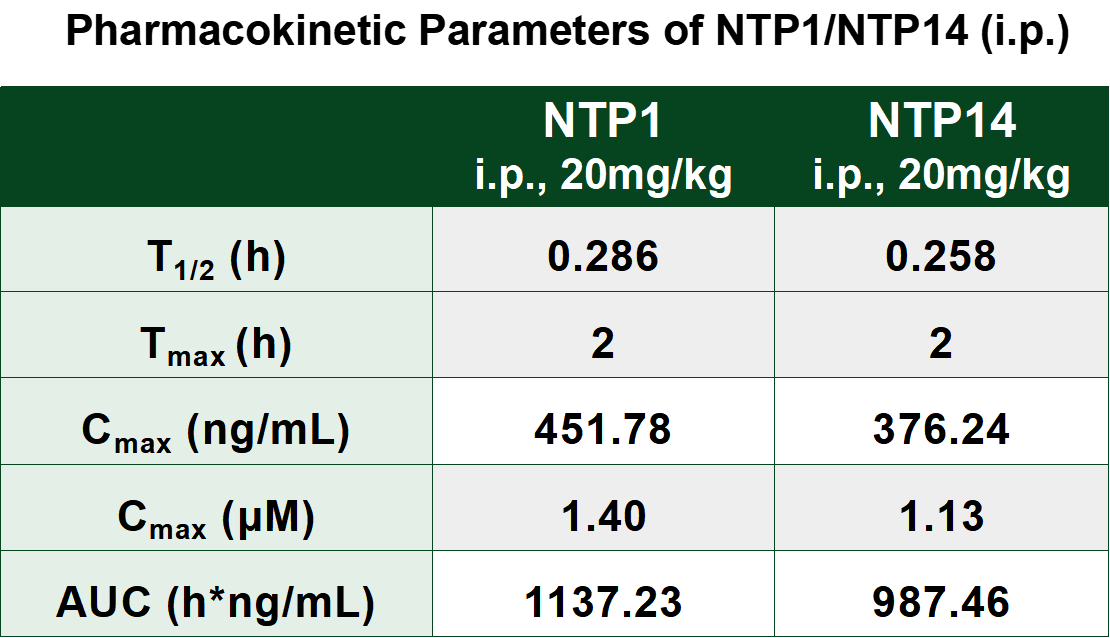


**Figure S40: Pharmacokinetic evaluation of NTP1 and NTP14.** SD rats received intraperitoneal administration of NTP1 or NTP14 (20 mg/kg). Plasma samples were collected at 0-24 hours post administration, with drug concentrations quantified by LC-MS. Pharmacokinetic parameters (T_1/2_, T_max_, C_max_, AUC) were calculated using Phoenix WinNonlin software.

**Table S1: Information on compounds for LC/MS based screening**

| **Name** | **CAS Number** | **MolWt** | **Target** |
| --- | --- | --- | --- |
| **Oritinib** | 2035089-28-0 | 539.67 | EGFR |
| **BTK inhibitor 17** | 1858206-76-4 | 456.5 | BTK |
| **EN6** | 1808714-73-9 | 368.34 | Proton pump; Autophagy |
| **Olafertinib** | 1660963-42-7 | 530.57 | EGFR |
| **BLU9931** | 1538604-68-0 | 509.38 | FGFR |
| **Z-VAD(OMe)-FMK** | 187389-52-2 | 467.49 | Caspase |
| **TED-347** | 2378626-29-8 | 313.7 | YAP |
| **Naquotinib** | 1448232-80-1 | 562.71 | EGFR |
| **Lipoic acid** | 1200-22-2 | 206.33 | Endogenous Metabolite; Mitochondrial Metabolism; Reactive Oxygen Species |
| **Ibrutinib** | 936563-96-1 | 440.5 | BTK; Src; Ligands for Target Protein for PROTAC; Tyrosine Kinases |
| **Ritlecitinib** | 1792180-81-4 | 285.34 | JAK |
| **Fisogatinib** | 1707289-21-1 | 503.38 | FGFR |
| **WZ-3146** | 1214265-56-1 | 464.95 | EGFR |
| **Z-VAD-FMK** | 161401-82-7 | 453.46 | Caspase |
| **Halobetasol propionate** | 66852-54-8 | 484.96 | Phospholipase |
| **K-Ras G12C-IN-4** | 2376328-55-9 | 561.07 | Kras |
| **Alflutinib** | 1869057-83-9 | 568.59 | Cytochromes P450;EGFR |
| **H3B-6527** | 1702259-66-2 | 629.54 | FGFR |
| **TL-895** | 1415823-49-2 | 447.5 | BTK |
| **evobrutinib** | 1415823-73-2 | 429.51 | BTK |
| **Mobocertinib** | 1847461-43-1 | 585.7 | EGFR |
| **Allitinib tosylate** | 1050500-29-2 | 621.08 | EGFR;FLT |
| **AZ-5104** | 1421373-98-9 | 485.58 | EGFR |
| **YKL-5-124** | 1957203-01-8 | 515.61 | CDK |
| **Borussertib** | 1800070-77-2 | 596.68 | Akt |
| **VLX1570** | 1431280-51-1 | 469.39 | DUB |
| **3CAI** | 28755-03-5 | 193.63 | Akt |
| **Z-FA-FMK** | 197855-65-5 | 386.42 | Cysteine Protease; SARS-CoV |
| **ARS-853** | 1629268-00-3 | 432.94 | Raf; Apoptosis; Ras |
| **Almonertinib** | 1899921-05-1 | 525.64 | EGFR |
| **JAK3-IN-6** | 1443235-95-7 | 350.37 | JAK |
| **M2N12** | 2376577-06-7 | 393.83 | Phosphatase |
| **DHODH-IN-4** | 1148125-93-2 | 347.2 | Dehydrogenase |
| **CHMFL-EGFR-202** | 2089381-40-6 | 489.96 | EGFR |
| **WZ8040** | 1214265-57-2 | 481.01 | EGFR |
| **FGFR2-IN-3** | 2549174-42-5 | 509.53 | FGFR |
| **YAP/TAZ inhibitor-2** | 2762617-31-0 | 390.33 | YAP |
| **EGFR-IN-9** | 1226549-39-8 | 476.53 | EGFR |
| **JAK3i** | 1918238-72-8 | 354.34 | JAK |
| **A 21960** | 609-15-4 | 164.59 | Anti-infection |
| **BJJF078** | 2531244-56-9 | 523.6 | Glutaminase |
| **Pantethine** | 16816-67-4 | 554.72 | Endogenous Metabolite |
| **GSK-3β inhibitor 3** | 1448990-73-5 | 327.37 | Apoptosis;GSK-3 |
| **TPCK** | 402-71-1 | 351.85 | Proteasome |
| **BMX-IN-1** | 1431525-23-3 | 524.59 | BTK |
| **MRTX-1257** | 2206736-04-9 | 565.71 | Kras |
| **CC-90003** | 1621999-82-3 | 458.44 | ERK |
| **AV-412** | 451493-31-5 | 851.41 | EGFR |
| **Clobetasol propionate** | 25122-46-7 | 466.97 | Cytochromes P450; Glucocorticoid Receptor |
| **RSL3** | 1219810-16-8 | 440.88 | Glutathione Peroxidase; Ferroptosis; GPX4 |
| **K-Ras(G12C) inhibitor 12** | 1469337-95-8 | 449.67 | Apoptosis; Raf; Ras |
| **CNX-2006** | 1375465-09-0 | 545.53 | EGFR |
| **DTNB** | 69-78-3 | 396.35 | Others |
| **(±)-Zanubrutinib** | 1633350-06-7 | 471.55 | BTK |
| **Ac-YVAD-CMK** | 178603-78-6 | 540.99 | IL Receptor; Caspase; Immunology/Inflammation related; Pyroptosis |
| **Mavelertinib** | 1776112-90-3 | 415.42 | EGFR |
| **Spebrutinib** | 1202757-89-8 | 423.44 | Src; BTK |
| **EN523** | 2094893-05-5 | 234.25 | OTUB1 |
| **Ac-FLTD-CMK** | 2376255-48-8 | 569.05 | Caspase |
| **Btk inhibitor 2** | 1558036-85-3 | 431.49 | BTK |
| **EN4** | 1197824-15-9 | 416.47 | c-Myc |
| **BPK-29 hydrochloride** | 2444815-73-8 | 506.46 | KEAP1 |
| **K-975** | 2563855-03-6 | 287.74 | TEAD |
| **MALT1 inhibitor MI-2** | 1047953-91-2 | 455.72 | MALT |
| **PF-6274484** | 1035638-91-5 | 372.78 | EGFR |
| **Lazertinib** | 1903008-80-9 | 554.64 | EGFR |
| **NQ301** | 130089-98-4 | 325.75 | Thrombin; Platelet aggregation |
| **KC7F2** | 927822-86-4 | 570.38 | HIF; HIF/HIF Prolyl-Hydroxylase |
| **FIIN-4** | 2093088-81-2 | 634.73 | FGFR |
| **BOC-D-FMK** | 634911-80-1 | 263.26 | Caspase; Apoptosis |
| **Olmutinib** | 1802181-20-9 | 486.59 | EGFR |
| **Mometasone furoate** | 83919-23-7 | 521.43 | Glucocorticoid Receptor |
| **FM-381** | 2226521-65-7 | 428.49 | JAK |
| **Halcinonide** | 3093-35-4 | 454.96 | Smo; Glucocorticoid Receptor |
| **JH-X-119-01** | 2227368-54-7 | 452.46 | IRAK |
| **Z-DEVD-FMK** | 210344-95-9 | 668.66 | Caspase |
| **Atuzabrutinib** | 1581714-49-9 | 539.6 | BTK |
| **Mutant EGFR inhibitor** | 1421373-62-7 | 520.03 | EGFR |
| **iRGD peptide** | 1392278-76-0 | 948.04 | Integrin |
| **Degrasyn** | 856243-80-6 | 384.27 | Bcr-Abl; Apoptosis; JAK; DUB; Autophagy |
| **Alogliptin Benzoate** | 850649-62-6 | 461.51 | DPP-4; Ferroptosis; Proteasome |
| **Anagliptin** | 739366-20-2 | 383.45 | Proteasome; DPP-4 |
| **VLX1570** | 1431280-51-1 | 469.39 | DUB |
| **Saxagliptin hydrate** | 945667-22-1 | 333.43 | Proteasome; DPP-4 |
| **DBPR108** | 1186426-66-3 | 324.39 | Proteasome; DPP-4 |
| **Brensocatib** | 1802148-05-5 | 420.46 | Proteasome |
| **TPCK** | 402-71-1 | 351.85 | Proteasome |
| **18α-Glycyrrhetinic acid** | 1449-05-4 | 470.68 | Apoptosis; NF-κB; Proteasome |
| **SJ-172550** | 431979-47-4 | 428.87 | E1/E2/E3 Enzyme; Mdm2 |
| **LDN-91946** | 439946-22-2 | 314.32 | DUB |
| **Parthenolide** | 20554-84-1 | 248.32 | Mitophagy; Apoptosis; NF-κB; Autophagy; HDAC |
| **PYZD-4409** | 423148-78-1 | 351.67 | E1/E2/E3 Enzyme |
| **P005091** | 882257-11-6 | 348.22 | DUB |
| **NSC59984** | 803647-40-7 | 265.27 | p53 |
| **Heclin** | 890605-54-6 | 283.32 | E1/E2/E3 Enzyme |
| **DKM 2-93** | 65836-72-8 | 243.69 | E1/E2/E3 Enzyme |
| **Alogliptin** | 850649-61-5 | 339.39 | DPP-4; Proteasome; Ferroptosis |
| **RA375** | 2649154-57-2 | 588.99 | Proteasome |
| **EOAI3402143** | 1699750-95-2 | 503.42 | DUB |
| **PYR-41** | 418805-02-4 | 371.3 | E1/E2/E3 Enzyme; Apoptosis |

**Supplementary Methods**

**Compound Screening**

For each compound, a stock solution (2 mM) was added to the N-sumo-6His-STING (Novoprotein, China) protein to achieve a final concentration of 40 μM. The mixture was incubated at 4 °C for 24 hours on a rotary shaker. The following LC-MS/MS analysis and data processing of the protein samples were performed by Wininnovate Bio (Shenzhen, China). Protein samples (50 μg) were denatured by adding 8 M urea. Subsequently, 10 mM dithiothreitol (DTT) was added, and the mixture was incubated at room temperature for 2 hours to achieve reduction. Following reduction, 50 mM iodoacetamide (IAA) was introduced, and alkylation was allowed to proceed at room temperature for 15 minutes in the dark. The alkylated samples were then subjected to enzymatic digestion using trypsin (1 μg) and incubated overnight at 37°C. The resulting peptide mixtures were desalted using C18 solid-phase extraction (SPE) cartridges. Briefly, samples were loaded onto pre-activated C18 cartridges, washed with 0.1% (v/v) trifluoroacetic acid (TFA) in water, and eluted with 80% (v/v) acetonitrile containing 0.1% TFA. The eluates were lyophilized and reconstituted in 0.1% (v/v) formic acid. Protein mass spectrometry was performed on an Orbitrap Fusion Lumos (ThermoFisher, USA) connected to an EASY-nLC1200 system, equipped with a PepMap C18 2 μm 75 μm x 25 mm column. Buffer A was 0.1% formic acid in water, and buffer B was 0.1% formic acid in 50% acetonitrile. The analytical gradient was from 5% to 95% buffer B within 6.0 min with 0.75 mL min^−1^ flow. Mass spectra were collected from 100 – 3000 Da and the spectra were deconvoluted using the software Peaks Studio 12.

**Full-length STING Protein Expression and Purification**

The coding sequence of human full-length STING was cloned into the pCAGGS vector with a carboxy-terminal 6His-Flag tag. Expi293F cells (Thermo Fisher Scientific) were maintained in Expi293 medium (Thermo Fisher Scientific) at 37 ℃ with 5% CO_2_. When cell density reached 2 × 10^6^ cells/mL, 2 mg expression plasmids were pre-mixed with 6 mg PEI (Polysciences) in the fresh culture medium and incubated for 20 min, and the mixture was then added to 1 L of cell culture. After 24 hours post-transfection, 10 mM sodium butyrate was added to the culture medium. Cells were collected 48 hours after infection for STING purification.

Thawed cell pellets were resuspended in hypotonic lysis buffer (containing 25 mM Hepes pH7.4, 20 mM KCl, 10mM MgCl_2_, Cocktail (ROCHE), 1mM PMSF, 10μg/ml Dnase I) for 30 min and disrupted by Dounce homogenizer for 160 strokes. The lysates were centrifuged for 10 min at 3,000 ×g to remove nuclei and unlysed cells. The membrane fraction was pelleted by centrifugation at 150,000 ×*g* for 1 hour.

The membrane pellets were solubilized in solubilization buffer (20 mM HEPES pH 7.4, 150 mM NaCl, 1% DDM, 0.2% CHS, protease inhibitor cocktail (Roche), 1 mM PMSF) using a Dounce homogenizer, using a volume proportional to the original culture volume (e.g., per liter). The suspension was rotated at 4°C for 2 hours, followed by centrifugation at 150,000 ×*g* for 40 minutes to remove insoluble material. Anti-flag M1 affinity resin was added to the solubilized receptor and rotated for 2 hours at 4℃, then washed with a buffer containing 20 mM Hepes pH7.4, 150 mM NaCl, 0.05% DDM,0.005% CHS. The receptor was subsequently eluted with the same buffer supplemented with flag peptide (0.3 mg/ml). The elution was concentrated and then loaded onto Superose 6 Increase column (GE Healthcare) with a running buffer of 20 mM Hepes pH 7.5, 150 mM NaCl, 0.05% DDM, 0.005% CHS. The peak fraction of STING was collected, concentrated, and stored at −80℃ until use. The above protein expression and purification experiments were carried out by Readcrtstal Biotechnology Co., Ltd (Suzhou, China).

**Surface Plasmon Resonance (SPR) Assay**

SPR binding assays were conducted on a Biacore T200 instrument with the GxP Package with running buffer HBS-EP+ (Cytiva, Switzerland). STING-CTD or STING-FL protein was immobilized onto a CM7 sensor chip by amine coupling. A concentration gradient of P005091, NTP14 and NTP16 (0.39-25 μM or 0.78-50 μM) were injected over the sensor surface at a flow rate of 30 μL/min for kinetic analysis. The association phase was maintained for 60 seconds, followed by a dissociation period of 180 seconds. The dissociation constant (*K_D_*) was calculated using GraphPad Prism 8 software.

**Differential Scanning Fluorimetry (DSF) Assay**

DSF assay mixtures were prepared using the PROTEOSTAT^®^ Thermal Shift Stability Assay Kit (Enzo Life Sciences, USA) with the following components: 3 µL of STING-CTD protein (2.4 µg) or STING-FL protein (1.8 µg), 18.5 µL of 1× Assay Buffer (diluted with ddH₂O), 1 µL of P0005091 or LB244 (3.125-50 µM dissolved in DMSO), and 1 µL of PROTEOSTAT^®^ TS Detection Reagent (reconstituted in 10× Assay Buffer). The prepared reaction mixtures were loaded into a PCR instrument and subjected to a thermal denaturation protocol increasing from 30 to 99 °C at a rate of 0.05 °C/s. Fluorescence intensity was recorded at 0.3-second intervals during the temperature ramp. The melting temperature (Tm) of the protein samples was determined using GraphPad Prism 8 software.

**Glutathione Binding Stability Assay**

P005091 (5 mM) and glutathione (5 mM) were mixed in 1 mM PBS buffer (containing 25% DMSO and 100 mM NaCl) and the reaction system was incubated at 37°C. Aliquots of the reaction mixture were collected at 2 h, 4 h, and 12 h for HPLC analysis using a mobile phase gradient of 5%-100% acetonitrile. The relative content of P005091 was calculated by comparing its peak area to that of a P005091 standard (5 mM) using the area normalization method.

**Cell Thermal Shift Assay**

RAW264.7 cells (1 × 10⁷ cells per group) were seeded into 15 cm cell culture dishes. P005091 or LB244 was added to the respective cell cultures at a final concentration of 0.5 μM, while control groups received an equivalent volume of DMSO. Following incubation for 2 hours, the culture medium was discarded. Cells were washed once with PBS buffer and subsequently detached with trypsin. The resulting cell suspensions were pelleted by centrifugation at 1,500 rpm for 3 minutes, washed with PBS buffer, and centrifuged again. The supernatant was discarded, and the cell pellets were resuspended in PBS containing protease and phosphatase inhibitors and 0.1% NP-40. Each resuspended sample was aliquoted into 7 equal portions for heating at different temperatures. Individual aliquots were subjected to heating at predetermined temperatures for 3 minutes. Cell lysis was subsequently achieved by performing 5 cycles of freezing in liquid nitrogen and thawing in a 25 °C water bath. Lysates were centrifuged at 13,000 rpm for 10 minutes at 4 °C. The supernatant was collected, mixed with 5×SDS-PAGE loading buffer, and heated at 95 °C for 10 minutes.

**Analysis of USP7 Protein Covalent Modification**

Samples were prepared by co-incubating P005091(40 μM) with the USP7 protein (MedChemExpress, HY-P74478) at 37°C for 3 hours. Subsequent covalent modification analysis was performed on the samples by Wininnovate Bio (Shenzhen, China) as the same methods for STING-CTD mentioned in manuscript.

**Immunofluorescence Analysis**

COS7-HASTING cells (2 × 10⁴ cells per dish) were seeded in confocal dishes. After cell attachment, the experimental groups were pretreated with 10 μM P005091 for 2 hours, followed by stimulation with 1 μM diABZI for another 2 hours. After treatment, the culture medium was removed, and cells were fixed with 4% paraformaldehyde at room temperature for 15 minutes. After washing with PBS, cells were permeabilized with 0.1% Triton X-100 for 20 minutes. Subsequently, samples were blocked with QuickBlock™ Immunostaining Blocking Buffer (Beyotime, China) at room temperature for 1 hour. After removal of the blocking solution, cells were washed three times with PBS. Primary antibodies (against STING and GM130) diluted 1:100 were applied and incubated overnight at 4°C. After thorough washing with PBS, the cells were incubated with corresponding fluorescently labeled secondary antibodies for 1 hour at room temperature in the dark. Finally, nuclei were counterstained with DAPI (Beyotime, China) for 5 minutes. Images were acquired using an Olympus FV3000 confocal laser scanning microscope. Colocalization analysis was performed on fluorescence images using ImageJ software.

**RNA Isolation and RT-qPCR Analysis**

BJ/ RAW264.7/L929/DC2.4 or HeLa-STING KO cells were seeded in 6-well plates at a density of 1.0 × 10⁶ cells per well and allowed to adhere, while THP-1 cells were seeded in suspension. After an appropriate incubation period, cells were pretreated with various concentrations of P005091 (0-40 μM) for 2 hours. Subsequently, the cells were stimulated for 2 hours with specified concentrations of cGAMP (1 μM for RAW264.7 cells, 25 μM for THP-1 cells), S-cddA (0.5 μM for RAW264.7 and L929 cells, 0.4 μM for THP-1 cells), DMXAA (20 μM), diABZI (1 μM for BJ and HeLa-STING KO cells, 10 μM for RAW264.7 cells), MSA-2 (40 μM), LPS (10 μg/ml), and poly (I:C) (5 μg/ml).

Total RNA was extracted using the Total RNA Rapid Extraction Kit (GOONIE, China), and RNA concentration was measured with a Nanodrop 2000 (Thermo Fisher Scientific). cDNA was synthesized from 1 µg of RNA using HiScript Q RT SuperMix (with gDNA wiper). Quantitative real-time PCR (RT-qPCR) was performed on a Bio-Rad CFX96™ system using ChamQ SYBR Color qPCR Master Mix. Each experimental group included at least three technical replicates. Relative gene expression was calculated using Bio-Rad CFX Manager software, and further statistical analysis was conducted with GraphPad Prism 8.

The primer sequences used were as follows: Mouse *Gapdh*: Forward: 5′‐ATTCAACGGCACAGTCAAGG‐3′, Reverse: 5′‐GCAGAAGGGGCGGAGATGA‐3′. Mouse *Ifnβ1*: Forward: 5′‐GGTGGAATGAGACTATTGTTG‐3′, Reverse: 5′‐AAGTGGAGAGCAGTTGAG‐3′. Mouse *Cxcl10*: Forward: 5′‐CCAAGTGCTGCCGTCATTTT‐3′, Reverse: 5′‐TTCATCGTGGCAATGATCTCAAC‐3′. Mouse *Il-6*: Forward: GGGACTGATGCTGGTGACAA, Reverse: CGCACTAGGTTTGCCGAGTA. Mouse *Isg15*: Forward: TGGTACAGAACTGCAGCGAG, Reverse: CAGCCAGAACTGGTCTTCGT. Human *Gapdh*: Forward: CCAAGGTCATCCATGACAA, Reverse: CAGTGAGCTTCCCGTTCAG. Human *Ifnβ1*: Forward: AAACTCATGAGCAGTCTGCA, Reverse: AGGAGATCTTCAGTTTCGGAGG. Human *Cxcl10*: Forward: 5′‐GTGGCATTCAAGGAGTACCTC‐3′, Reverse: 5′‐TGATGGCCTTCGATTCTGGATT‐3′.

**RNA Sequencing**

BJ cells were divided into the following four groups: DMSO, P005091 (20 μM), diABZI (2 μM), and diABZI (2 μM) + P005091 (20 μM). After 2 hours of treatment, total RNA was extracted using the Total RNA Rapid Extraction Kit (GOONIE, China). RNA sequencing and differential gene expression analysis were performed by Tsingke Biotechnology Co., Ltd. (Beijing, China). The four-cluster heatmap of total mRNA and Gene Set Enrichment Analysis (GSEA) was subsequently employed to conduct enrichment analysis on the sets of differentially expressed genes.

**Cell Cytotoxicity Assay**

BJ, RAW 264.7, L929 and HeLa cells were seeded in 96-well plates at 8 × 10^3^ cells/well and cultured until adherence. THP-1 cells were plated at 1 × 10^4^ cells/well. Cells were treated with P005091 (0-50 μM) for 48 hours, followed by incubation with 5% (v/v) CCK-8 reagent (NCM, China) for 2 hours. Absorbance at 450 nm was measured using Varioskan LUX (Thermo Fisher Scientific).

**Luciferase Reporter Assay**

THP-1 Lucia ISG cells (1 × 10^5^ cells per group) were plated in 12-well plates. Following a 2-hour pre-incubation with compounds (0.5-20 μM or 1and 10 μM), S-cddA (0.1 μM) was added and co-incubated for 12 hours. Cells were then centrifuged at 1,500 rpm for 3 minutes. After washing the cell pellet with PBS buffer, chemiluminescence was measured using the Renilla Luciferase Reporter Gene Assay Kit (Beyotime, China).

***In Vivo* Toxicity Testing of P005091**

Male C57BJ6 STING-wild type (stock No: N000013) or STING-deficient mice (stock No: T012747) (6 weeks, n = 3/group) purchased from GemPharmatech Co., Ltd (Nanjing, China) received a daily intraperitoneal injection of P005091 at a dose of 5 mg/kg for 15 consecutive days. On day 16, all mice were euthanized, and colon tissues were harvested for H&E staining.

**DSS-Induced Acute Colitis Model**

Female C57BL/6J mice at 6 weeks were randomly divided into four groups: control, DSS, XL177A (5mg/kg) and DSS+XL177A(5mg/kg), **related to Figure S29**; control, P005091 (5mg/kg), H-151 (5mg/kg), DSS, DSS+P005091(5mg/kg), DSS+H-151(5mg/kg) and DSS+H-151(5mg/kg)+ P005091(5mg/kg), **related to Figure S30**. Mice in the DSS-exposed groups (i.e., DSS and DSS + drug groups) received 3% DSS in drinking water ad libitum for 10 days. Mice in the compound-treated groups received daily intraperitoneal injections of the respective compound intraperitoneally each day for 10 days. Body weight was recorded daily. On day 12, all mice were euthanized. Colon length was measured.

For male STING-deficient mice (stock No: T012747) at 6 weeks divided into two groups: DSS and DSS+P005091, **related to Figure S31**, mice received 3% DSS in drinking water ad libitum for 10 days and daily intraperitoneal injections of P005091 (5 mg/kg) intraperitoneally each day for 10 days. Body weight was recorded daily. On day 11, all mice were euthanized. Colon length was measured.

**H&E and Immunohistochemistry (IHC) Assays**

Paraffin embedding and sectioning of mouse tissues and H&E staining were performed by Service-bio (Wuhan, China). Following dewaxing in xylene (2 × 15 min), tissue sections were rehydrated through a graded ethanol series (100%, 90%, 80%, 75%; 5 minutes per concentration). Antigen retrieval was performed using EDTA in a boiling water bath, after which sections were cooled to room temperature and treated with 3% hydrogen peroxide for 10 minutes. Non-specific binding was blocked with 20% goat serum for 30 minutes prior to incubation with primary antibodies (Phospho-STING, Phospho-IRF3, and Phospho-STAT3 were diluted at 1:100 ) at 4°C overnight. Sections were then incubated with goat anti-mouse/rabbit IgG polymer for 1 hour at room temperature. Chromogenic development utilized 3,3'-diaminobenzidine (DAB) followed by hematoxylin counterstaining, with differentiation in 1% hydrochloric acid-alcohol for 10 seconds. Dehydration was achieved through reverse-graded ethanol (75%, 80%, 90%, 100%; 5 min each) and clearing in xylene (2 × 15 min). After complete air-drying, sections were permanently mounted with neutral resin. Images were performed using a Slide Scanner (KF-PRO-020-HI), with subsequent image visualization and analysis conducted in Kviewer V1 software.

***In Vivo* Pharmacokinetics Study**

The *in vivo* pharmacokinetic experiment was conducted using Sprague Dawley (SD) rats (male, 6-8 weeks) provided from Hangzhou Yanqu Information Technology Co., Ltd., which performed the experimental following procedures. 20 mg/kg intraperitoneal dose of the NTP1 or NTP14, (formulated as a DMSO/HS-15/saline, 5:5:90, v:v:v), were administered to rats. A blood sample was collected at 0, 0.08, 0.25, 0.5, 1, 2, 4, 6, 8 and 24 hours after dosing into EDTA-K2 anticoagulant tubes. After collection, the whole blood was placed in an ice-water bath for temporary cryopreservation. Plasma was subsequently separated by centrifuging the samples at 2000 xg for 10 minutes (4 °C) within 0.5 hours of collection. All samples were analyzed for the compound by LC-MS/MS method. Pharmacokinetic parameters (T_1/2_, T_max_, C_max_, AUC) were calculated using Phoenix WinNonlin software.

**Chemical Synthesis**

**Synthetic Procedure for NTP0.**

2-acetyl-5-chlorothiophene (500 mg) was dissolved in 3 mL of concentrated sulfuric acid with stirring in an ice bath until the solid was completely dissolved. Subsequently, 1 mL of pre-cooled nitric acid was added dropwise to 1 mL of pre-cooled concentrated sulfuric acid to prepare the nitrating mixture. This mixed acid was then added dropwise slowly to the reaction mixture. The reaction progress was monitored by TLC. Upon complete consumption of the starting material, the reaction mixture was slowly added dropwise to ice water, resulting in the precipitation of a pale yellow solid. The mixture was extracted using an ethyl acetate/water system. The organic layer was separated and concentrated under reduced pressure. The resulting crude product was purified by silica gel column chromatography (hexane/ethyl acetate 20:1 v/v) to afford the desired product.

***1-(5-chloro-4-nitrothiophen-2-yl)ethan-1-one* (NTP0)**, 60% yield as a pale yellow solid. ^1^H NMR (400 MHz, CDCl_3_) δ 8.07 (s, 1H), 2.58 (s, 3H).

**General Synthetic Procedure for NTP1-13** **(e.g., NTP1).**

TF-4X0 (50 mg, 0.24 mmol) was dissolved in 4 mL of methanol with stirring at room temperature. Subsequently, 2,3-dichlorobenzenethiol (0.24 mmol) was added and, after dissolution, sodium methoxide (13 mg, 0.3 mmol) was introduced. The reaction mixture was stirred at room temperature for 8 hours, with the progress monitored by TLC. Upon substantial conversion of the starting material, the pH was adjusted to neutrality using 1 N hydrochloric acid. The mixture was extracted using an ethyl acetate/water system. The organic layer was separated and concentrated under reduced pressure. The resulting crude product was purified by silica gel column chromatography (hexane/ethyl acetate 3:1 v/v) to afford the desired product.

***1-(5-((2,3-dichlorophenyl)thio)-4-nitrothiophen-2-yl)ethan-1-one* (NTP1),** 46% yield as a pale yellow solid. ^1^H NMR (400 MHz, CDCl_3_) δ 8.08 (s, 1H), 7.74-7.68 (m, 2H), 7.37 (t, J = 7.9 Hz, 1H), 2.50 (s, 3H); HRMS calcd for [M+Na]^+^: 369.9137, found 369.9160.

***1-(5-((4-methoxyphenyl)thio)-4-nitrothiophen-2-yl)ethan-1-one* (NTP2),** 45% yield as a pale yellow solid. ^1^H NMR (400 MHz, CDCl_3_) δ 8.06 (s, 1H), 7.56 (d, J = 8.6 Hz, 2H), 7.03 (d, J = 8.7 Hz, 2H), 3.89 (s, 3H), 2.47 (s, 3H); HRMS calcd for [M+Na]^+^: 332.0022, found 332.0025.

***1-(5-((2-methoxyphenyl)thio)-4-nitrothiophen-2-yl)ethan-1-one* (NTP3),** 47% yield as a pale yellow solid. ^1^H NMR (400 MHz, CDCl_3_) δ 8.07 (s, 1H), 7.63-7.54 (m, 2H), 7.10-7.04 (m, 2H), 3.87 (s, 3H), 2.47 (s, 3H); HRMS calcd for [M+Na]^+^: 332.0022, found 332.0028.

***1-(5-((3-aminophenyl)thio)-4-nitrothiophen-2-yl)ethan-1-one* (NTP4),** 37% yield as a yellow solid. ^1^H NMR (400 MHz, CDCl_3_) δ 8.06 (s, 1H), 7.29 (t, J = 7.9 Hz, 1H), 7.02 (d, J = 7.3 Hz, 1H), 6.94 (s, 1H), 6.84 (d, J = 8.2 Hz, 1H), 2.48 (s, 3H); HRMS calcd for [M+H]^+^:295.0206, found 295.0238.

***1-(4-nitro-5-(p-tolylthio)thiophen-2-yl)ethan-1-one* (NTP5),** 44% yield as a yellow solid. ^1^H NMR (400 MHz, CDCl_3_) δ 8.07 (s, 1H), 7.53 (d, J = 8.1 Hz, 2H), 7.33 (d, J = 7.9 Hz, 2H), 2.47 (s, 3H), 2.45 (s, 3H). HRMS calcd for [M+H]^+^:316.0073, found 316.0092.

***1-(5-((3-methoxyphenyl)thio)-4-nitrothiophen-2-yl)ethan-1-one* (NTP6),** 45% yield as a pale yellow solid. ^1^H NMR (400 MHz, CDCl_3_) δ 8.07 (s, 1H), 7.48-7.41 (m, 1H), 7.26-7.23 (m, 1H), 7.20-7.17 (m, 1H), 7.11 (dd, J = 8.4, 2.6 Hz, 1H), 3.85 (s, 3H), 2.48 (s, 3H); HRMS calcd for [M+Na]^+^: 332.0022, found 332.0039.

***1-(5-((4-aminophenyl)thio)-4-nitrothiophen-2-yl)ethan-1-one* (NTP7),** 34% yield as a yellow solid. ^1^H NMR (400 MHz, CDCl_3_) δ 8.05 (s, 1H), 7.41-7.35 (m, 2H), 6.78-6.72 (m, 2H), 2.47 (s, 3H)；HRMS calcd for [M+Na]^+^: 317.0025, found 317.0036.

***1-(5-((4-(methylamino)phenyl)thio)-4-nitrothiophen-2-yl)ethan-1-one* (NTP8),** 42% yield as a orange solid. ^1^H NMR (400 MHz, CDCl_3_) δ 8.05 (s, 1H), 7.39 (d, J = 8.6 Hz, 2H), 6.65 (d, J = 8.6 Hz, 2H), 2.90 (s, 3H), 2.46 (s, 3H); HRMS calcd for [M+Na]^+^: 331.0182, found 331.0188.

***1-(5-((2-aminophenyl)thio)-4-nitrothiophen-2-yl)ethan-1-one* (NTP9),** 35% yield as a yellow solid. ^1^H NMR (400 MHz, DMSO-*d*_6_) δ 8.44 (s, 1H), 7.38 (d, J = 7.5 Hz, 1H), 7.31 (t, J = 7.5 Hz, 1H), 6.87 (d, J = 8.0 Hz, 1H), 6.66 (t, J = 7.4 Hz, 1H), 5.82 (s, 2H), 2.50 (s, 3H); HRMS calcd for [M+H]^+^: 295.0206, found 295.0238.

***1-(5-((2,6-dichlorophenyl)thio)-4-nitrothiophen-2-yl)ethan-1-one* (NTP10),** 43% yield as a pale yellow solid. ^1^H NMR (400 MHz, CDCl_3_) δ 8.09 (s, 1H), 7.57 (d, J = 1.1 Hz, 1H), 7.55 (s, 1H), 7.47 (dd, J = 8.9, 7.1 Hz, 1H), 2.50 (s, 3H); HRMS calcd for [M+Na]^+^: 349.9137, found 369.9140.

***1-(5-((2-fluorophenyl)thio)-4-nitrothiophen-2-yl)ethan-1-one* (NTP11),** 36% yield as a white solid. ^1^H NMR (400 MHz, Acetone-*d*_6_) δ 8.32 (s, 1H), 7.88-7.77 (m, 2H), 7.49 (t, J = 8.2 Hz, 2H), 2.55 (s, 3H); HRMS calcd for [M+Na]^+^: 319.9822, found 319.9845.

***1-(5-((2-chlorophenyl)thio)-4-nitrothiophen-2-yl)ethan-1-one* (NTP12),** 45% yield as a pale yellow solid. ^1^H NMR (400 MHz, CDCl_3_) δ 8.08 (s, 1H), 7.76 (d, J = 7.7 Hz, 1H), 7.64 (d, J = 8.0 Hz, 1H), 7.55 (t, J = 7.7 Hz, 1H), 7.43 (t, J = 7.5 Hz, 1H), 2.49 (s, 3H); HRMS calcd for [M+Na]^+^: 335.9526, found 335.9543.

***1-(4-nitro-5-(phenylthio)thiophen-2-yl)ethan-1-one* (NTP13),** 43% yield as a white solid. ^1^H NMR (400 MHz, CDCl_3_) δ 8.07 (s, 1H), 7.67 (dd, J = 5.2, 3.1 Hz, 2H), 7.62-7.51 (m, 3H), 2.48 (d, J = 5.3 Hz, 3H); HRMS calcd for [M+H]^+^: 280.0097, found 280.0084.

**Synthetic Procedure for NTP14.**

NTP0 (50 mg, 0.24 mmol) was dissolved in 4 mL of toluene with stirring at room temperature. Subsequently, 2,3-dichlorophenol (39 mg, 0.24 mmol) was added. After dissolution, potassium carbonate (41.4 mg, 0.30 mmol) was introduced. The reaction mixture was stirred at room temperature for 12 hours, with the progress monitored by TLC. Upon substantial conversion of the thiophene starting material, the mixture was extracted using an ethyl acetate/water system. The organic layer was separated and concentrated under reduced pressure. The resulting crude product was purified by silica gel column chromatography (hexane/ethyl acetate 2:1 v/v) to afford the desired product.

***1-(5-(2,3-dichlorophenoxy)-4-nitrothiophen-2-yl)ethan-1-one*** **(NTP14),** 44% yield as a white solid. ^1^H NMR (400 MHz, CDCl_3_) δ 8.02 (s, 1H), 7.52 (d, J = 6.9 Hz, 1H), 7.34 (t, J = 8.2 Hz, 1H), 7.27 (d, J = 5.4 Hz, 1H), 2.52 (s, 3H)；HRMS calcd for [M+Na]^+^: 353.9365, found 353.9340.

**Synthetic Procedure for NTP15,17.**

Methyl 5-chloro-4-nitrothiophene-2-carboxylate (300 mg, 1.35 mmol) was dissolved in 8 mL of toluene, followed by the addition of 2,3-dichlorobenzenethiol (242.4 mg, 2.30 mmol) and potassium carbonate (224.5 mg, 1.62 mmol). The reaction mixture was stirred at room temperature for 3 hours, with progress monitored by TLC. Upon substantial conversion of the starting material, the reaction mixture was concentrated under reduced pressure. The residue was extracted using an ethyl acetate/water system. The organic layer was separated and concentrated under reduced pressure. The resulting crude product was purified by silica gel column chromatography (hexane/ethyl acetate 3:1 v/v) to afford the desired product.

***Methyl 5-((2,3-dichlorophenyl)thio)-4-nitrothiophene-2-carboxylate* (NTP15),** 58% yield as a pale yellow solid. ^1^H NMR (400 MHz, CDCl_3_) δ 8.18 (s, 1H), 7.71 (d, J = 8.3 Hz, 2H), 7.38 (t, J = 7.9 Hz, 1H), 3.85 (s, 3H); HRMS calcd for [M+Na]^+^: 385.9086, found 385.9086.

TF-4X18 (150mg, 0.41mmol) was dissolved in 8 mL of a methanol-THF-water mixture (2:4:2 v/v/v), followed by the addition of lithium hydroxide (66 mg, 1.65 mmol). The reaction mixture was stirred at room temperature for 6 hours, with progress monitored by TLC. Upon substantial conversion of the thiophene starting material, the pH was adjusted to neutrality using 1 N hydrochloric acid. The mixture was extracted with ethyl acetate and water. The organic layer was washed with brine, separated, and concentrated under reduced pressure to afford the product.

***5-((2,3-dichlor5ophenyl)thio)-4-nitrothiophene-2-carboxylic acid* (NTP17),** 92% yield as a pale brown solid. ^1^H NMR (400 MHz, DMSO-*d*_6_) δ 8.06 (s, 1H), 7.98 (m, 2H), 7.62 (t, J = 8.0 Hz, 1H); HRMS calcd for [M+Na]^+^: 371.8929, found 371.8916.

**Synthetic Procedure for NTP16.**

NTP0 (50 mg, 0.24 mmol) was dissolved in 4 mL of toluene with stirring at room temperature. Subsequently, 2,3-dichlorophenol (39 mg, 0.24 mmol) was added and, after dissolution, potassium carbonate (41.4 mg, 0.30 mmol) was added. The reaction mixture was stirred at room temperature for 12 hours, with the progress monitored by TLC. Upon substantial conversion of the thiophene starting material, the mixture was extracted using an ethyl acetate/water system. The organic layer was separated and concentrated under reduced pressure. The resulting crude product was purified by silica gel column chromatography (hexane/ethyl acetate 2:1 v/v) to afford the desired product.

**Synthetic Procedure for NTP18.**

NTP17 (100 mg, 0.29 mmol) was dissolved in 6 mL of dichloromethane and sequentially treated with HATU (150 mg, 0.72 mmol), N, N-diisopropylethylamine (117 mg, 0.90 mmol) and n-butylamine (42.4 mg, 0.58 mmol). The reaction mixture was stirred at room temperature for 4 hours, with progress monitored by TLC until no further increase in the product spot was observed. The reaction mixture was concentrated under reduced pressure. The residue was extracted using an ethyl acetate/water system. The organic layer was separated and concentrated under reduced pressure. The resulting crude product was purified by column chromatography using a petroleum ether/ethyl acetate system as the eluent to afford the desired product.

***N-butyl-5-((2,3-dichlorophenyl)thio)-4-nitrothiophene-2-carboxamide* (NTP18),** 12% yield as a pale brown solid. ^1^H NMR (400 MHz, CDCl_3_) δ 7.83 (s, 1H), 7.69 (dd, J = 7.9, 2.7 Hz, 2H), 7.36 (t, J = 7.9 Hz, 1H), 5.97 (s, 1H), 3.70 (t, J = 6.3 Hz, 1H), 3.59 (t, J = 6.5 Hz, 1H), 3.38 (dd, J = 13.2, 6.9 Hz, 2H), 1.93-1.85 (m, 1H), 1.76-1.69(m, 1H), 0.94 (t, J = 7.3 Hz, 3H); HRMS calcd for [M+Na]^+^: 426.9715, found 426.9710.

**Synthetic Procedure for NTP19.**

2-chlorothiophene (1 g, 8.43 mmol) was dissolved in 8 mL of chloroform, followed by the addition of 1.07 g (12.65 mmol) of propanoyl chloride. To this mixture, aluminum chloride (2.25 g, 16.86 mmol) was added slowly. The reaction was stirred at room temperature for 8 h, with progress monitored by TLC until the product spot showed no further increase in intensity. The reaction mixture was then concentrated under reduced pressure. The residue was extracted using an ethyl acetate-water system; the organic layer was separated and concentrated. The resulting crude product was purified by silica gel column chromatography (hexane/ethyl acetate 50:1 v/v) to afford the desired product NTP19-P1. Subsequently, 70 mg (0.40 mmol) of NTP19-P1 was dissolved in 3 mL of concentrated sulfuric acid and subjected to nitration using the nitrating agent, equivalent, and reaction conditions identical to those described for the synthesis of NTP0, yielding the intermediate NTP19-P2. Finally, 60 mg (0.27 mmol) of NTP19-P2 was dissolved in 4 mL of methanol and treated with the thiophenolization reagent, equivalent, and reaction conditions following the procedure used for the synthesis of NTP1, affording the final product.

***1-(5-((2,3-dichlorophenyl)thio)-4-nitrothiophen-2-yl)propan-1-one* (NTP19),** 52% yield as a pale yellow solid. ^1^H NMR (400MHz, CDCl_3_) δ 8.09 (s, 1H), 7.74-7.66 (m, 2H), 7.37 (t, J = 7.9 Hz, 1H), 2.85 (q, J = 7.3 Hz, 2H), 1.19 (t, J = 7.3 Hz, 3H); HRMS calcd for [M+Na]^+^: 383.9323, found 383.9324.

**Synthetic Procedure for NTP20-21.**

NTP17 (500 mg, 1.43 mmol) was dissolved in 8 mL of DMF. Subsequently, HATU (412 mg, 1.72 mmol), triethylamine (180.6 mg, 2.15 mmol) and tert-butyl (2-aminoethyl) carbamate (229 mg, 1.43 mmol) were added sequentially. The reaction mixture was stirred at room temperature for 12 h, with progress monitored by TLC until the product spot showed no further increase in intensity. Upon completion, the mixture was extracted using an ethyl acetate/water system. The organic layer was concentrated. The resulting crude product was purified by silica gel column chromatography (hexane/ethyl acetate 5:1 v/v) to afford the desired product NTP21. NTP21 (90 mg, 0.18 mmol) was dissolved in 3 mL of dichloromethane, followed by the addition of 0.5 mL of trifluoroacetic acid. The mixture was stirred at room temperature for 12 h, with reaction progress monitored by TLC until complete consumption of the starting material was observed. The reaction mixture was then concentrated under reduced pressure. The residue was extracted using a dichloromethane /saturated aqueous sodium carbonate solution system. The organic layer was separated, concentrated under reduced pressure, and the resulting residue was dried to afford the product.

***(2-(5-((2,3-dichlorophenyl)thio)-4-nitrothiophene-2- carboxamido)ethyl)carbamate* (NTP21),** 27% yield as a yellow solid. ^1^H NMR (400 MHz, DMSO-*d*_6_) δ 8.84-8.77 (m, 1H), 8.35 (s, 1H), 7.97 (t, J = 8.3 Hz, 2H), 7.61 (t, J = 8.0 Hz, 1H), 6.84 (t, J = 5.4 Hz, 1H), 3.23-3.16 (m, 2H), 3.08-3.01 (m, 2H), 1.35 (s, 9H); HRMS calcd for [M+Na]^+^: 514.0035, found 514.0087.

***N-(2-aminoethyl)-5-((2,3-dichlorophenyl)thio)-4-nitrothiophene-2-carboxamide* (NTP20),** 65% yield as a yellow solid. ^1^H NMR (400 MHz, DMSO-*d*_6_) δ 8.90 (s, 1H), 8.36 (s, 1H), 7.97 (t, J = 8.3 Hz, 2H), 7.62 (t, J = 8.0 Hz, 1H), 2.84 (t, J = 6.2 Hz, 2H); HRMS calcd for [M+H]^+^: 391.9692, found 391.9649.

**Synthetic Procedure for P005091-Biotin.**

NTP20 (30 mg, 0.077 mmol) was dissolved in anhydrous DMF (2 mL). HATU) (28.8 mg, 0.12 mmol, HOBt (14.77 mg, 0.12 mmol), TEA (12.6 mg, 0.124 mL, 0.15 mmol), and biotin (18.8 mg, 0.077 mmol) were added. The resulting mixture was stirred at room temperature for 6 h, and the reaction progress was monitored by TLC. Stirring was continued until no further increase in the intensity of the product spot was observed on TLC. The reaction mixture was then extracted using a DCM/saturated aqueous sodium carbonate solution system. A white solid precipitated in the lower organic layer upon phase separation. The extraction procedure was repeated until TLC analysis of the organic phase indicated the presence of only the product spot. The combined organic layers and the solid precipitate were collected. The liquid fraction was concentrated under reduced pressure using a rotary evaporator, affording the crude product.

***((2,3-dichlorophenyl)thio)-4-nitro-N-(2-(5-((3aS,4S,6aR)-2-oxohexahydro-1Hthieno[3,4-d]imidazol-4-yl)pentanamido)ethyl)thiophene-2-carboxamide* (P005091-Biotin)**, 17% yield as a yellow solid. 1H NMR (400 MHz, DMSO-*d*_6_) δ 8.83 (t, J = 5.6 Hz, 1H), 8.35 (s, 1H), 7.97 (t, J = 8.4 Hz, 2H), 7.86 (t, J = 5.5 Hz, 1H), 7.61 (t, J = 7.9 Hz, 1H), 6.39 (s, 1H), 6.34 (s, 1H), 4.31–4.26 (m, 1H), 4.13–4.06 (m, 1H), 3.24–3.18 (m, 2H), 3.18–3.12 (m, 2H), 3.09–3.02 (m, 1H), 2.04 (t, J = 7.4 Hz, 2H), 1.64–1.43 (m, 4H), 1.31–1.24 (m, 2H); HRMS calcd for [M+Na]^+^: 640.0287, found 640.0351.

**Synthetic Procedure for NTP1-Cys, NTP14-Cys and NTP16-Cys.**

NTP1, NTP14 or NTP16 (0.33 mmol) was dissolved in anhydrous DMF (1 mL). Boc-L-cysteine (73.5 mg, 0.33 mmol) and potassium carbonate (62.1 mg, 0.45 mmol) were added. The resulting mixture was stirred at room temperature for 2 h, and the reaction progress was monitored by TLC. Stirring was continued until no further increase in the intensity of the product spot was observed on TLC. The mixture was concentrated and then purified by silica gel column chromatography (DCM/methanol 8:1 v/v) to afford the desired product.

***S-(5-acetyl-3-nitrothiophen-2-yl)-N-(tert-butoxycarbonyl)-L-cysteine* (NTP1-Cys and NTP14-Cys)**, 35% yield as a yellow solid (NTP1-Cys), 34% yield as a yellow solid (NTP14-Cys) HRMS calcd for [M-H]^-^: 389.0, found 389.0 for NTP1-Cys; HRMS calcd for [M-H]^-^: 389.0, found 389.0 for NTP14-Cys.

***O-(5-acetyl-3-nitrothiophen-2-yl)-N-(tert-butoxycarbonyl)-L-serinate* (NTP16-Cys),** 38% yield as a yellow solid. HRMS calcd for [M-H]^-^: 405.0, found 405.0.

**Table S2: Information of reagents and resources**

| REAGENT or RESOURCE | SOURCE | IDENTIFIER |
| --- | --- | --- |
| Antibodies |  |  |
| Rabbit anti TMEM173/STING Polyclonal antibody | Proteintech | Cat#19851-1-AP; RRID: AB_10665370 |
| Rabbit anti- Phospho-STING (Ser365) | Cell Signaling Technology | Cat#72971; RRID: AB_2799831 |
| Rabbit anti- TBK1/Nak | Cell Signaling Technology | Cat#3504; RRID: AB_2255663 |
| Rabbit anti-p-TBK1/p-Nak (Ser172) | Cell Signaling Technology | Cat#5483; RRID: AB_10693472 |
| Rabbit anti-GAPDH | Cell Signaling Technology | Cat#2188; RRID: AB_561053 |
| Rabbit anti-Vinculin | Proteintech | Cat# 26520-1-AP; RRID: AB_ 2868558 |
| Rabbit anti-HA | Proteintech | Cat#22291-1-AP; RRID: AB_11042321 |
| Rabbit anti-Biotin Antibody | Abcam | Cat#ab53494; RRID: AB_867860 |
| Rabbit anti-DYKDDDDK tag Polyclonal antibody | Proteintech | Cat#20543-1-AP; RRID: AB_11232216 |
| Rabbit anti Phospho-IRF3 (Ser396) Polyclonal antibody | Proteintech | Cat#29528-1-AP; RRID: AB_2935415 |
| Rabbit anti Phospho-STAT3-Y705 | ABclonal | Cat#AP0705; RRID: AB_2863810 |
| Mouse anti-GM130 | BD Transduction Laboratories™ | Cat#610822; RRID: AB_398141 |
| Mouse anti-DYKDDDDK tag Polyclonal antibody | Proteintech | Cat#66008-4-Ig; RRID: AB_2918475 |
| Mouse-anti-HA | Proteintech | Cat#66006-2-Ig; RRID: AB_2881490 |
| Goat Anti-Mouse IgG H&L (Alexa Fluor® 488) | Abcam | Cat#ab150113; RRID: AB_2576208 |
| Goat Anti-Rabbit IgG H&L (Alexa Fluor® 647) | Abcam | Cat#ab150079; RRID: AB_2722623 |
| Myc-Tag Rabbit mAb | ABclonal | Cat#AB9106; RRID: AB_307014 |
| Recombinant Protein |  |  |
| Recombinant Human Sting (N-Sumo-6His) | Novoprotein | CR43 |
| Recombinant Human Sting (Full Length) | ReadCrystal | N/A |
| Chemicals and Reagents |  |  |
| DMSO | Sigma-Aldrich | Cat#D4540 |
| XL177A | TargetMol,USA | Cat#T9122, CAS 2417089-74-6 |
| MSA-2 | TargetMol,USA | Cat#T8798, CAS 129425-81-6 |
| H-151 | TargetMol,USA | Cat#T5674, CAS 941987-60-6 |
| DMXAA | TargetMol,USA | Cat#T6273, CAS 117570-53-3 |
| 2',3'-cGAMP sodium | TargetMol,USA | Cat#T10065L, CAS 2734858-36-5 |
| diABZI | TargetMol,USA | Cat#T11035, CAS 2138498-18-5 |
| Lipopolysaccharides | TargetMol,USA | Cat#T11855 |
| Poly(I:C) | MedChemExpress | Cat#HY-107202 |
| Dextran Sulfate Sodium Salt | Yeasen | Cat#60316ES |
| S-cddA | Autonomous synthesis | N/A |
| DMEM, high glucose | Bio-Channel | Cat#BC-M-005 |
| RPMI 1640 | Bio-Channel | Cat#BC-M-023 |
| Fetal Bovine Serum(Characterized) | Bio-Channel | Cat#BC-SE-FBS07 |
| Penicillin-streptomycin | New Cell & Molecular Biotech | Cat# C100C5 |
| L-glutamine | MedChemExpress | Cat#HY-N0390, CAS 56-85-9 |
| Cell Total RNA Quick Extraction Kit | Goonie Biotech Co.,Ltd | Cat# 400-100 |
| Uni All-in-One First-Strand cDNA Synthesis SuperMix for qPCR | TranGen Biotech Co.,Ltd | Cat# AU341-02 |
| PerfectStart® Green qPCR SuperMix | TranGen Biotech Co.,Ltd | Cat# AQ601-02-V2 |
| DEPC H_2_O | Beyotime | Cat# R0021 |
| QuickBlock Immunostaining Blocking Solution | Beyotime | Cat#P0260 |
| BSA | New Cell & Molecular Biotech Co.,Ltd | Cat#WB6504 |
| Triton X-100 | Sigma-Aldrich | Cat#X100 |
| DAPI | ThermoFisher | Cat#D3571 |
| SDS-PAGE Sample Loading Buffer，5X | New Cell & Molecular Biotech Co.,Ltd | Cat# WB2001 |
| Non-Reducing SDS-PAGE Sample Loading Buffer,5X | Biosharp Life science | Cat# BL511B |
| Non-Reducing Native-PAGE Sample Loading Buffer,5X | New Cell & Molecular Biotech Co.,Ltd | Cat# WB3002 |
| Three-color prestained protein Marker 10~250 kDa | New Cell & Molecular Biotech Co.,Ltd | Cat#P9006 |
| Cell Lysis Buffer for Western and IP without Inhibitors | New Cell & Molecular Biotech Co.,Ltd | Cat# P70100 |
| Protease and Phosphatase Inhibitor Cocktail | New Cell & Molecular Biotech Co.,Ltd | Cat#P002 |
| Sodium deoxycholate | Chemxyz Biotech Co.,Ltd | Cat#P332012 |
| NCM Universal Antibody Diluent | New Cell & Molecular Biotech Co.,Ltd | Cat#WB500D |
| Protein A+G Agarose (Fast Flow,for IP) | Beyotime | Cat# P2055 |
| L- Glycine | Beyotime | Cat#ST1522; CAS 56-40-6 |
| THAM | D&B | Cat#K6870022; CAS 77-86-1 |
| SDS | AmBeed | Cat#A120709; CAS 151-21-3 |
| Methanol | Energy Chemical | Cat#A040901; CAS 67-56-1 |
| Ethanol | Energy Chemical | Cat#W310136; CAS 64-17-5 |
| Sodium taurochenodeoxycholate | D&B | Cat#P332015; CAS 6009-98-9 |
| Xylene | Energy Chemical | Cat#W320218; CAS 1330-20-7 |
| Poly-L-lysine | Macklin | Cat#P875129; CAS 25988-63-0 |
| Histiocyte fixative solution | Leagene | Cat#DF0135 |
| EDTA antigen retrieval solution (50×) | Solarbio | Cat# C1034 |
| Ready-to-use normal goat serum | BOSTER | Cat#AR0009 |
| PV-6000 Universal Kit (Mouse/Rabbit Polymer Assay System) | ZSGB-Bio | Cat#PV-6000 |
| DAB kit | ZSGB-Bio | Cat#ZL1-9017 |
| Eosin stain | Servicebio | Cat#G1002 |
| Hematoxylin staining solution | Servicebio | Cat#G1004 |
| Standard Matrigel | Corning | Cat#356234 |
| Stain Buffer (FBS) | BD Transduction Laboratories™ | Cat#554656 |
| Neutral resin | Solarbio | Cat# G8590 |
| Compounds for STING-CTD mass spectrometry screening analysis | TargetMol | N/A |
| Kolliphor HS-15 | Sigma-Aldrich | Cat#42966 |
| LB244 | MedChemExpress | Cat#HY-156117 |
| USP7 (K208-E560) | MedChemExpress | Cat#HY-P74478 |
| Critical Commercial Assays |  |  |
| Mouse lFN-β1 ELlSA Kit | JONLNBIO | Cat#JL52208 |
| Mouse CXCL10 ELiSA Kit | JONLNBIO | Cat#JL54728 |
| Mouse IL-6 ELISA Kit | JONLNBIO. | Cat#JL20268 |
| BCA protein assay kit | KeyGen Biotech | Cat#KGP903 |
| Renilla-Lumi™ | Beyotime | Cat#RG062S |
| FastPure Cell/Tissue Total RNA Isolation Kit V2 | Vazyme | Cat# RC112-01 |
| Experimental models: Cell lines |  |  |
| L929 cells | ATCC | Cat#CCL-1; RRID: CVCL_0462；  Cell Origin: Mouse connective tissue  (Purchase date: 2023.06) |
| RAW264.7 cells | ATCC | Cat#TIB-71; RRID: CVCL_0493；  Cell Origin: Mouse tumor induced by Abelson murine leukemia virus (Purchase date: 2023.06) |
| THP-1 cells | Pricella | Cat#CL-0233; RRID: CVCL_0006；  Cell Origin: Human acute monocytic leukemia cells (Purchase date: 2023.09) |
| DC2.4 cells | Pricella | Cat#CL-0545; RRID: CVCL_J409;  Cell Origin: Mouse bone marrow isolate (Purchase date: 2025.01) |
| BJ cells | Wanwu Biology | Cat#Delf-10527; RRID: CVCL_C8QN;  Cell Origin: Human foreskin (Purchase date: 2025.03) |
| HeLa cells | Pricella | Cat#CL-0101; RRID: CVCL_0030; Cell Origin: Human cervical cancer tissue (Purchase date: 2024.09) |
| HEK293F cells | Pricella | Cat#CL-0544; RRID: CVCL_6642; Cell Origin: Human embryonic kidney (Purchase date: 2025.04) |
| THP-1 Lucia ISG cells | InvivoGen  (provided by Junmin Quan’s Lab) | Cat#thpl-nfkb; RRID: CVCL_X590;  Cell Origin: Human acute monocytic leukemia cells (Provided date: 2023.10) |
| COS7-HA STING cells | Pricella (COS7 cells) | Cat#CL-0069; RRID: CVCL_0224;  Cell Origin: Kidney tissue of African green monkey (Purchase date: 2025.03) |
| HEK293T-Flag STING cells | Pricella (HEK293T cells) | Cat#CL-0005; RRID: CVCL_0063;  Cell Origin: Human embryonic kidney (Purchase date: 2023.10) |
| HeLa-STING KO cells | Tiebang Kang’s Lab | Provide date: 2025.06 |
| Recombinant DNA |  |  |
| pCDNA3.1-3HA-hSTING(WT) | Kidan Biosciences Inc | N/A |
| pCDNA3.1-3HA-hSTING(C206/309A) | Kidan Biosciences Inc | N/A |
| pCDNA3.1-3HA-hSTING(C257/309A) | Kidan Biosciences Inc | N/A |
| pCDNA3.1-3HA-hSTING(C292/309A) | Kidan Biosciences Inc | N/A |
| pCDNA3.1-3Flag-mSTING(WT) | Kidan Biosciences Inc | N/A |
| pCDNA3.1-3HA-mSTING(WT) | Kidan Biosciences Inc | N/A |
| pCDNA3.1-3HA-mSTING(C291/308A) | Kidan Biosciences Inc | N/A |
| pCDNA3.1-3Flag-hSTING(WT) | Junmin Quan’s Lab | N/A |
| pCDNA3.1-3Flag-hSTING(C12A) | Junmin Quan’s Lab | N/A |
| pCDNA3.1-3Flag-hSTING(C29A) | Junmin Quan’s Lab | N/A |
| pCDNA3.1-3Flag-hSTING(C64A) | Junmin Quan’s Lab | N/A |
| pCDNA3.1-3Flag-hSTING(C88A) | Junmin Quan’s Lab | N/A |
| pCDNA3.1-3Flag-hSTING(C91A) | Junmin Quan’s Lab | N/A |
| pCDNA3.1-3Flag-hSTING(C148A) | Junmin Quan’s Lab | N/A |
| pCDNA3.1-3Flag-hSTING(C206A) | Junmin Quan’s Lab | N/A |
| pCDNA3.1-3Flag-hSTING(C257A) | Junmin Quan’s Lab | N/A |
| pCDNA3.1-3Flag-hSTING(C292A) | Junmin Quan’s Lab | N/A |
| pCDNA3.1-3Flag-hSTING(C309A) | Junmin Quan’s Lab | N/A |
| pCDNA3.1-3Flag-hSTING(All C→S) | Junmin Quan’s Lab | N/A |
| pCDNA3.1-Myc-USP7 | Kidan Biosciences Inc | N/A |
| Software and Algorithms |  |  |
| GraphPad Prism | GraphPad 8.0 Software | GraphPad |
| SPSS | SPSS 20.0 Software | SPSS |
| ImageJ | National Institutes of Health | ImageJ |
| FV31S-SW Version: 2.1.1.98 | OLYMPUS | FV31S-SW |
| KViewer V1 | KFBIO | KViewer |
| QuPath | QuPath-0.3.0 Software | QuPath |

**Table S3: Abbreviations**

| **Abbreviations** | **Full Name** |
| --- | --- |
| **STING** | stimulator of interferon genes |
| **cGAS** | cyclic GMP-AMP synthase |
| **dsDNA** | double-stranded DNA |
| **cGAMP** | 2’3’-cyclic GMP-AMP |
| **I-IFN** | type I interferons |
| **SLE** | systemic lupus erythematosus |
| **AGS** | Aicardi-Goutières syndrome |
| **NASH** | non-alcoholic steatohepatitis |
| **IBD** | inflammatory bowel disease |
| **ER** | endoplasmic reticulum |
| **CTD** | C-terminal domain |
| **TBK1** | TANK-binding kinase 1 |
| **IRF3** | interferon regulatory factor 3 |
| **TMD** | transmembrane domain |
| **MS** | mass spectrometry |
| **mSTING** | murine STING |
| **hSTING** | human STING |
| **DMSO** | dimethyl sulfoxide |
| **CETSA** | cell thermal shift assay |
| **DSF** | differential scanning fluorimetry |
| **IP** | immunoprecipitation |
| **WB** | western blot |
| **TREX1** | three prime repair exonuclease 1 |
| **IHC** | immunohistochemistry |
| **DAB** | diaminobenzidine |
| **P-STING** | phosphorylated STING |
| **IF** | immunofluorescence |
| **WT** | wild type |
| **ISGs** | interferon-stimulated genes |
| **DAMPs** | damage-associated molecular patterns |
| **DSS** | dextran sulfate sodium |
| **P-IRF3** | phosphorylated IRF3 |
| **P-STAT3** | phosphorylated STAT3 |
| **SPR** | surface plasmon resonance |
| **SAR** | structure-activity relationship |
| **LC/MS** | liquid chromatograph mass spectrometer |
| **TLC** | thin layer chromatography |
| **HRMS** | High resolution mass spectrometry |
| **^1^H NMR** | ^1^hydrogen nuclear magnetic resonance |
| **THF** | tetrahydrofuran |
| **HATU** | 1-[Bis(dimethylamino)methylene]-1H-1,2,3-triazolo [4,5-b] pyridinium 3-oxid hexafluorophosphate |
| **DMF** | N, N-Dimethylformamide |
| **TEA** | triethylamine |
| **HOBt** | 1-hydroxybenzotriazole |
| **DCM** | dichloromethane |
| **NS** | normal saline |

**Spectrogram for structural characterization of compounds (^1^H NMR and HRMS)**

**NTP1 (P005091)**

**
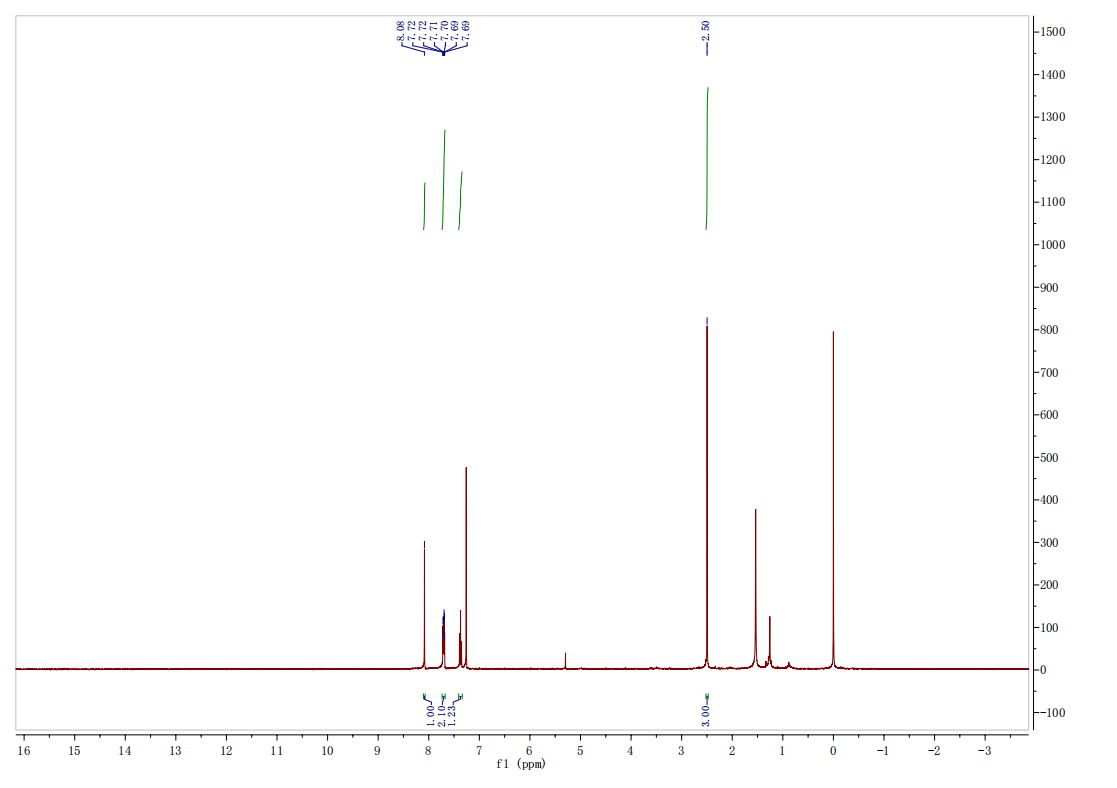
**

**
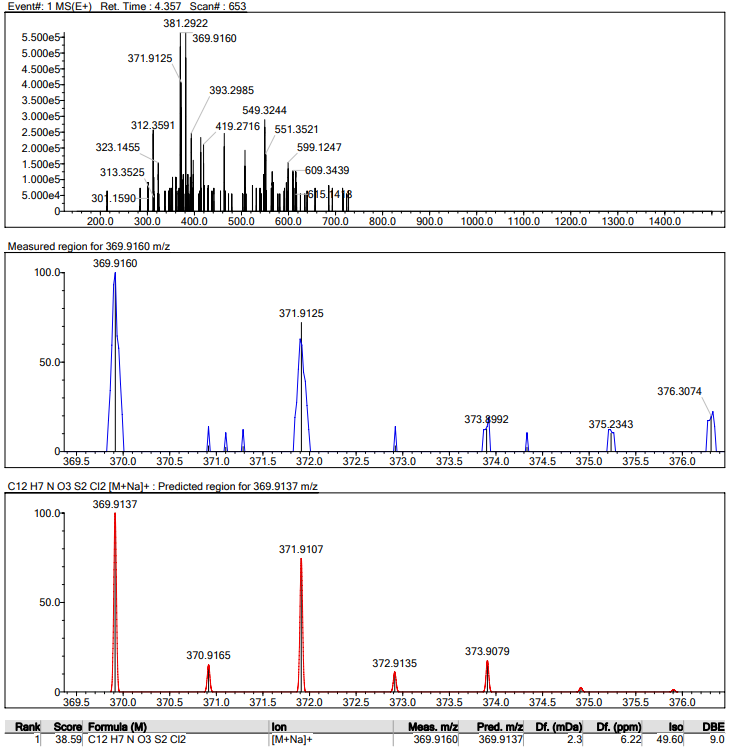
**

**NTP2**

**
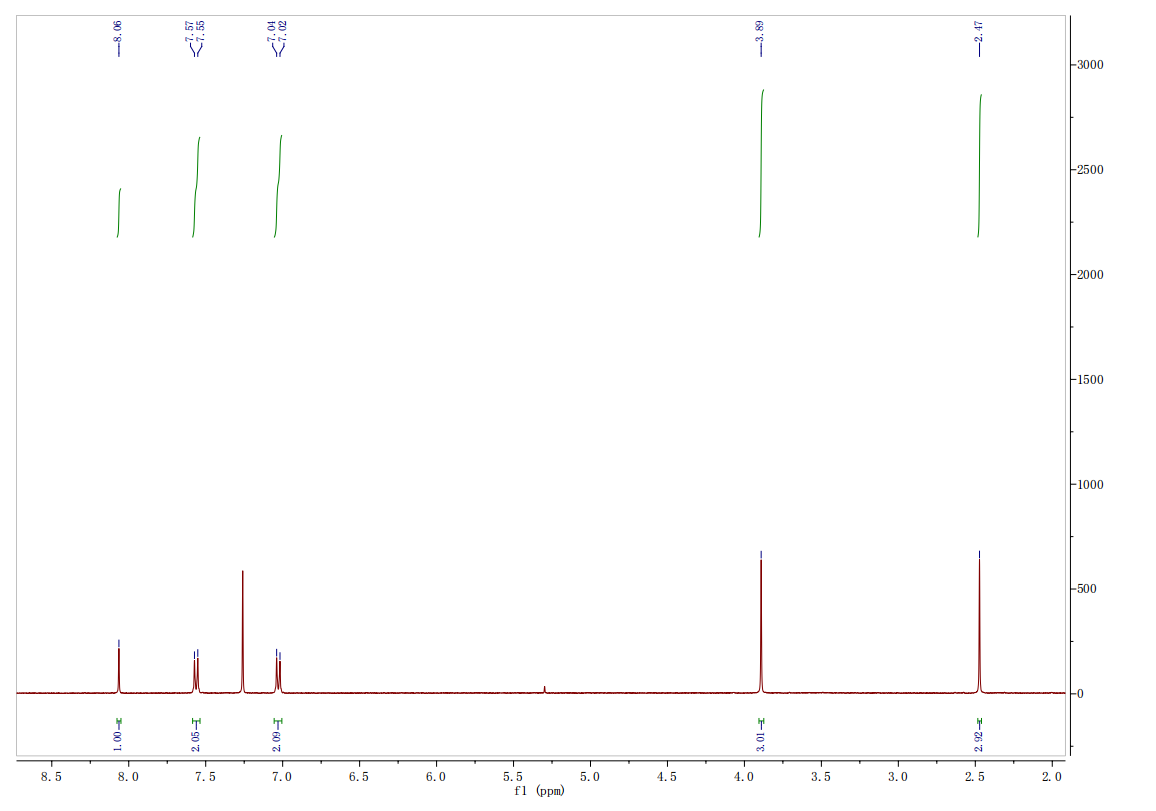
**

**
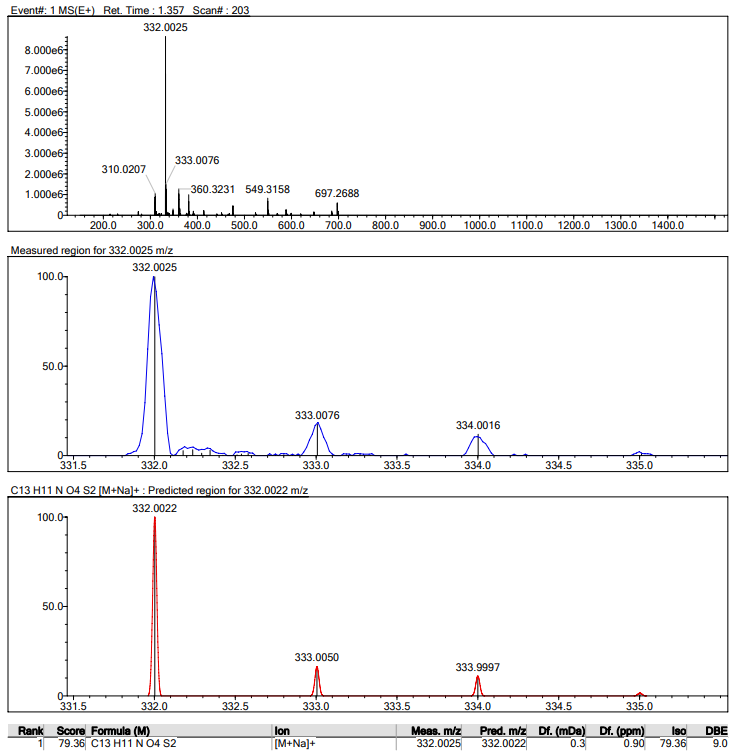
**

**NTP3**

**
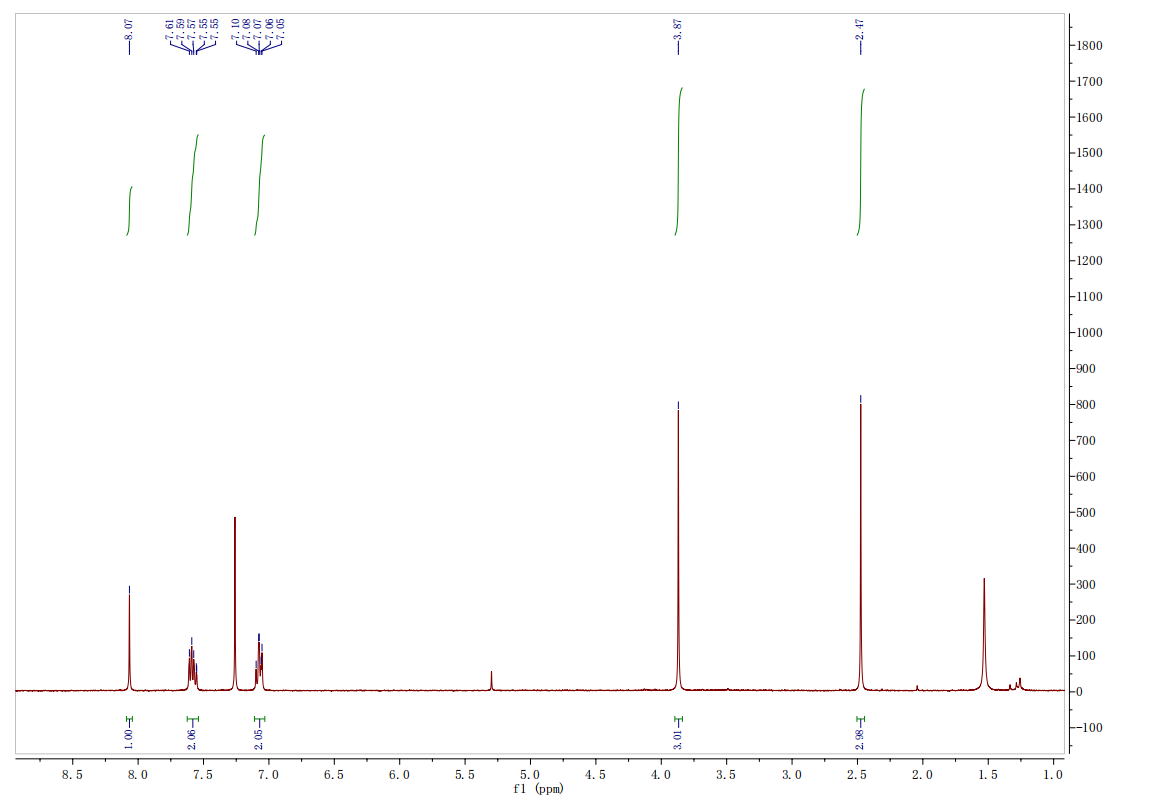
**

**
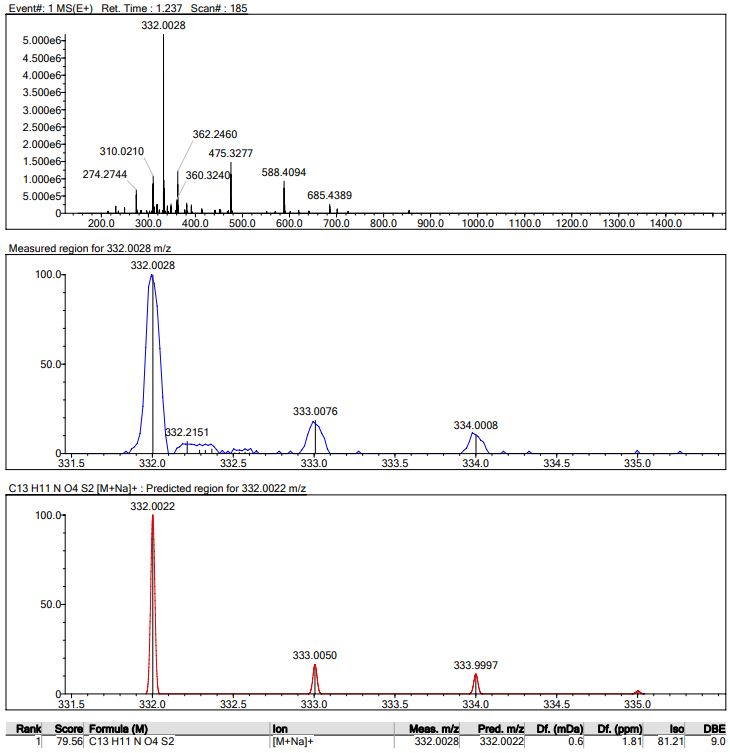
**

**NTP4**

**
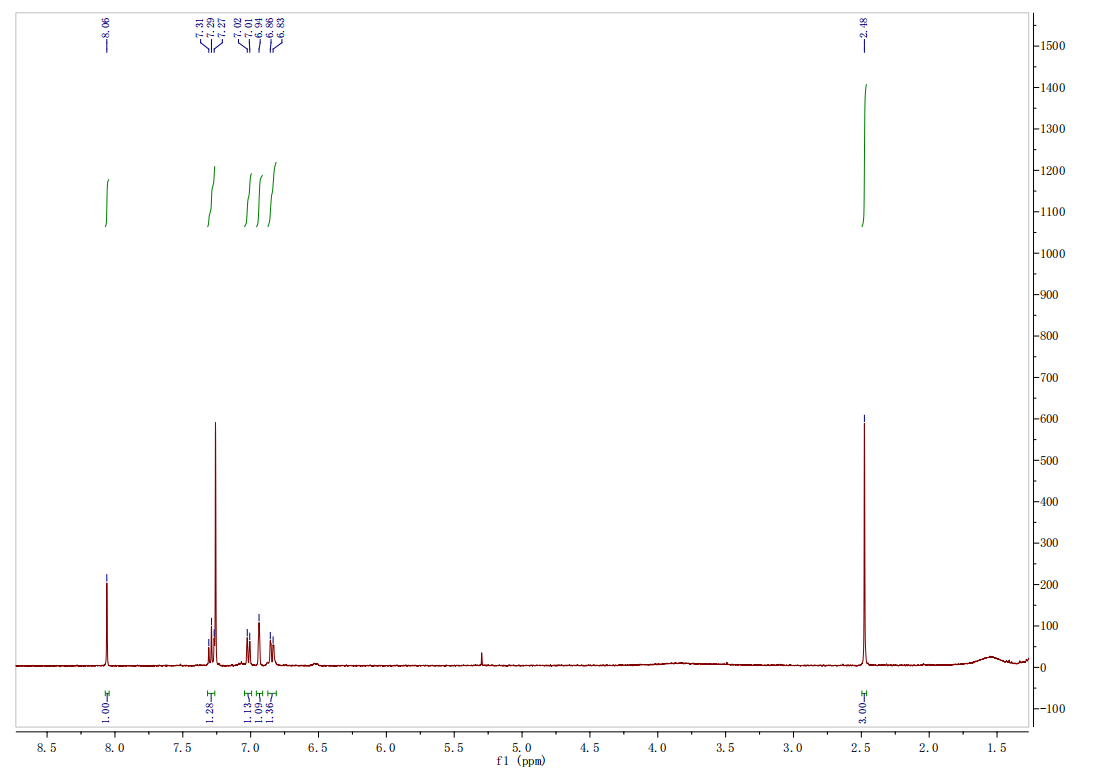
**


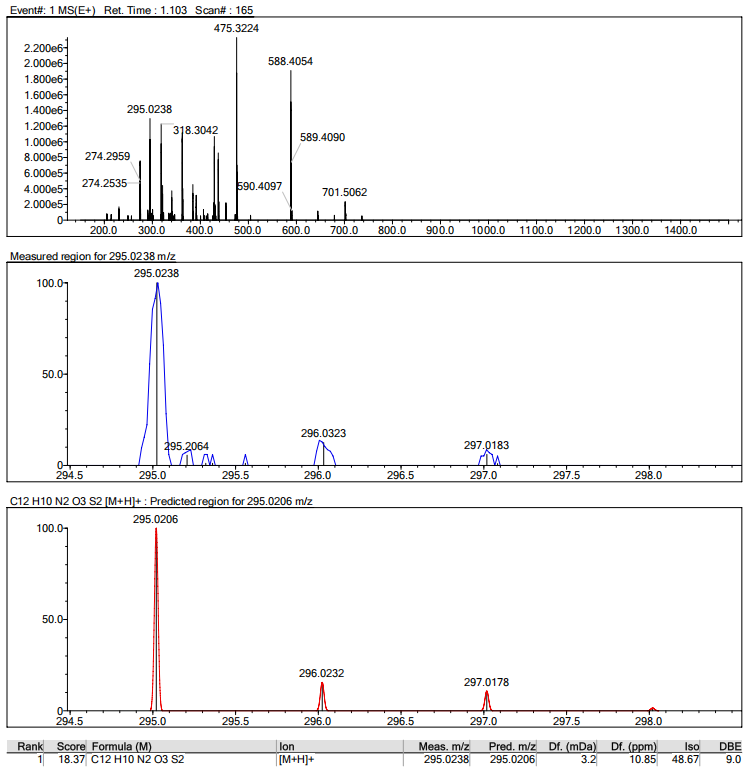


**NTP5**

**
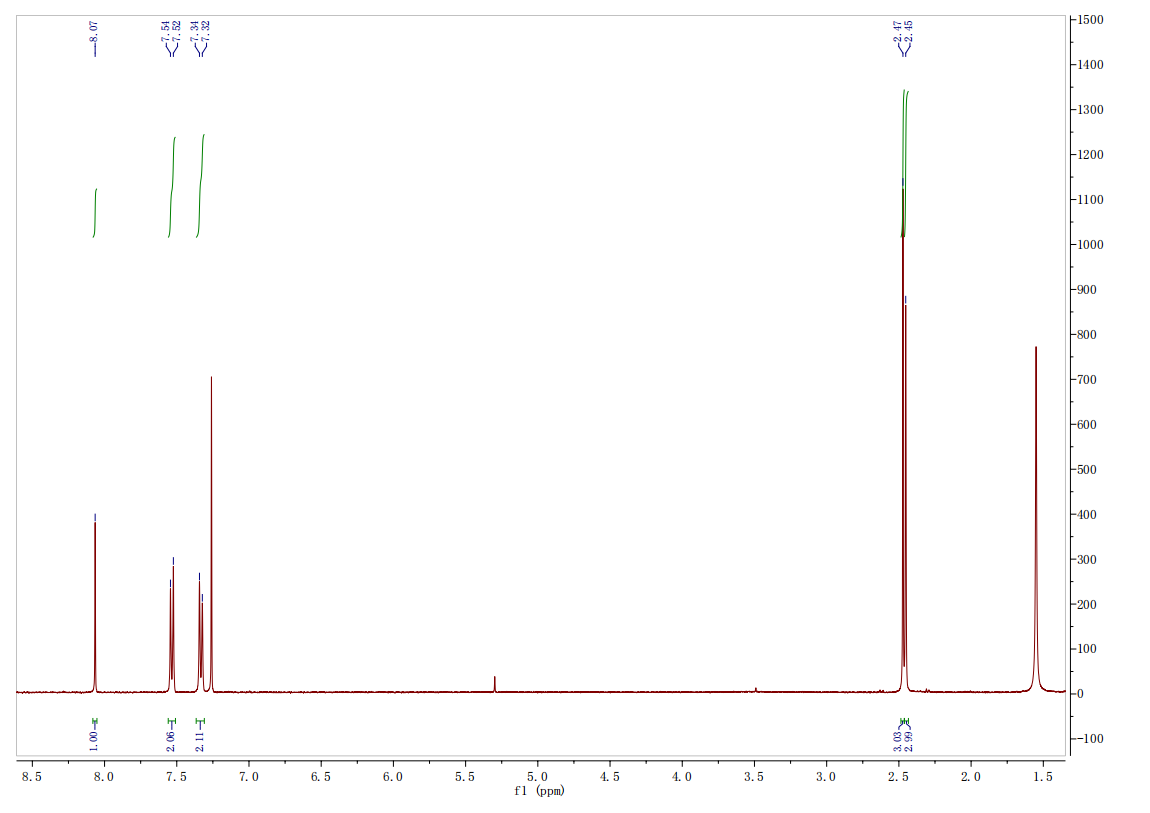
**


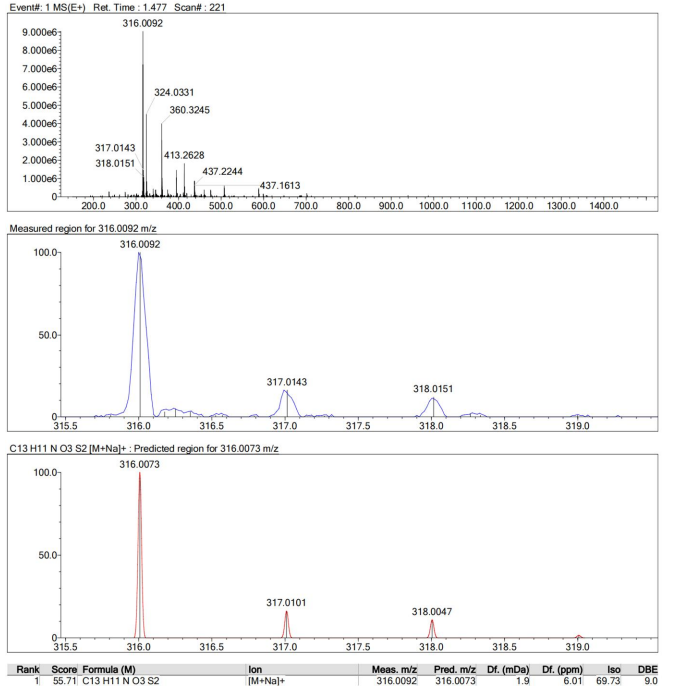


**NTP6**

**
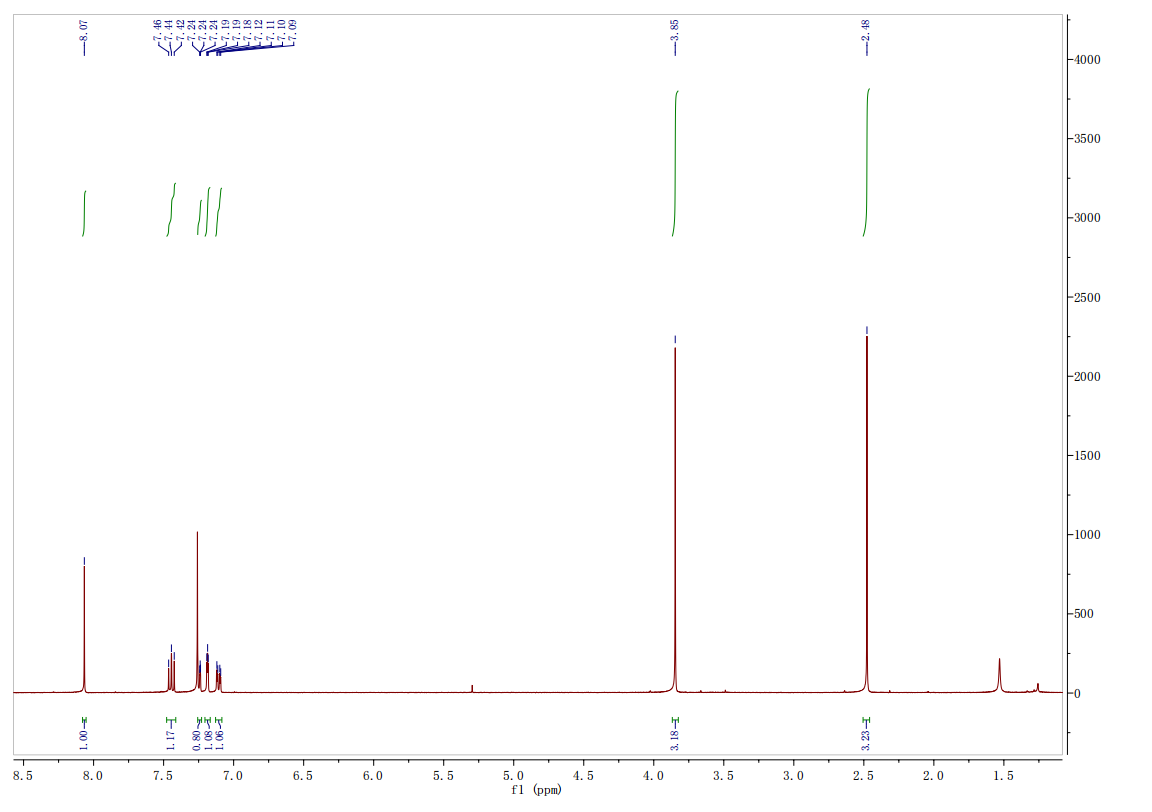
**

**
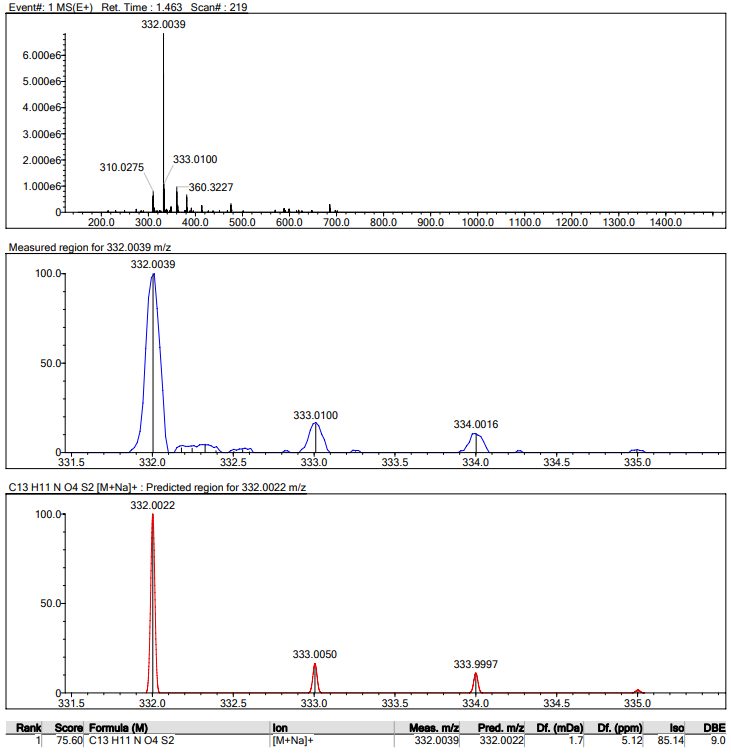
**

**NTP7**

**
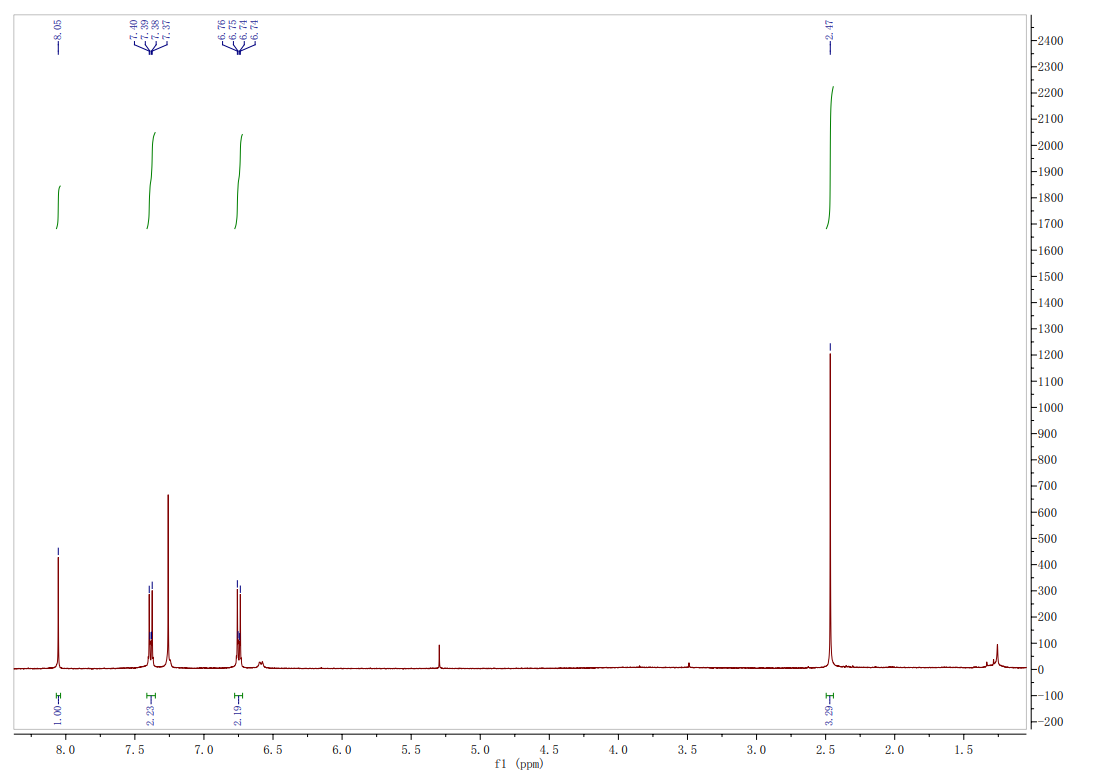
**

**
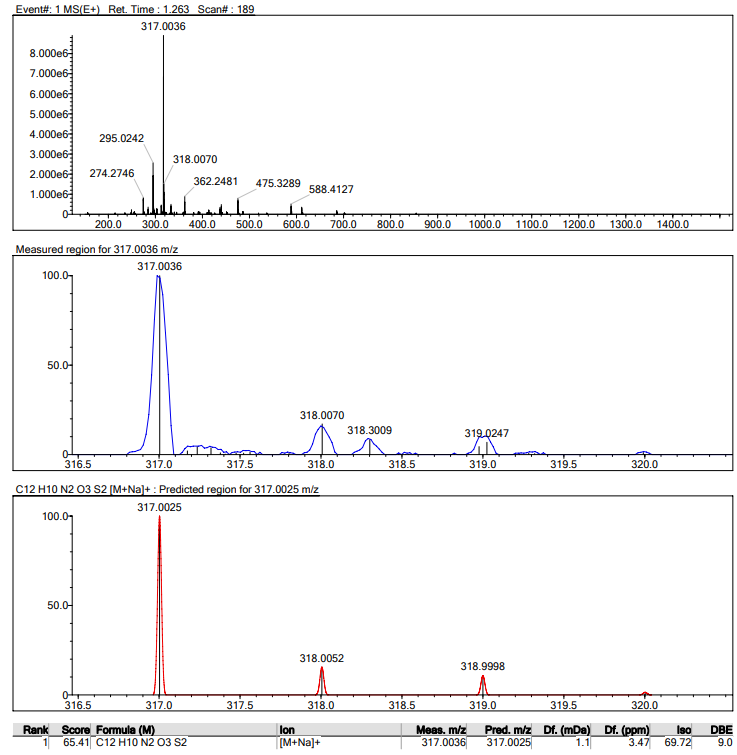
**

**NTP8**

**
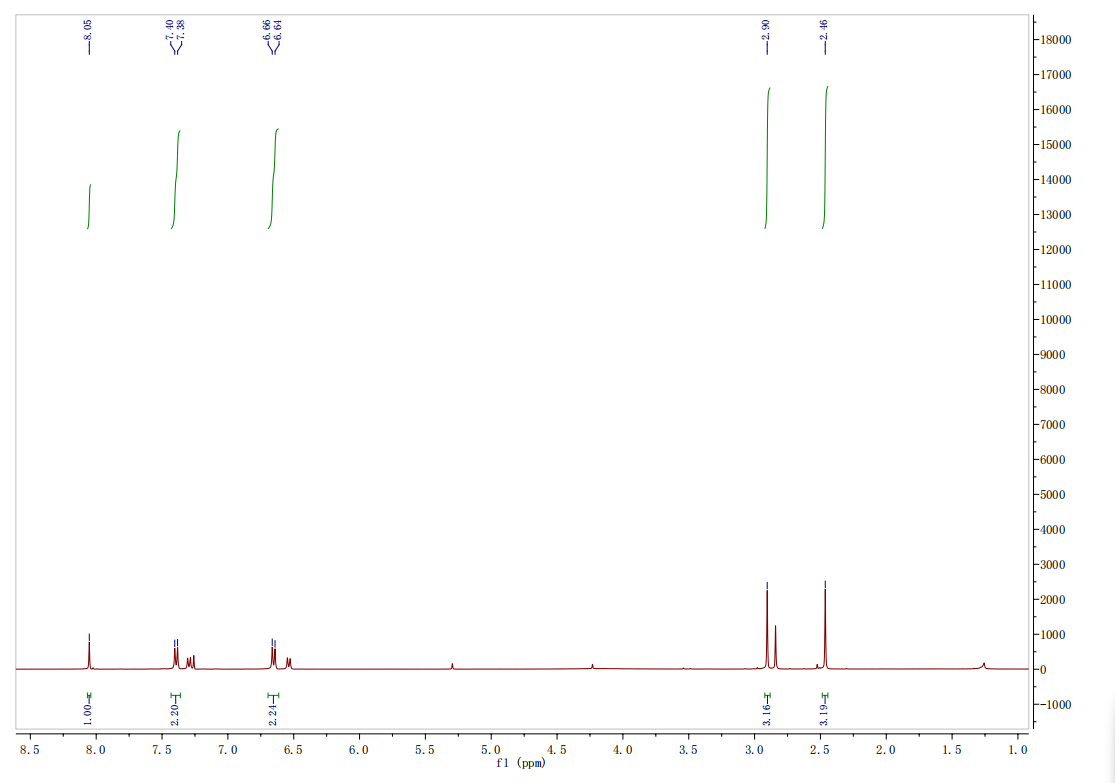
**

**
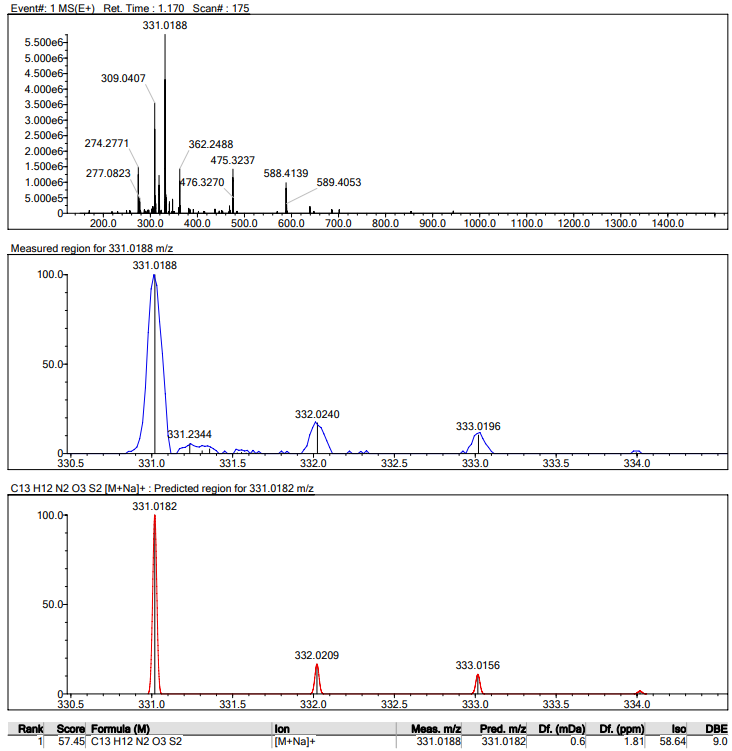
**

**NTP9**

**
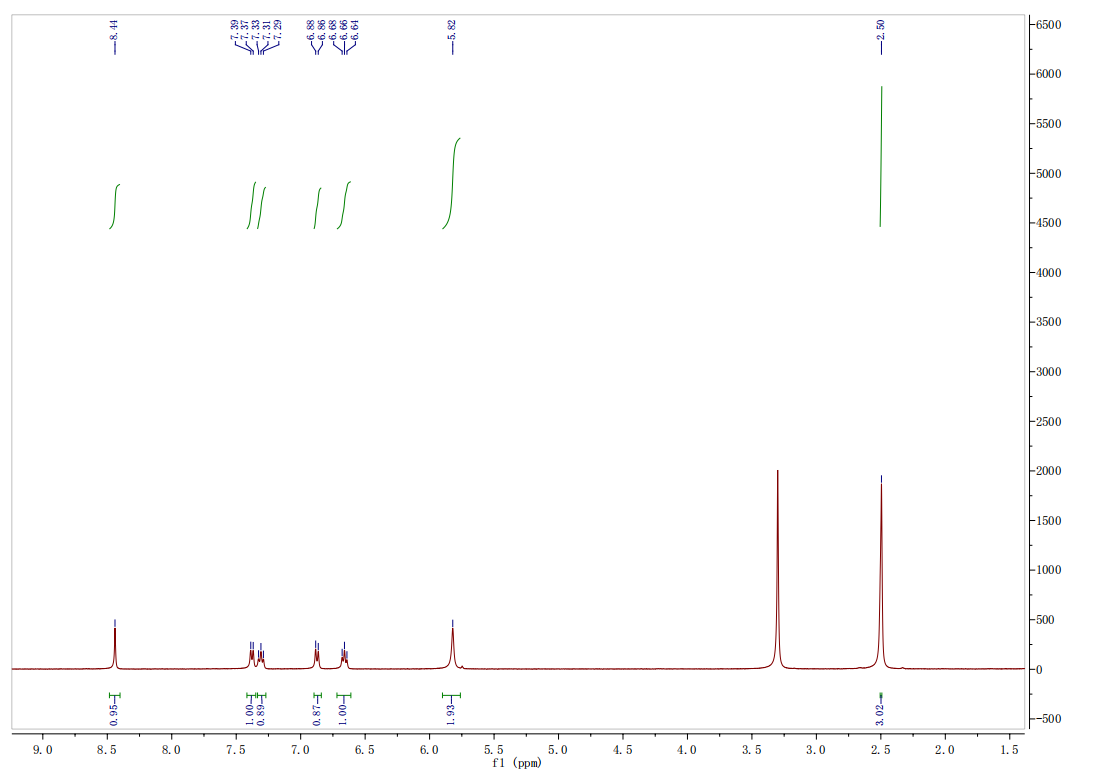
**

**
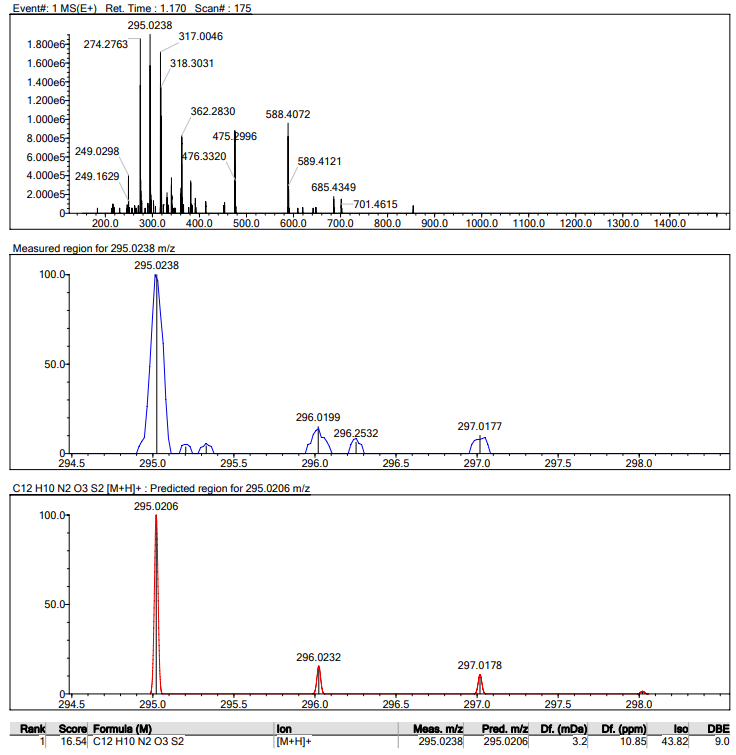
**

**NTP10**

**
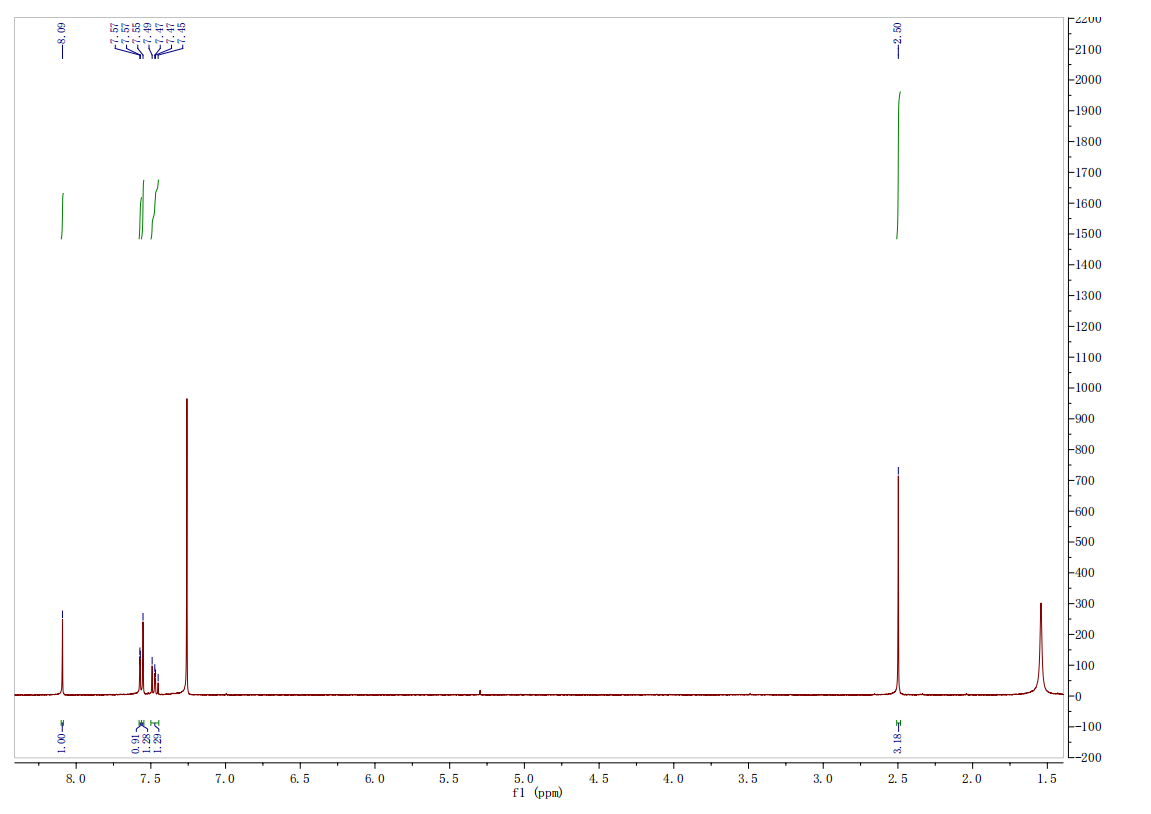
**

**
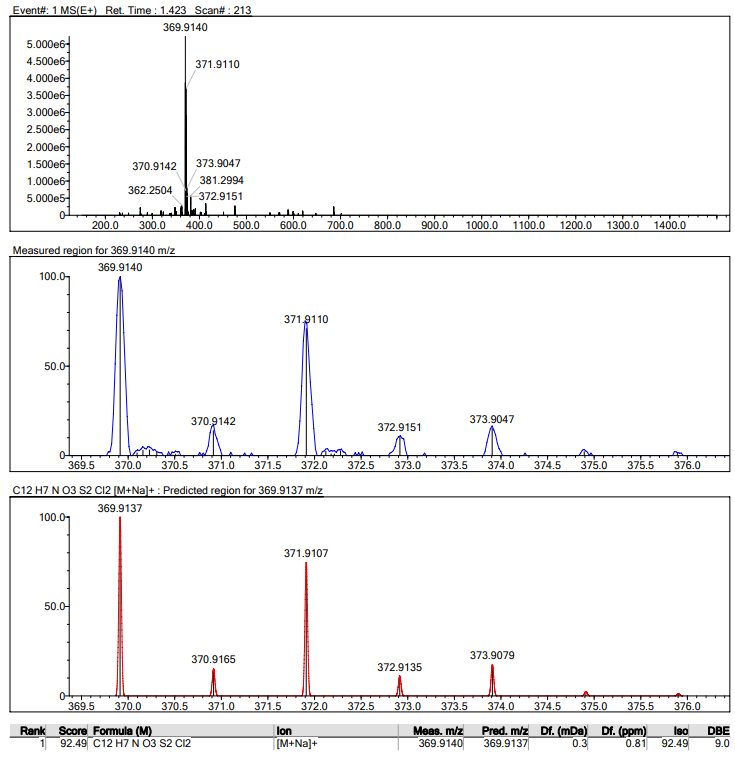
**

**NTP11**

**
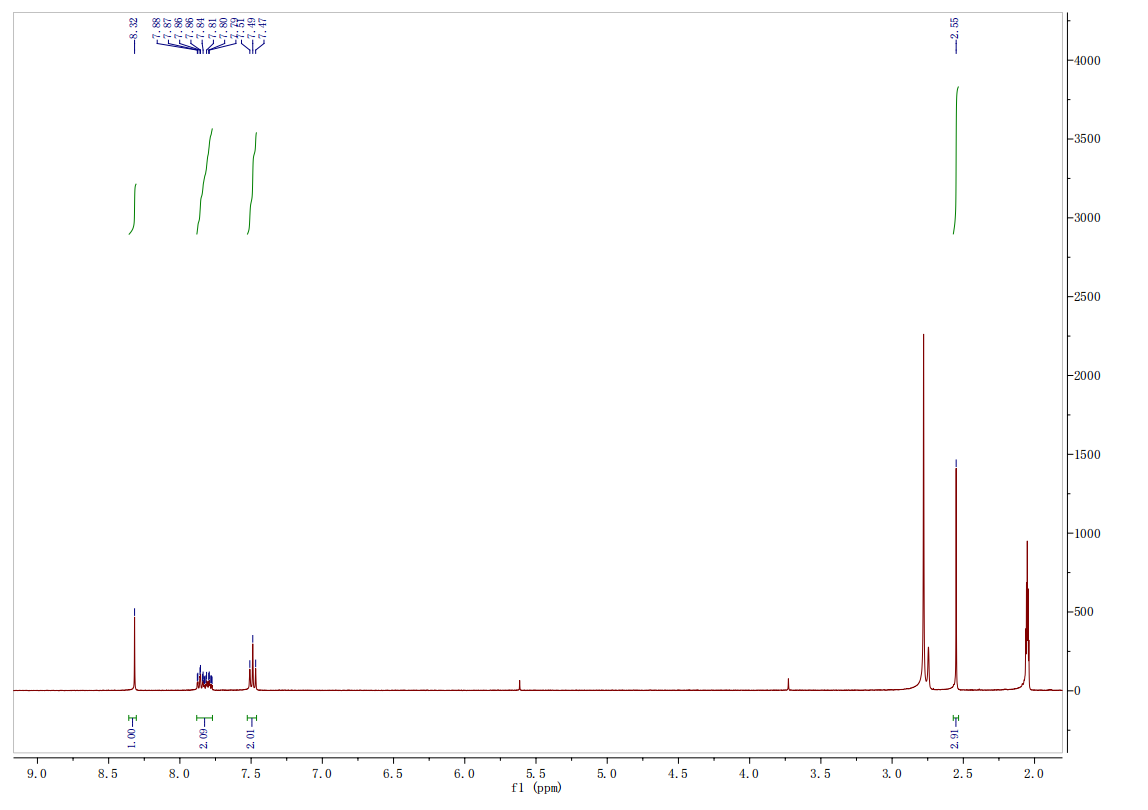
**

**
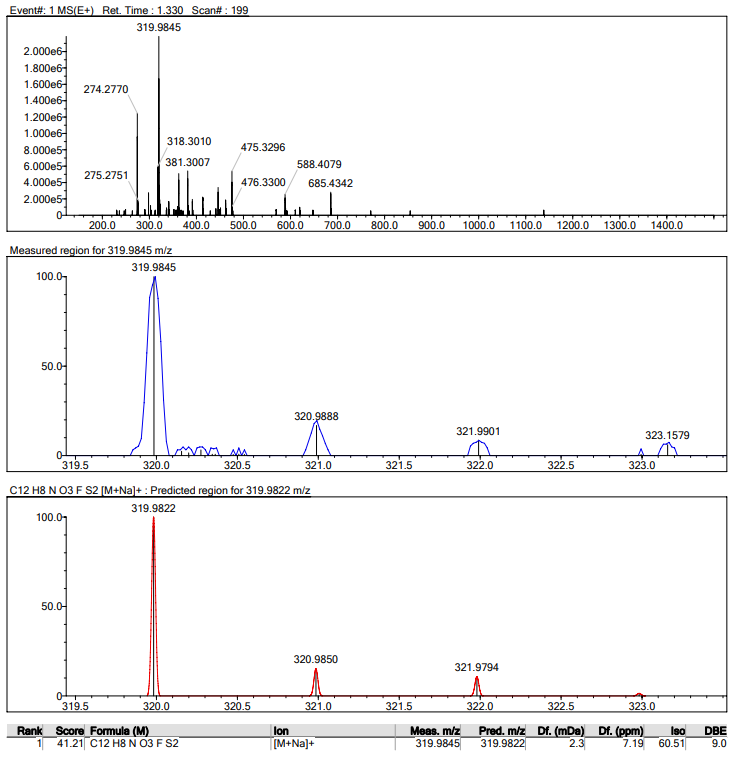
**

**NTP12**

**
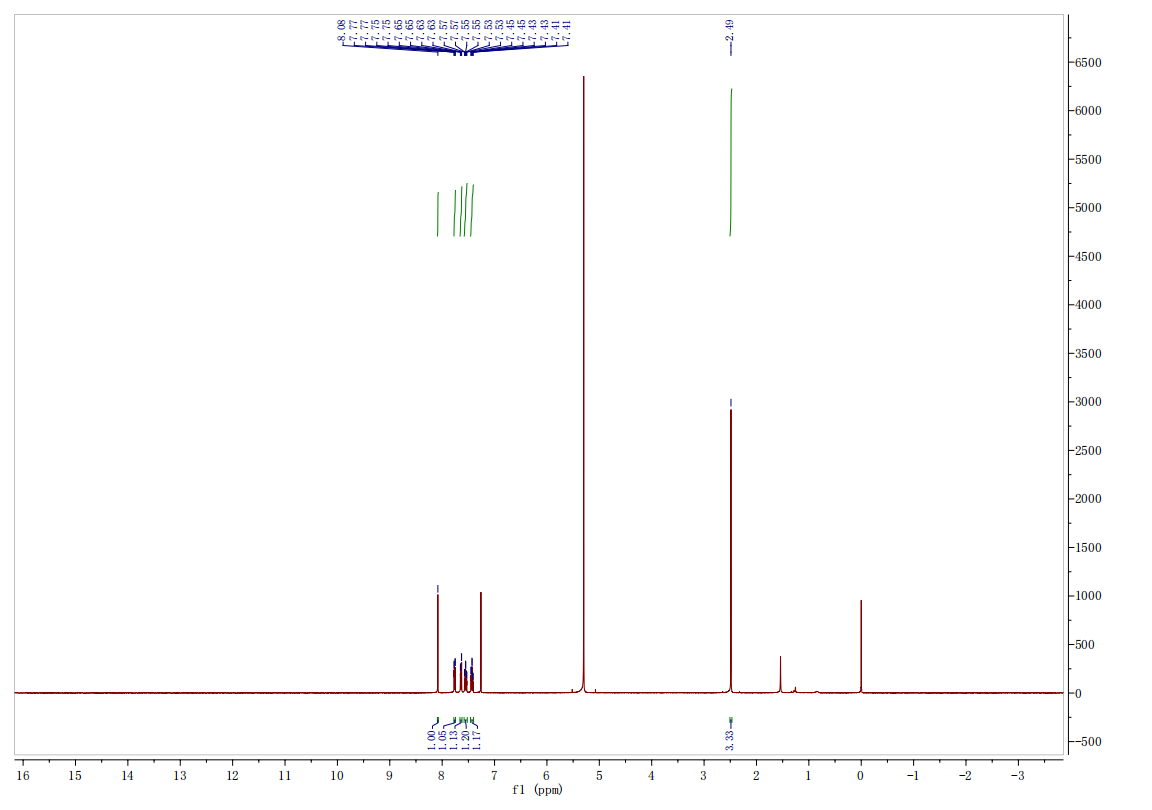
**

**
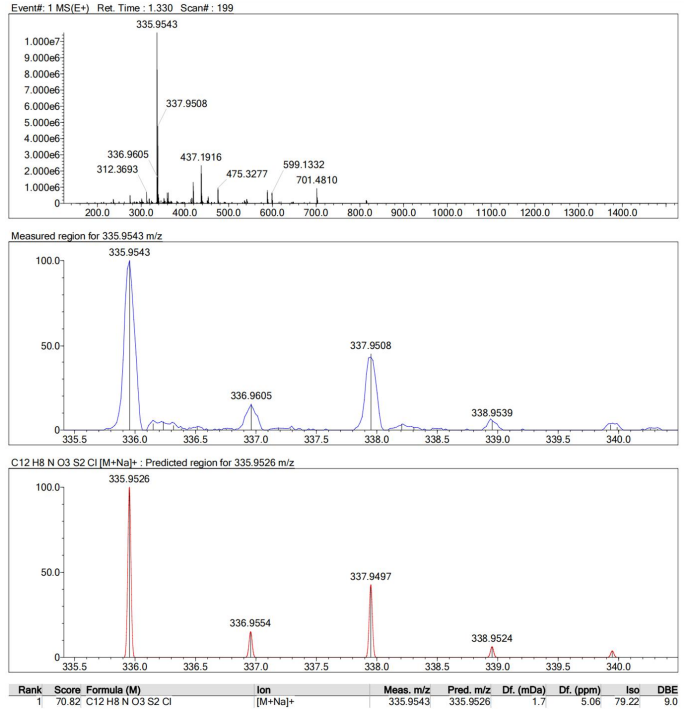
**

**NTP13**

**
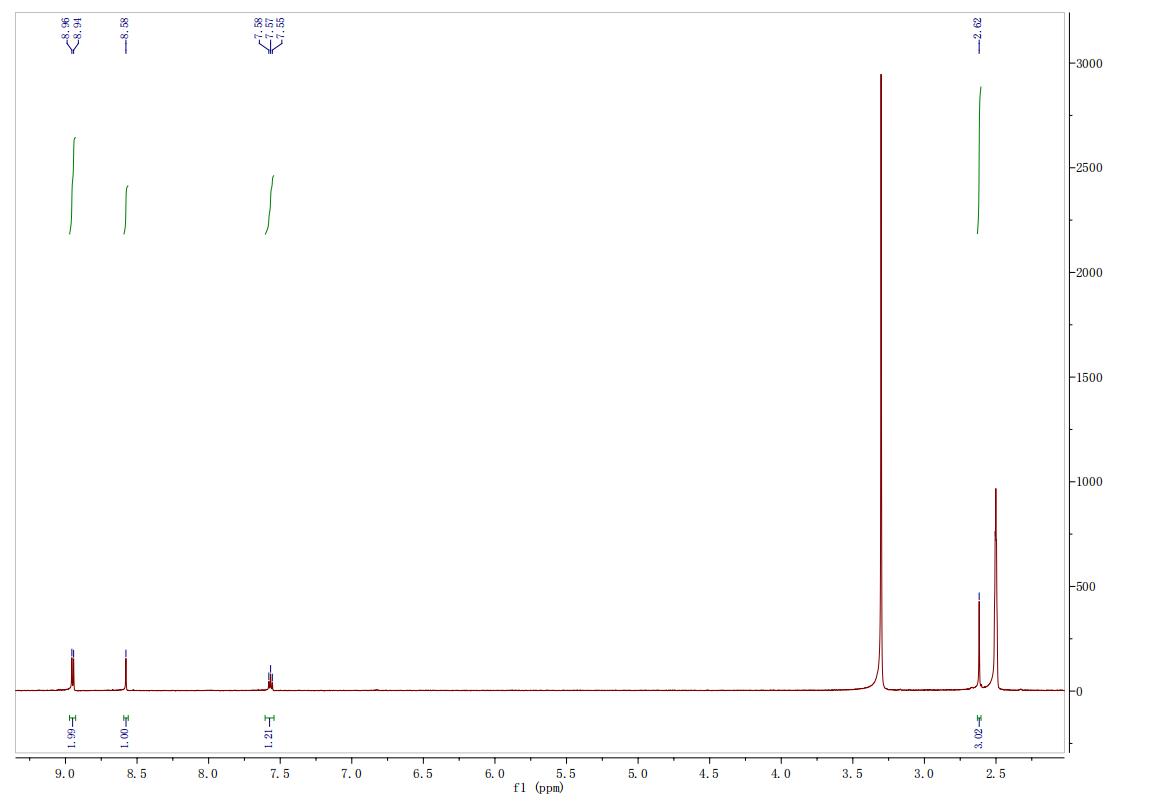
**

**
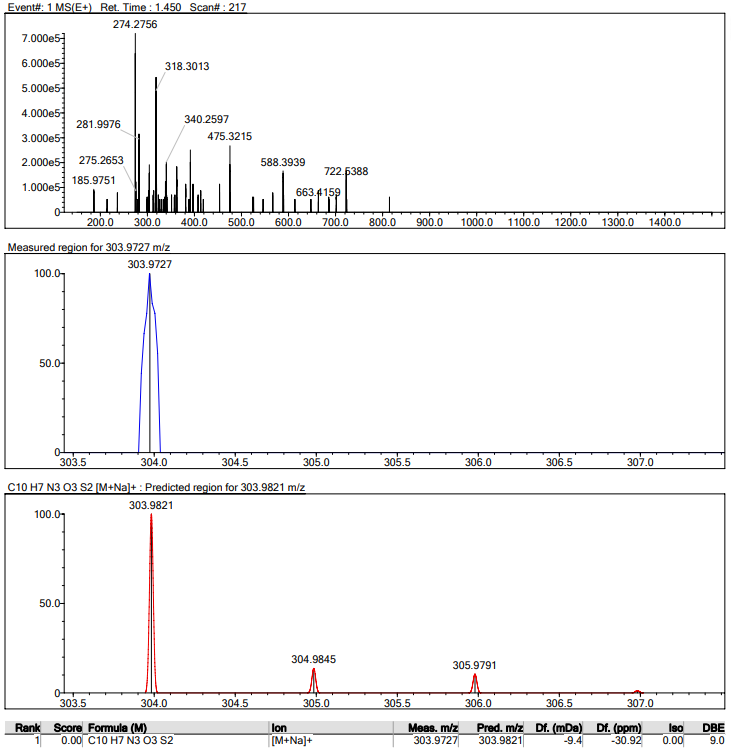
**

**NTP14**

**
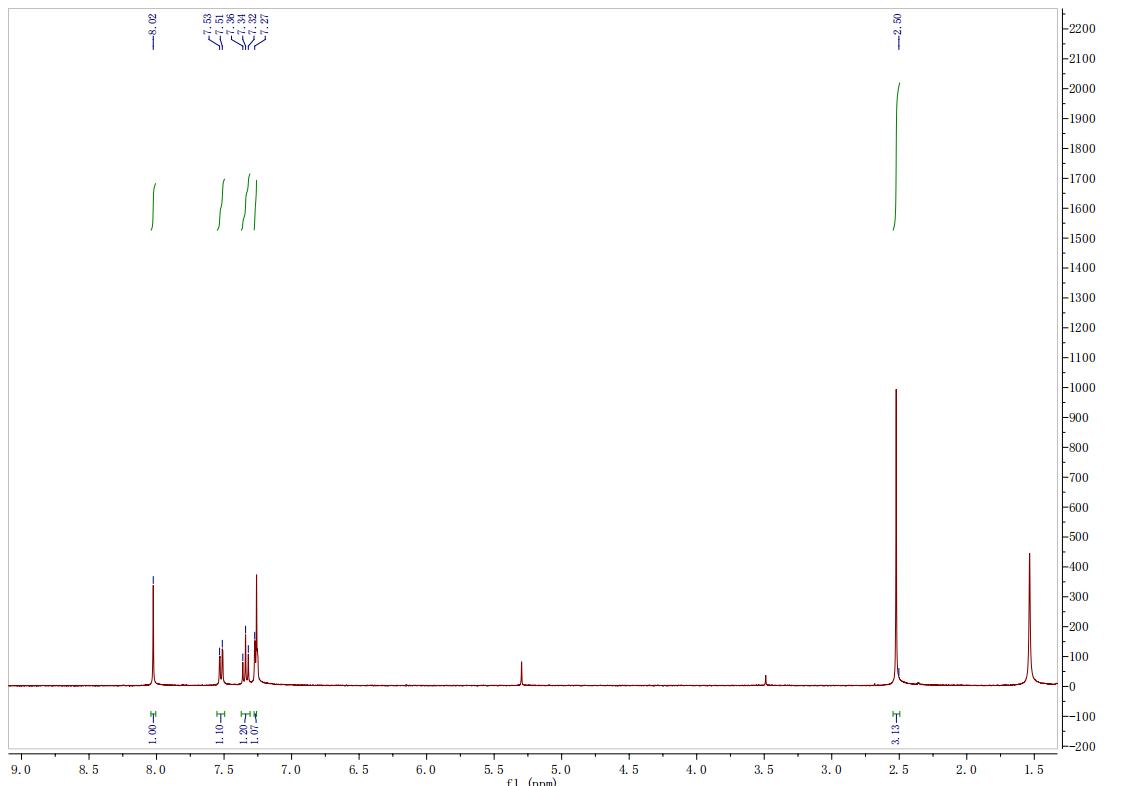
**

**
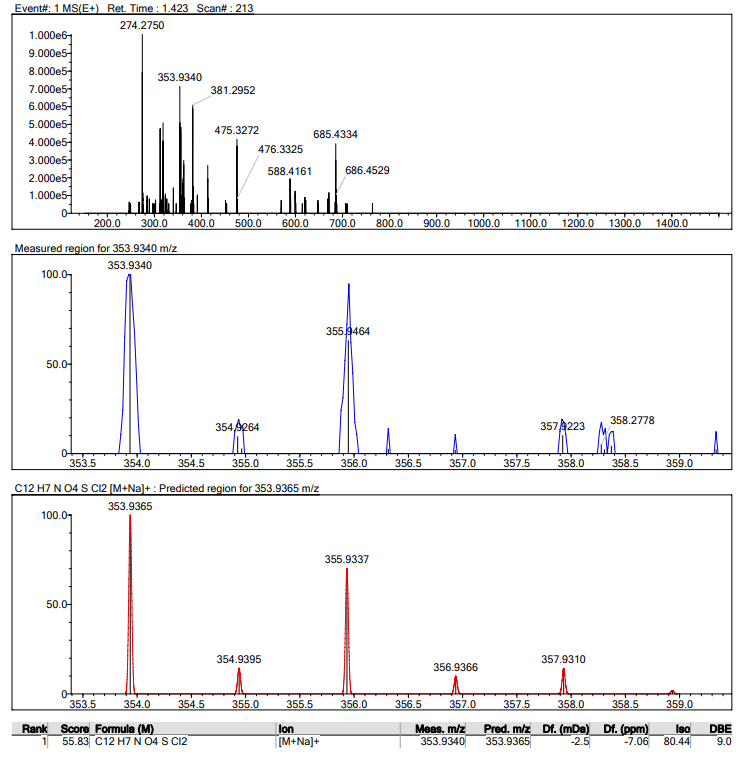
**

**NTP15**

**
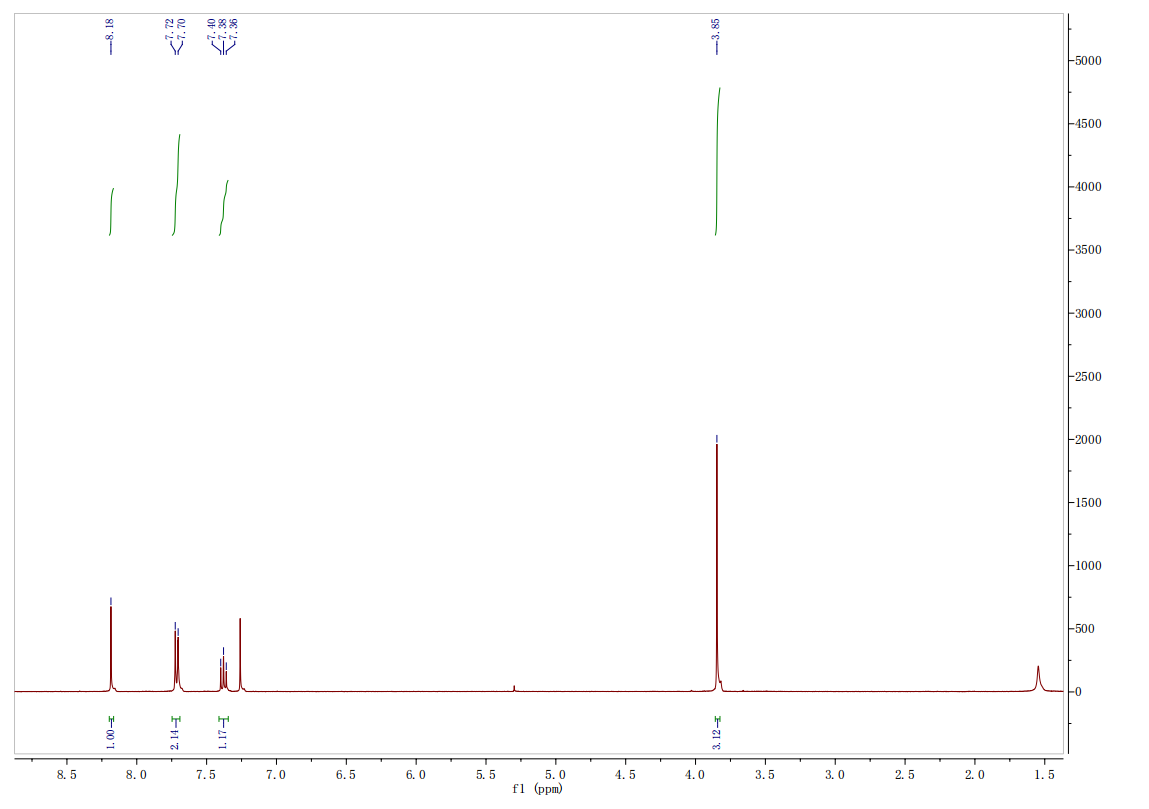
**

**
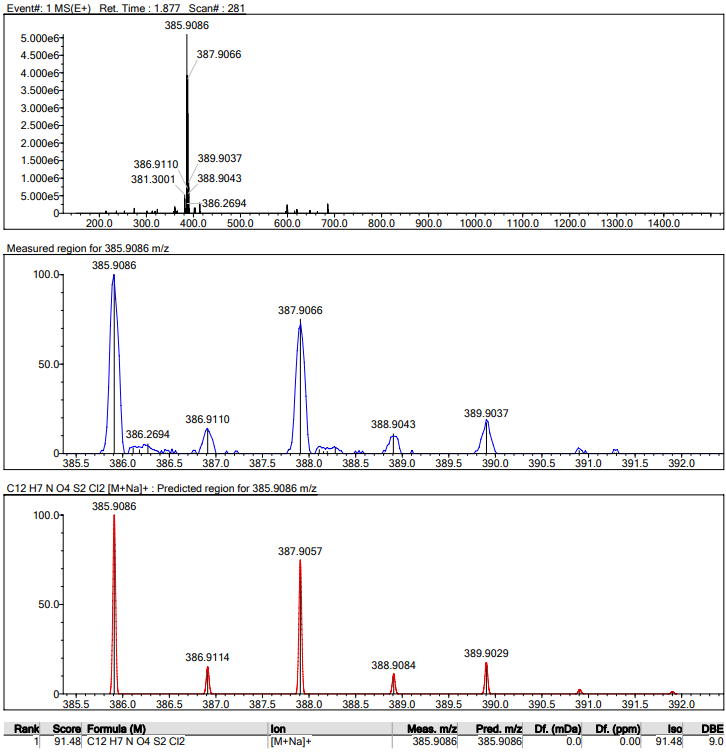
**

**NTP16**

**
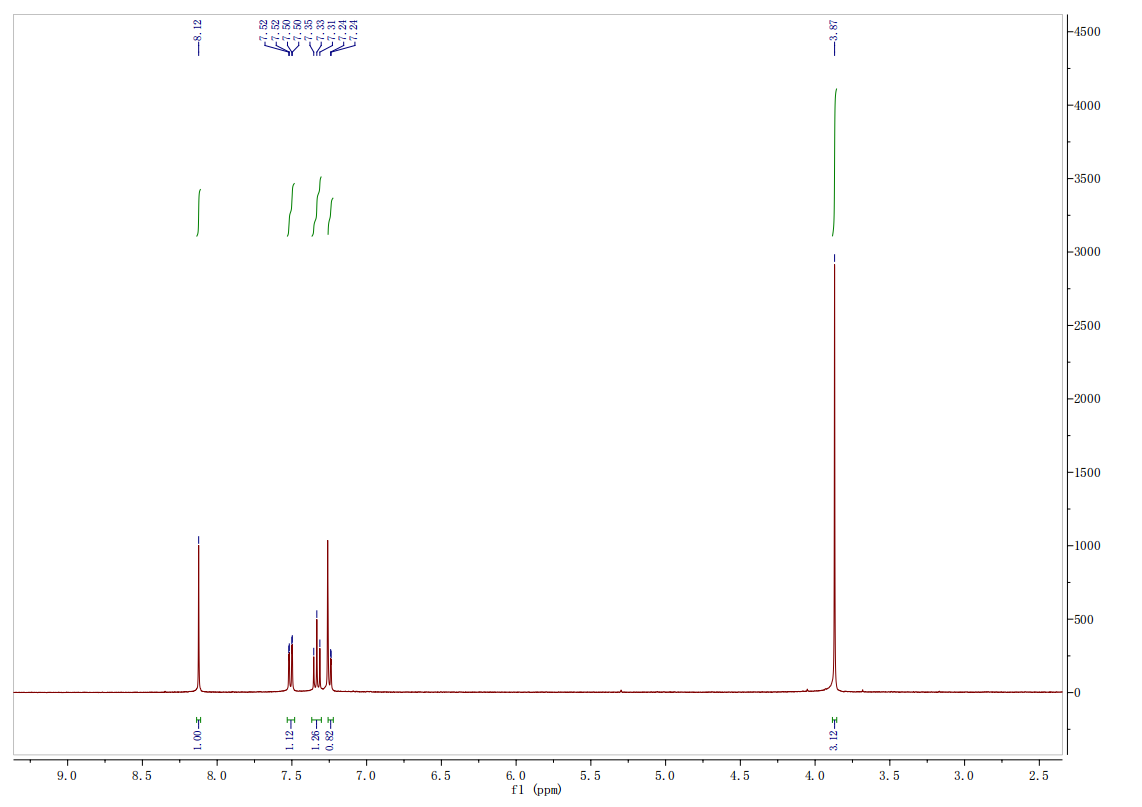
**

**
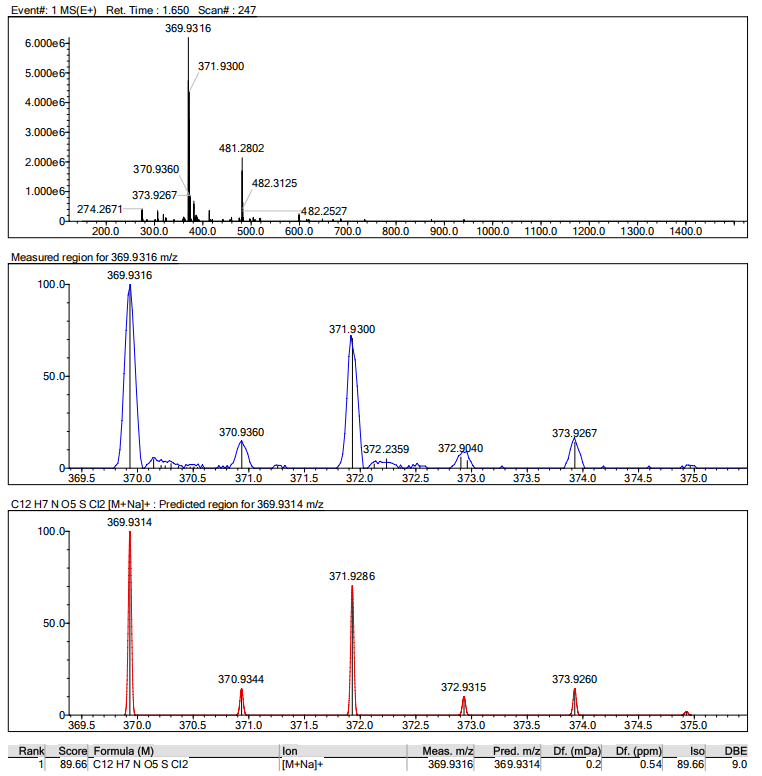
**

**NTP17**

**
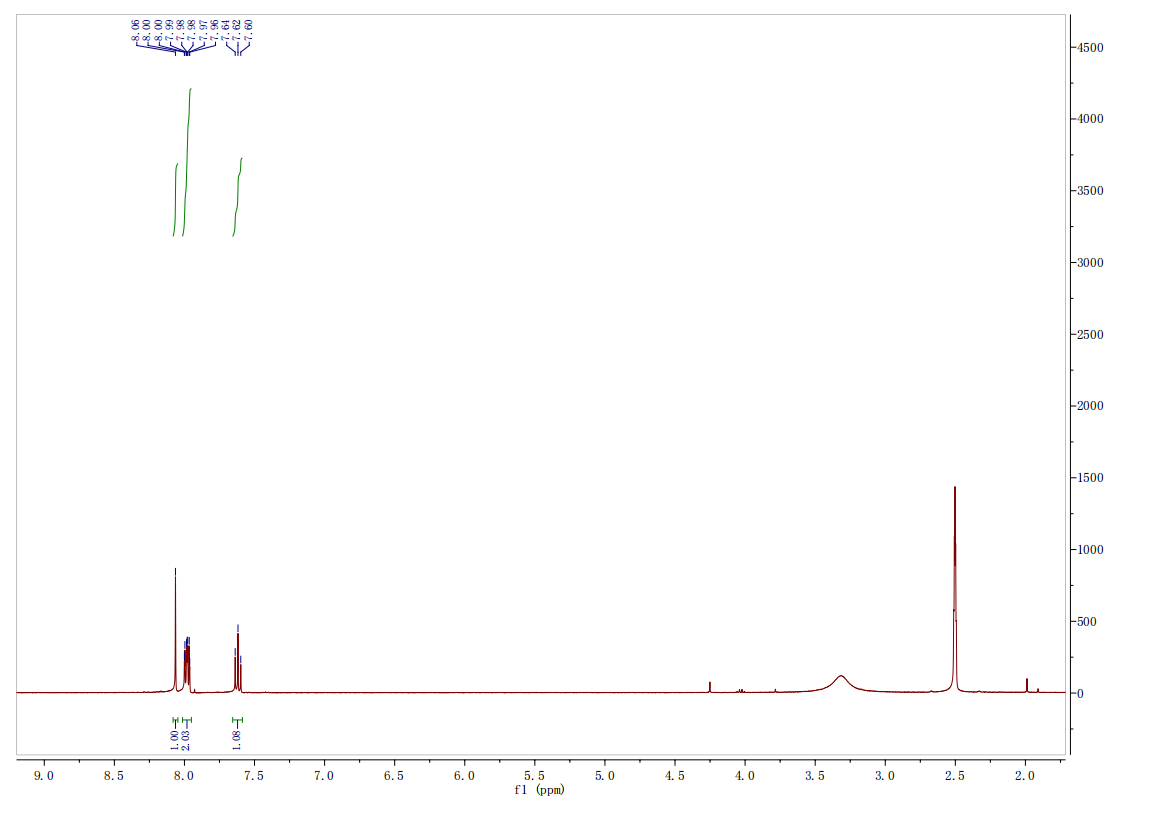
**

**
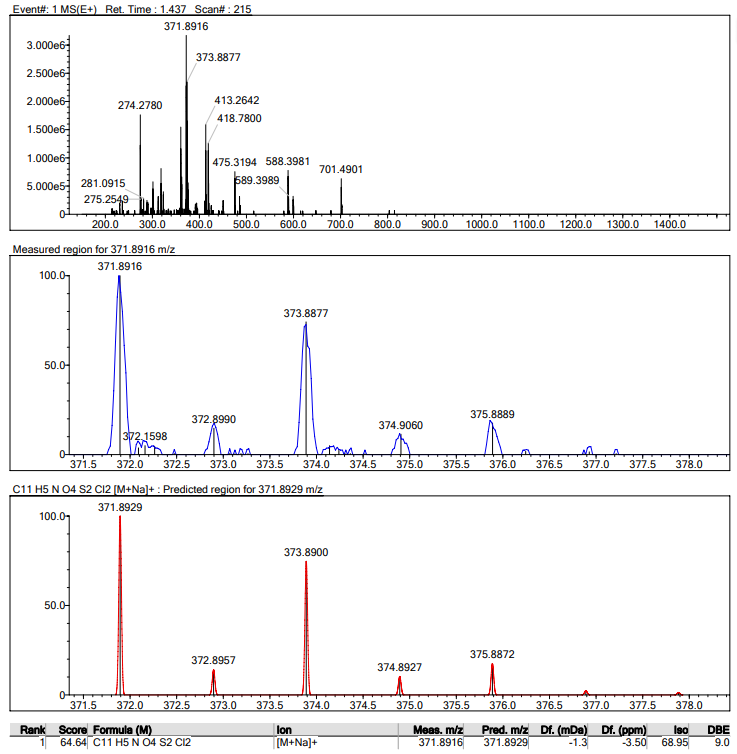
**

**NTP18**

**
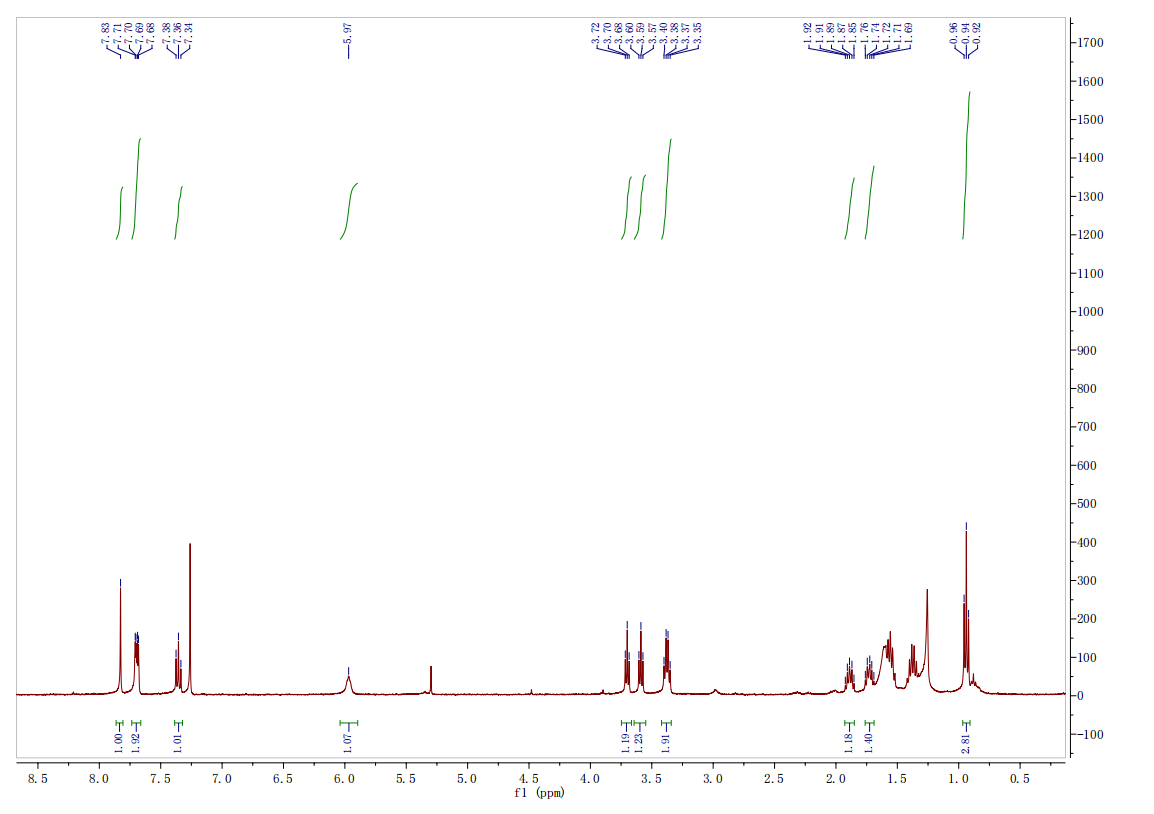
**

**
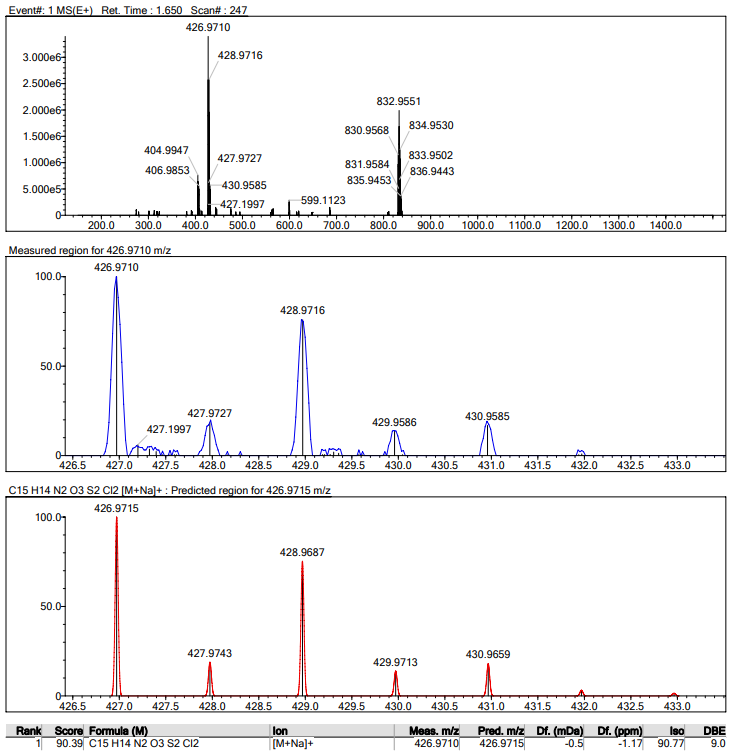
**

**NTP19**

**
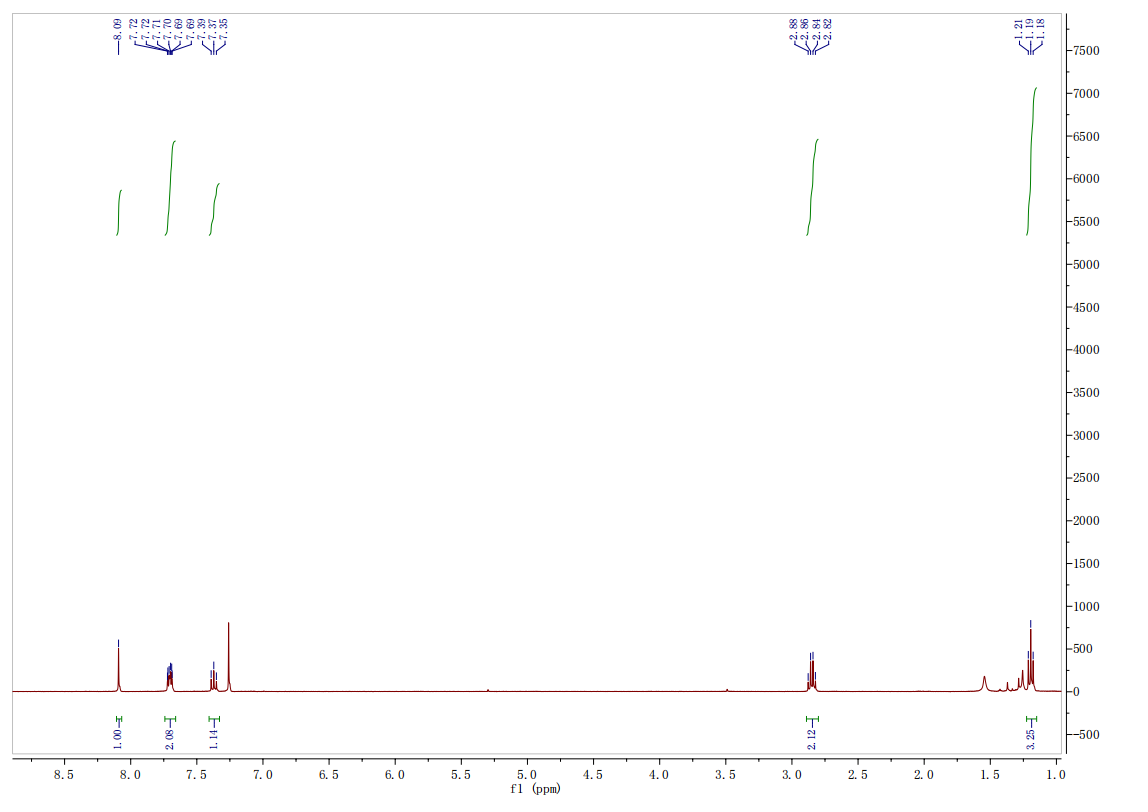
**

**
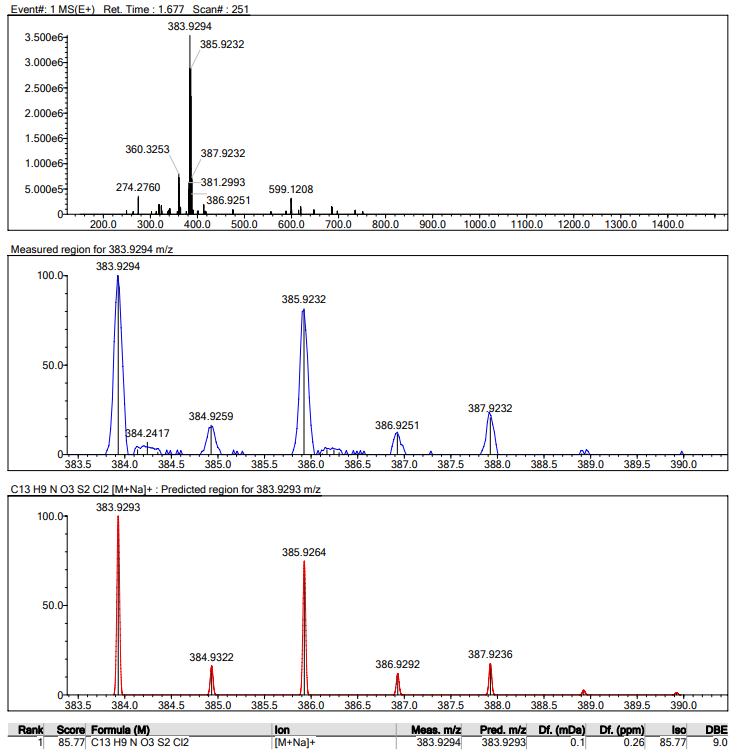
**

**NTP20**

**
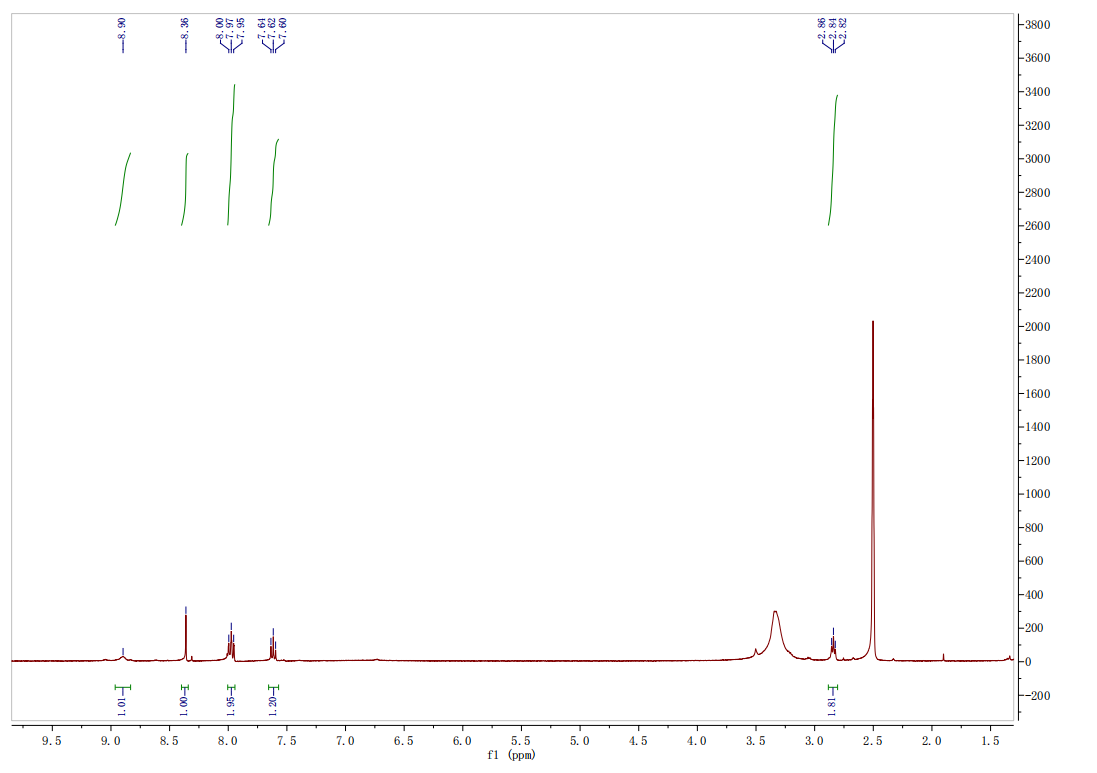
**

**
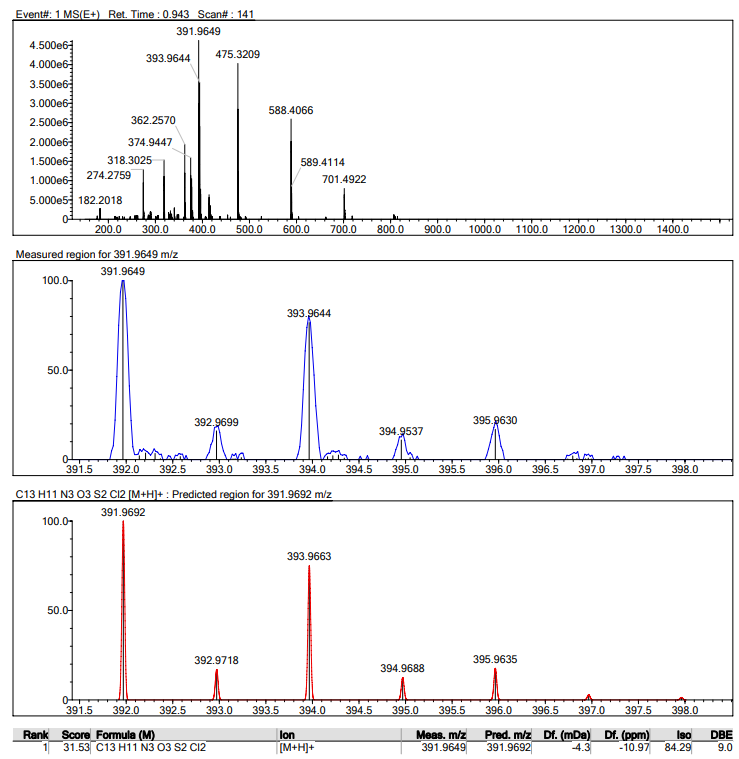
**

**NTP21**

**
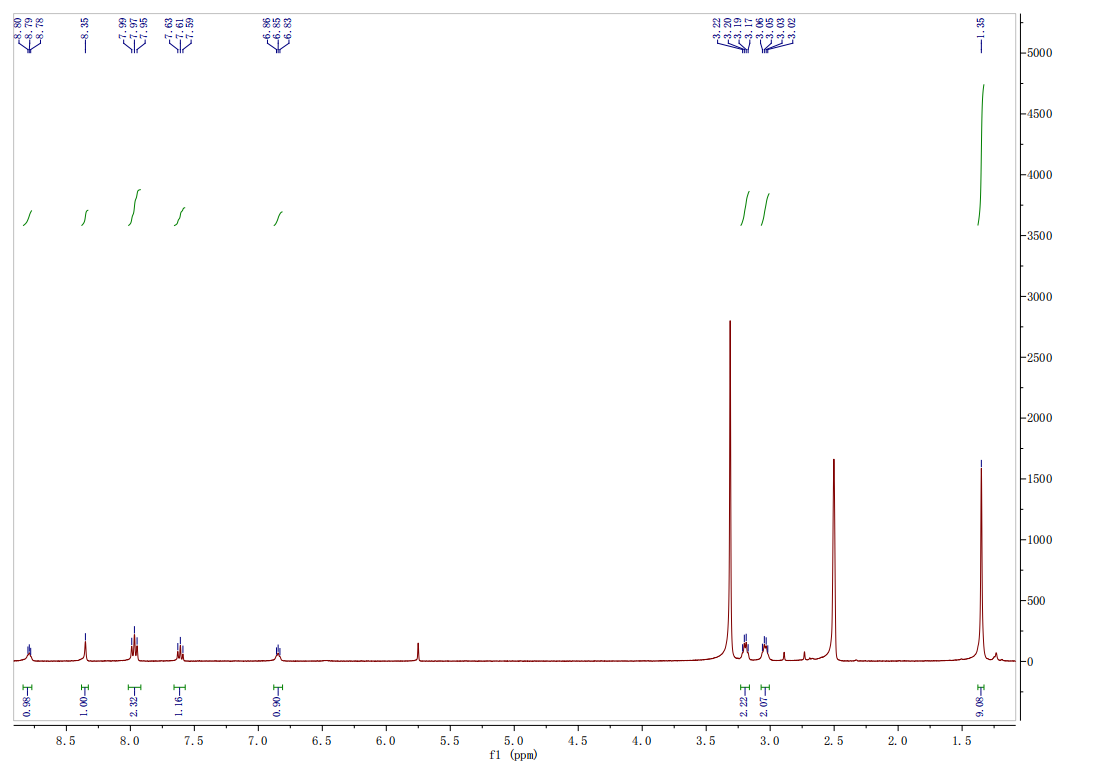
**

**
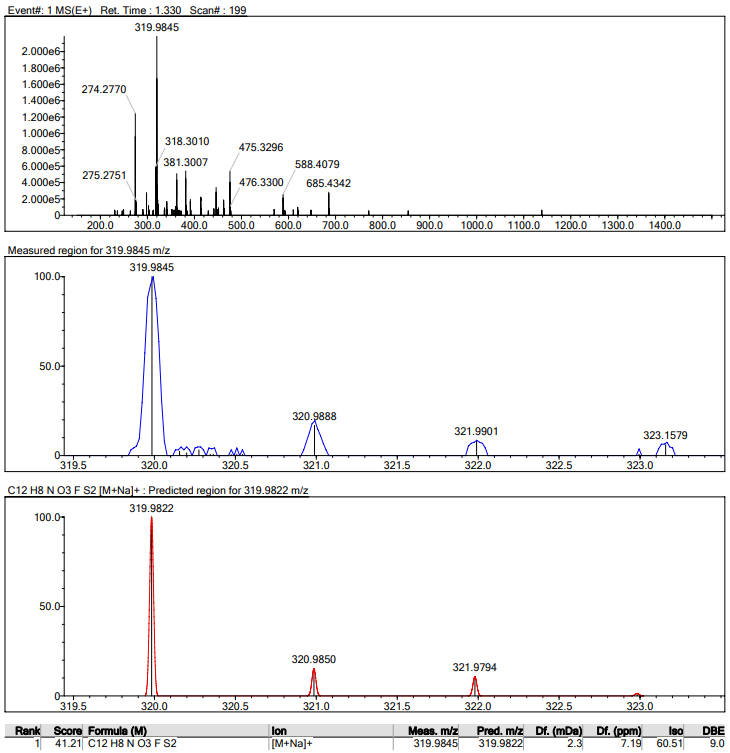
**

**P005091-Biotin**

**
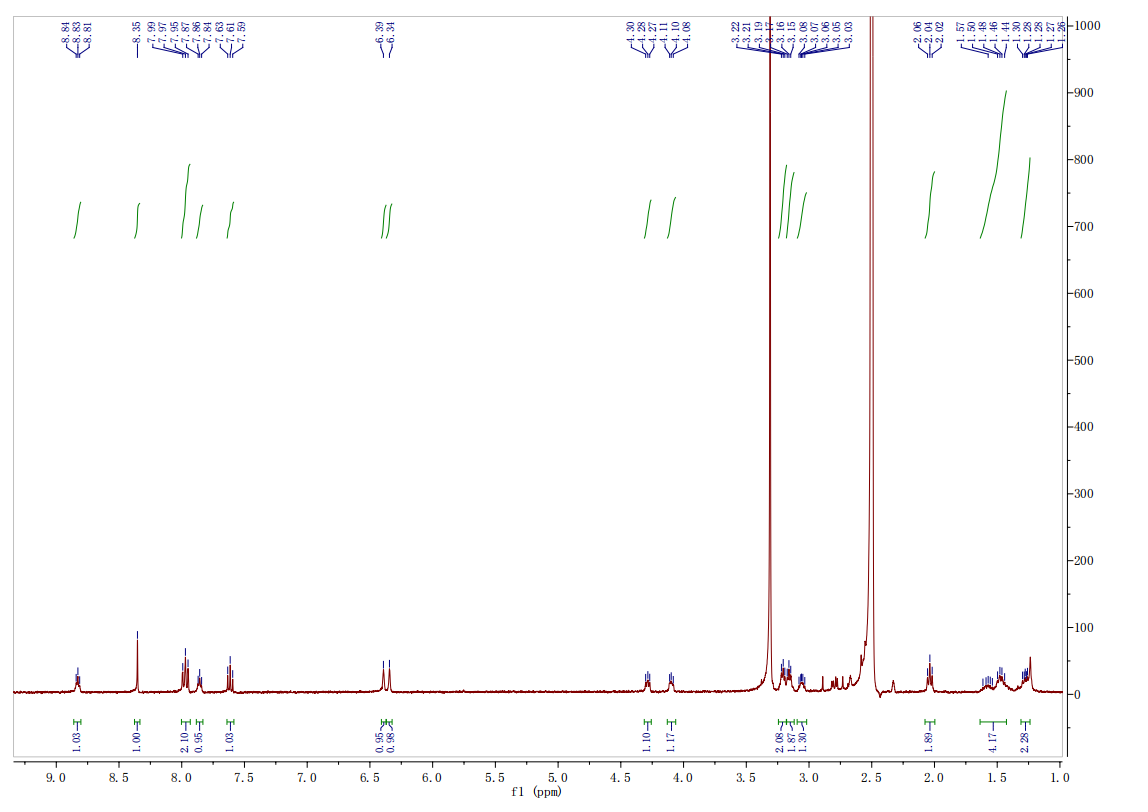
**

**
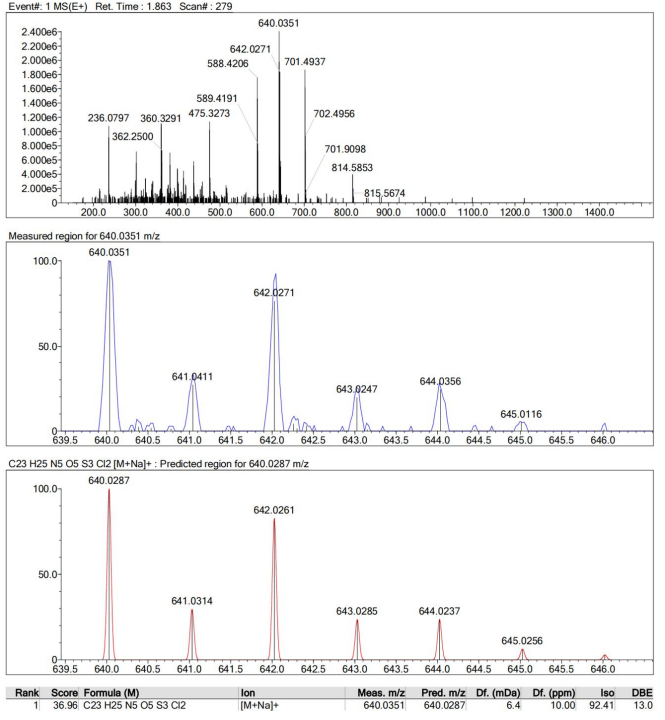
**
